# Analysis of study Global Burden of Disease in 2021: global, regional, and national burden of nutritional deficiency from 1990 to 2021

**DOI:** 10.3389/fnut.2024.1540485

**Published:** 2025-01-15

**Authors:** Jing Chen, Zedong Li, Hong Liu

**Affiliations:** ^1^Department of Surgery, Institute of Integrated Traditional Chinese and Western Medicine, Frontiers Science Center for Disease-related Molecular Network, West China Hospital, Sichuan University, Chengdu, China; ^2^Department of Gastrointestinal Surgery, Xiangya Hospital, Central South University, Changsha, China; ^3^Department of Surgery, Integrated Traditional and Western Medicine, West China Hospital, Sichuan University, Chengdu, China

**Keywords:** nutritional deficiencies, GBD, DALYs, trend projections, Social Development Index

## Abstract

**Background:**

Nutrient deficiency disorders (NDs) harm growth, causing economic losses. Addressing NDs is a global priority, yet recent data is limited. This study examines latest NDs data across 204 countries and 21 regions from 1990 to 2021.

**Methods:**

Data from the 2021 Global Burden of Disease (GBD) study were used to analyze NDs-related incidence, prevalence, deaths, and disability-adjusted life years (DALYs) at global, national, and regional levels. Joinpoint regression analysis was applied to evaluate temporal trends, with Estimated Annual Percentage Change (EAPC) assessing long-term patterns.

**Results:**

In 2021, the global burden of NDs remained substantial, with a total of 1,845,246,558 cases with an ASPR of 23,858.99 cases per 100,000 individuals (95% UI: 23,445.77–24,320.82). The ASIR was 7,725.1 per 100,000 people (95% UI: 7,404.01–8,109.01), while the ASMR was 3.03 per 100,000 persons (95% UI: 2.69–3.4). Additionally, age-standardized DALYs rate was 657.62 per 100,000 individuals (95% UI: 489.93–869.58). Regionally, areas with low SDI exhibited the greatest ASPR, ASIR, ASDR, and age-standardized DALYs rates, whereas high SDI regions had the lowest rates.

**Conclusions:**

Although global NDs burden has declined from 1990 to 2021, regional and demographic disparities remain. Enhanced healthcare access in high-risk SDI regions is essential to further mitigate NDs's global impact.

## 1 Introduction

Nutrition provides the essential energy needed for the body to perform vital functions. A lack of adequate nutrition can not only impair growth but also lead to the loss of various bodily functions and increase the risk of disease (1), Nutrient deficiencies (NDs) occur when there is an insufficient intake or absorption of essential nutrients, and they contribute significantly to the global disease burden (2). In recent years, nutritional deficiencies has quietly reached pandemic proportions (3). Meta-analyses indicate that NDs prevalence among high-risk populations ranges from 5.8% to 28.0%, spanning communities to hospital settings (4–6). Nutrition plays a crucial role in health, particularly for vulnerable groups such as the elderly, pregnant women, newborns, and children (7). Studies consistently show that nutritional deficiencies and the risk of nutritional deficiencies are associated with increased mortality in these populations (8–10). As such, nutrient deficiencies are increasingly recognized as a serious public health concern, requiring more focused attention from policymakers.

Research in this area has broadened from older adults in hospitals, nursing facilities, and nursing homes to include community settings (11). However, surveys remain limited to district-level data, reflecting disparities in health resources. A recent study analyzed cross-border health inequalities in children's nutritional status from 1990 to 2019, using a global nutrition index based on disability-adjusted life years (DALYs) (12). DALYs measure the impact of nutrient deficiencies by combining years of life lost (YLL) due to premature death and years lived with disability (YLD) (13). These studies emphasize the unequal status of nutritional deficiencies. Currently, there is still a lack of a global analysis of nutritional deficiencies across all age groups, regions, and areas.

This study aims to explore how geographic location, Social Development Index (SDI), age, and gender influence global trends in NDs prevalence, mortality, and DALYs, utilizing GBD data from 204 countries and territories between 1990 and 2021.

## 2 Materials and methods

### 2.1 Research design

This study covers the data of GBD incidence rate, prevalence, death, DALYs and their time trends in 204 countries or regions and 21 regions from 1990 to 2021. It has observed different trends of NDs burden and significant differences in gender, region, country and sociodemographic indexes.

### 2.2 Data sources

The nutritional deficiencies data analyzed in this study comes from GBD 2021, which provides the latest estimates of the epidemiological burden of 371 diseases and injuries in 21 GBD regions and 204 countries and regions from 1990 to 2021. All of this data can be exchanged through the Global Health Data Exchange (https://ghdx.healthdata.org/gbd-2021/sources) Free access.

### 2.3 The social demographic index (SDI)

SDI is a comprehensive indicator introduced by the Institute for Health Metrics and Evaluation (IHME) in 2015 to measure a country's or region's level of development, with a focus on the interplay between social development and population health outcomes. Ranging from 0 to 1, a higher SDI indicates greater socioeconomic development (5, 13). The SDI is known to correlate with disease incidence and mortality rates. In this study, we classified countries and regions into five SDI categories (low, low-medium, medium, medium–high, and high) to examine the relationship between NDs burden and socioeconomic development.

### 2.4 Estimated annual percentage change (EAPC)

The EAPC is an effective and widely used indicator, which has been widely used in previous studies to track the trend of morbidity and incidence rate and other indicators within a specific time period. When assessing the temporal trends, we introdmmluced the indicator of estimated annual percentage change (EAPC) (14, 15). The EAPC was calculated based on a regression model, that is (16):


$$\begin{array}{r} \text{y}=\text{α}+\text{βx}+\varepsilon \\ \text{EAPC}=100\times \left(\text{exp}\left(\text{β}\right)-1\right) \end{array}$$

The 95% confidence interval (CI) of EAPC also comes from this fitting model. The trends were recognized as a decrease when the upper boundary of 95% CI of EAPC was < 0; while if the lower boundary of 95% CI of EAPC was > 0, the upward trends of burden were defined; otherwise, the trends represented stable In addition, this study uses percentage changes to reflect the changes in prevalence, incidence rate and DALYS cases in 2021 compared with 1990 (17).


$$\begin{array}{l} \text{Percentage change}=\left(2021\text{ cases}-1990\text{ cases}\right)/\left(1990\text{ cases}\right) \end{array}$$

### 2.5 Joinpoint regression analysis

In this study, the Joinpoint regression analysis model was used—a statistical method commonly used in epidemiological research to evaluate the temporal trend of disease prevalence or mortality. The study used geometric weights from different regions to calculate the average annual percentage change (AAPC) and corresponding 95% CI to demonstrate the 30-year trend of mortality and incidence rate. When assigning weights to the length of each segment, the specified time interval was taken into account. The formula for AAPC is as follows:


$$\begin{array}{l} \text{AAPC}=\left\{\text{exp}\left(\Sigma \text{wibi}/\Sigma \text{wi}\right)-1\right\}\times 100 \end{array}$$

Where “wi” represents the length of each segment within the annual range, and “bi” is the slope coefficient of each segment within the required annual range. We compared the amplitude of AAPC with zero and concluded that the lack of significance indicates a stable trend, aiming to achieve statistical significance. Select the permutation test model and parameter method for CI. If AAPC is located in a segment, perform t-distribution; Otherwise, apply a normal (Z) distribution. In each computing step, we repeat the calculations 1,000 times, drawing a sample for each parameter in each iteration. Using these 1,000 draws, we compute the final estimate's mean value and 95% Uncertainty Interval (UI). The Uncertainty Interval (UI) is defined as the ordered draws' 25th and 975th values. The uncertainty is due to differences in sample sizes between data sources, data availability, and model settings. The use of UIs rather than CIs allows uncertainty to be propagated to the final estimate (18).

### 2.6 Statistical analysis

Data were described as absolute numbers with 95% uncertainty intervals (UIs) by age, sex, year, state, and drug categories. 95% UI was generated by the 2.5th and 97.5th percentiles of the ordered estimate values for the CODEm process of 1,000 draws (19). Perform all analysis and graphical representation programs using the World Health Organization's Health Equity Assessment toolkit and statistical calculation software R (version 4.4.1).

## 3 Results

### 3.1 Analysis of global and national structural data on nutrient deficiency disorders

Nationally, in 2021, the numbers of NDs related incident cases, prevalent cases, deaths, and DALYs were 58,612.94 (95%UI: 562,143.85, 61,458.60), 184,524.66 (95%UI: 181,194.64, 188,268.3), 22.23 (95%UI: 19.97, 24.76), and 4,891.93 (95%UI: 3,598.61, 6,498.54) million. Meanwhile, the age-standardized rates of incidence, prevalence, mortality, and DALYs (ASIR, ASPR, ASMR, and DALYs) of the NDs were 7,725.1 (95%UI: 7,404.01, 8,109.01), 23,858.99 (95%UI: 23,445.77, 24,320.82), 3.03 (95%UI: 2.69, 3.4), and 657.62 (95%UI: 489.93, 869.58) per 100,000, respectively. From 1990 to 2021, their EAPCs were −2.52% (95% UI: −2.67%, −2.38%), −0.98% (95% UI: −0.99%, −0.96%), −4.41% (95% UI: −4.84%, −3.98%), and −2.52% (95% UI: −2.71%, −2.32%) (Table 1).

**Table 1:** Incidence, prevalence, deaths, and DALYs of nutritional deficiencies (NDs) in cases and age-standardized rates for both sexes combined in 1990 and 2021.

**Location**	**Measure**	**1990**		**2021**		**1990–2021 EAPC**
		**All-ages cases (both sexes)**	**Age-standardized rates per 100,000 people**	**All–ages cases (both sexes)**	**Age-standardized rates per 100,000 people**	
		***n* (95% UI)**	***n* (95% UI)**	***n* (95% UI)**	***n* (95% UI)**	***n* (95% UI)**
**Sociodemographic index**						
Global	Incidence	964,881,696 (928,560,815–1,001,236,465)	171,12.55 (16,470.31–17,731.43)	586,129,387 (562,138,520–614,585,958)	7,725.1 (7,404.01–8,109.01)	−2.52 (−2.67 to −2.38)
	Prevalence	1,765,110,763 (1,735,880,718–1,794,376,240)	32217.95 (31693.55–32740.92)	1,845,246,558 (1,811,946,448–1,882,683,059)	23,858.99 (23,445.77–24,320.82)	−0.98 (−0.99 to −0.96)
	Deaths	570,119 (485,777–693,881)	10.9 (9.44–12.97)	222,274 (199,731–247,630)	3.03 (2.69–3.4)	−4.41 (−4.84 to −3.98)
	DALYs	78,674,224 (64,859,007–97,375,904)	1,367.15 (1,126.3–1,708.49)	48,919,261 (35,986,110–64,985,437)	657.62 (489.93–869.58)	−2.52 (−2.71 to −2.32)
High SDI	Incidence	24,546,126 (22,463,966–26,835,801)	2,909.5 (2668.01–3,180.88)	19,575,040 (17,266,355–22,245,938)	1,671.71 (1,484.68–1,885.64)	−1.42 (−1.59 to −1.26)
	Prevalence	79,096,229 (75,591,321–82,908,356)	9118.68 (8732.9–9,522.94)	74,228,337 (70,673,385–77,972,862)	6,458.84 (6,173.06–6,781.27)	−0.91 (−1.02 to −0.81)
	Deaths	7,797 (7,003–8,275)	0.78 (0.7–0.84)	24,537 (20,287–26,826)	0.95 (0.8–1.03)	0.21 (−0.28 − 0.71)
	DALYs	1,109,206 (782,906–1,605,007)	131.07 (92.53–189.66)	1,517,919 (1,120,271–2,055,320)	118.26 (83.05–165.11)	−0.07 (−0.17 −0.04)
Middle SDI	Incidence	269,727,153 (256,230,128–284,449,670)	14,567.32 (13,862.76–15,328.32)	117,311,987 (110,221,589–125,794,283)	5,016.29 (4,705.26–5,381.24)	−3.28 (−3.38 to −3.18)
	Prevalence	535,577,106 (522,058,155–549,984,341)	30,487.47 (29,726.7–31,238.93)	458,836,864 (447,289,272–471,001,855)	19,136.37 (18,700.19–19,617.53)	−1.48 (−1.5 to −1.46)
	Deaths	109,572 (101,308–119,935)	9.89 (9.09–10.56)	53,543 (48,304–57,698)	2.62 (2.34–2.82)	−4.26 (−4.32 to −4.19)
	DALYs	16,827,001 (13,719,008–21,383,808)	972.77 (797.32–1229.51)	10,513,020 (7,538,644–14,390,371)	459.47 (331.59–624.72)	−2.39 (−2.42 to −2.35)
Low SDI	Incidence	230,001,057 (223,728,220–236,755,376)	42,587.56 (41,451.32–43,795.16)	225,651,527 (218,004,944–233,508,668)	19,047.59 (18,448.14–19,697.06)	−2.67 (−2.89 to −2.45)
	Prevalence	321,458,652 (316,898,242–325,886,013)	62,945.48 (62,062.97–63,775.33)	496,951,266 (486,858,432–506,781,212)	44,208.88 (43,375.1–45,081.24)	−1.2 (−1.27 to −1.13)
	Deaths	207,305 (161,460–270,528)	35.05 (28.92–43.14)	78,101 (61,628–95,120)	8.73 (7.44–10.02)	−4.36 (−4.98 to −3.73)
	DALYs	23,628,234 (19,283,997–29,778,931)	3,334.19 (2,727.78–4,117.71)	16,170,549 (12,342,103–20,971,971)	1,319.28 (1,002.38–1,722.91)	−2.97 (−3.31 to −2.62)
High-middle SDI	Incidence	87,788,129 (81,809,640–94,442,969)	8,339.4 (7,781.3–8,950.07)	41,476,684 (38,159,757–45,288,134)	3,305.31 (3,046.36–3,589.94)	−2.87 (−2.96 to −2.78)
	Prevalence	215,302,592 (208,375,347–221,905,661)	20,406.6 (19,771.83–21,028.35)	156,142,864 (150,977,524–162,351,321)	12,006.92 (11,625.11–12,455.64)	−1.77 (−1.81 to −1.72)
	Deaths	17,140 (15,542–18,832)	2.06 (1.86–2.26)	13,681 (11,883–15,159)	0.8 (0.7–0.89)	−3.26 (−3.58 to −2.95)
	DALYs	4,316,932 (3,243,552–5,836,365)	429.22 (326.5–575.07)	2,592,001 (1,799,258–3,685,988)	203.41 (140.77–288.96)	−2.64 (−2.73 to −2.54)
Low-middle SDI	Incidence	351,995,175 (334,313,603–368,810,119)	27,944.93 (26,526.37–29,160.78)	181,642,679 (171,302,454–193,219,091)	9,389.32 (8,870.92–9,951.09)	−3.48 (−3.64 to −3.32)
	Prevalence	612,148,578 (600,041,562–624,013,702)	51,757.29 (50,783.71–52,700.59)	657,750,319 (644,770,854–671,669,215)	34,604.42 (33,967.95–35,312.84)	−1.31 (−1.32 to −1.29)
	Deaths	227,994 (191,098–275,044)	19.6 (16.61–22.99)	52,234 (46,202–58,902)	3.73 (3.33–4.12)	−6.05 (−6.72 to −5.37)
	DALYs	32,747,125 (26,960,542–40,530,876)	2,374.12 (1,965.67–2,980.94)	18,094,875 (13,077,187–24,551,130)	971.02 (706.71–1,308.72)	−3.17 (−3.41 to −2.93)
**Region**						
Central Asia	Incidence	6,498,243 (6,075,993–6,929,210)	8,894.18 (8,339.6–9,462.22)	4,749,728 (4,423,462–5095799)	4,927.2 (4,597.18–5,281.99)	−1.9 (−2.04 to −1.75)
	Prevalence	22,334,239 (21,598,359–23,179,108)	30,957.07 (29,949.41–31,979)	23,804,197 (22,785,372–25,092,731)	24,557.43 (23,510.4–25,908.38)	−0.85 (−0.92 to −0.79)
	Deaths	901 (824–987)	1.23 (1.13–1.33)	275 (239–318)	0.32 (0.28–0.36)	−5.18 (−5.59 to −4.77)
	DALYs	637,553 (453,032–885,816)	845.24 (595.76–1,176.9)	571,576 (390,226–816,401)	585.12 (399.86–837.14)	−1.49 (−1.61 to −1.37)
East Asia	Incidence	145,728,245 (128,205,577–164,193,031)	1,1535.28 (10,239.38–12,956.89)	47,953,796 (42,789,520–53,591,198)	3,450.57 (3,075.48–3,859.46)	−3.7 (−3.83 to −3.57)
	Prevalence	281,133,227 (265,234,703–298,969,903)	22,973.86 (21,774.63–24,315.05)	152,264,619 (144,553,425–160,840,133)	9,993.97 (9,525.91–10,550.2)	−2.72 (−2.78 to −2.66)
	Deaths	38,161 (33,059–43,798)	29.27 (4.78–6.1)	16,452 (13,635–19,383)	1.13 (0.93–1.32)	−8.07 (−10.13 to −5.97)
	DALYs	6,372,105 (5,103,034–8,191,738)	570.83 (462.24–725.04)	2,398,822 (1,637,228–3,456,190)	160.76 (110.46–228.34)	−6.17 (−7.52 to −4.8)
South Asia	Incidence	332,392,143 (306,306,678–356,480,485)	28,292.51 (26,126.08–30,300.72)	166,181,992 (150,406,214–183,692,446)	9,171.97 (8,326.93–10,138.25)	−3.59 (−3.83 to −3.35)
	Prevalence	634,104,800 (617,323,641–650,116,133)	57,698.06 (56,207.27–59,040.58)	739,051,761 (719,938,200–759,461,377)	40,854.69 (39,854.44–41,943.76)	−1.13 (−1.15 to −1.11)
	Deaths	21,4969 (174,958–260,197)	19.49 (16.02–23.18)	35,229 (29,438–41,650)	2.56 (2.16–3)	−6.23 (−6.4 to −6.06)
	DALYs	35,096,798 (28,574,752–44,285,658)	2,796.88 (2,246.58–3,574.34)	2,077,8261 (14,607,575–28,656,309)	1,187.87 (840.68–1,627.64)	−2.69 (−2.72 to −2.67)
Southeast Asia	Incidence	103,870,040 (97,630,098–110,410,060)	19,895.08 (18,775.33–21,118.15)	38,912,936 (36,375,564–41,989,770)	5,892.56 (5,501.48–6,347.38)	−3.74 (−3.83 to −3.66)
	Prevalence	172,103,477 (166,521,018–178,061,548)	35,834.3 (34,722.31–36,946.67)	137,595,402 (133,035,023–142,160,406)	20,247.73 (19,580.2–20,906.14)	−1.83 (−1.91 to −1.76)
	Deaths	41,160 (34,178–49,604)	15.04 (12.61–17.05)	29,018 (24,952–32,476)	5.96 (5.1–6.65)	−2.8 (−2.94 to −2.66)
	DALYs	5,143,487 (4,134,119–6,512,203)	1,104.99 (899.41–1,380.21)	3,255,847 (2,427,929–4,327,584)	505.55 (384.3–664.82)	−2.49 (−2.58 to −2.4)
Central Europe	Incidence	21,044,355 (20,102,527–22,174,741)	17,286.89 (16,504.76–18,203.53)	8,321,411 (7,862,904–8,811,424)	7,479.7 (7,086.41–7,915.69)	−2.81 (−2.92 to −2.7)
	Prevalence	36,725,051 (35,598,638–37,811,774)	30,101.36 (29,150.76–31,017.93)	19,224,722 (18,602,276–20,012,743)	17,361.46 (16,753.07–18,164.11)	−1.87 (−1.91 to −1.82)
	Deaths	194 (181–210)	0.17 (0.15–0.18)	747 (679–810)	0.36 (0.33–0.39)	1.89 (1.32 −2.47)
	DALYs	428,680 (283,570–623,528)	360.34 (237.09–521.6)	224,408 (155,096–323,237)	200.85 (134.94–293.55)	−2.04 (−2.13 to −1.95)
Eastern Europe	Incidence	4,057,741 (3,573,017–4,615,030)	1,891.24 (1,660.15–2,160.48)	2,171,095 (1,837,608–2,532,989)	1,146.35 (947.99–1,355.48)	−1.25 (−1.42 to −1.09)
	Prevalence	32,667,546 (30,870,116–34,777,512)	14,534.95 (13,740.49–15,390.8)	23,276,071 (21,637,366–25,071,286)	11,128.59 (10,414.49–11,888.5)	−0.99 (−1.11 to −0.87)
	Deaths	1,106 (1,066–1,143)	0.48 (0.46–0.5)	878 (817–938)	0.29 (0.27–0.31)	−3.12 (−4.1 to − 2.13)
	DALYs	774,337 (527,315–1,115,780)	352.98 (242.19–508.14)	481,404 (337,108–685,573)	236.53 (163.1–337.2)	−1.63 (−1.82 to −1.44)
Western Europe	Incidence	8,775,025 (8,051,814–9,684,894)	2,386.76 (2,195.93–2,632.51)	8,279,770 (7,236,188–9,518,797)	1,681 (1,481.09–1,911.45)	−0.5 (−0.72 to −0.29)
	Prevalence	31,325,135 (29,207,067–33,800,039)	8324.04 (7774.86–8963.74)	27764099 (25925376–30162538)	5987.26 (5563.01–6531.68)	−0.88 (−1 to −0.76)
	Deaths	3,582 (3,168–3,860)	0.67 (0.59–0.72)	8620 (6954–9697)	0.66 (0.54–0.74)	0.01 (−0.17 − 0.19)
	DALYs	386,946 (259,997–577,480)	105.9 (69.93–158.02)	487,327 (328,126–721,785)	94.55 (59.19–138.75)	−0.11 (−0.27 −0.04)
Australasia	Incidence	189,489 (160,872–226,058)	915.79 (777.01–1,096.17)	301,024 (265,789–353,778)	845.61 (741.33–996.47)	0.03 (−0.18 −0.24)
	Prevalence	1,158,776 (1,010,770–1,459,270)	5,871.86 (4,990.21–7,849.45)	1,459,598 (1,318,169–1,709,489)	4,586.74 (3,994.95–5,902.02)	−0.72 (−0.79 to −0.65)
	Deaths	84 (76–90)	0.4 (0.36–0.43)	164 (137–182)	0.26 (0.22–0.29)	−1.62 (−1.92 to −1.32)
	DALYs	14,043 (9,189–20,923)	69.5 (44.71–106.19)	17,763 (11,730–28,238)	53.43 (33.98–87.56)	−0.85 (−0.97 to −0.73)
Caribbean	Incidence	4,260,123 (4,016,037–4,507,337)	11,640.01 (10,974.12–12,278.43)	3,033,764 (2,804,571–3,295,404)	6,591.5 (6,080.1–7,179.38)	−2 (−2.06 to −1.94)
	Prevalence	10,377,561 (10,049,815–10,735,796)	28,665.34 (27,799.97–29,612.38)	11,389,503 (10,962,546–11,940,346)	24,673.9 (23,720.07–25,891.38)	−0.53 (−0.62 to −0.44)
	Deaths	4,001 (3,354–4,789)	11.13 (9.52–13.1)	1,881 (1,517–2,389)	4.14 (3.28–5.37)	−3.04 (−3.42 to −2.65)
	DALYs	515,707 (422,279–635,094)	1,323.48 (1,080.53–1,634.01)	325,160 (239,143–437,026)	746.86 (553.64–1,001.03)	−1.73 (−1.96 to −1.49)
Oceania	Incidence	1,339,378 (1,252,388–1,429,921)	18,443.43 (17,325.88–19,591.87)	1,586,342 (1,451,597–1,741,349)	10,329.04 (9,522.33–11,202.87)	−1.52 (−1.67 to −1.38)
	Prevalence	2,551,814 (2,411,742–2,721,367)	37,722.84 (35,921.84–39,771.06)	4,248,613 (3,881,670–4,672,495)	29,695.81 (27,374.8–32,536.36)	−0.63 (−0.7 to −0.57)
	Deaths	238 (190–297)	7.58 (6.3–9.1)	293 (227–378)	4.36 (3.57–5.42)	−1.84 (−1.88 to −1.79)
	DALYs	53,516 (40,032–71,376)	776.54 (583.49–1037.62)	88,158 (61,801–132,058)	610.45 (433.75–897.37)	−0.54 (−0.62 to −0.46)
Andean Latin America	Incidence	4,324,050 (3,968,542–4,719,109)	10,346.99 (9,529.53–11,252.21)	3,300,572 (3,023,871–3,603,401)	4,975.44 (4,562.47–5,432.15)	−2.7 (−2.94 to −2.46)
	Prevalence	10,484,115 (9,725,704–11,391,468)	25,745.5 (23,999.1–27,790.92)	9,760,590 (9,070,654–10,669,803)	14,811.91 (13,803.47–16,211.18)	−1.99 (−2.06 to −1.92)
	Deaths	6,899 (5,967–7,966)	21.84 (19.44–24.48)	2,985 (2,471–3,615)	5.24 (4.34–6.34)	−4.95 (−5.19 to −4.7)
	DALYs	625,218 (518,413–744,687)	1,396.57 (1,181.12–1,646.63)	237,065 (182,191–305,227)	375.99 (290.86–481.58)	−4.48 (−4.67 to −4.29)
Central Latin America	Incidence	23,202,044 (21,606,348–24,999,916)	13,013.43 (12,134.32–13,917.95)	13,940,960 (12,822,617–15,237,964)	5,594.27 (5,148.05–6,102.81)	−2.55 (−2.64 to −2.47)
	Prevalence	37,544,533 (35,853,779–39,239,515)	21,405.79 (20,563.37–22,311.79)	31,383,870 (30,058,891–32,681,022)	12,701.93 (12,187.02–13,237.35)	−1.61 (−1.64 to −1.57)
	Deaths	25,568 (24,495–26,672)	25.63 (24.48–26.43)	11,380 (10,189–12,677)	4.94 (4.42–5.51)	−5.43 (−5.53 to −5.33)
	DALYs	1,758,748 (1,587,513–1,987,774)	1037.87 (948.91–1154.78)	712,763 (576,156–897,813)	303.26 (246.92–380.03)	−3.94 (−4.05 to −3.83)
Tropical Latin America	Incidence	36,479,450 (33,271,238–40,093,411)	23,477.83 (21,509.41–25,637.81)	22,986,968 (20,379,717–26,123,602)	10,148.82 (8,997.31–11,433.6)	−2.78 (−2.87 to −2.69)
	Prevalence	58,726,397 (55,256,855–62,407,938)	37,996.29 (35,895.89–40,380.31)	51,846,394 (47,957,201–55,694,676)	22,995.86 (21,309.36–24,743.73)	−1.67 (−1.7 to −1.65)
	Deaths	12,777 (11,838–13,783)	11.02 (10.27–11.73)	6,224 (5,453–6,713)	2.63 (2.3–2.84)	−4.7 (−4.99 to −4.41)
	DALYs	1,760,950 (1,445,446–2,164,069)	1,131.66 (940.38–1,387.23)	968,433 (685,625–1,330,150)	446.57 (315.59–611.6)	−3.16 (−3.27 to −3.05)
Southern Latin America	Incidence	5,534,434 (4,999,689–6,146,388)	11,027.26 (9,963.22–12,266.04)	4,151,693 (3,685,485–4,574,474)	6,323.94 (5,561.38–6,960.63)	−1.67 (−1.92 to −1.43)
	Prevalence	9,387,604 (8,412,555–10,661,326)	18,876.02 (16,957.74–21,371.29)	7,990,908 (6,837,460–9,742,035)	12,388.91 (10,501.75–15,136.29)	−1.27 (−1.35 to −1.19)
	Deaths	1,944 (1,853–2,039)	4.3 (4.08–4.51)	1,491 (1,315–1,618)	1.69 (1.5–1.83)	−2.96 (−3.59 to − 2.33)
	DALYs	158,223 (131,463–196,834)	320.87 (267.36–398.1)	78,615 (54,851–115,525)	116.33 (79.43–172.14)	−3.2 (−3.4 to −3)
High-income Asia Pacific	Incidence	3,694,595 (3,257,539–4,225,776)	2,330.42 (2,051.34–2,650.66)	2,546,370 (2,219,509–2,972,885)	1,384.11 (1,214.58–1,602.14)	−1.46 (−1.59 to −1.34)
	Prevalence	15,173,415 (13,504,296–17,218,684)	8,927.27 (8,013.99–10,074.53)	12,515,714 (11,273,662–14,208,106)	5,950.02 (5,287.5–6,845.45)	−1.17 (−1.31 to −1.02)
	Deaths	1,075 (982–1,141)	0.63 (0.57–0.68)	2443 (2018–2703)	0.44 (0.38–0.47)	−1.26 (−1.4 to − 1.12)
	DALYs	146,204 (94,882–223,583)	86.22 (55.97–133.63)	154,630 (107,984–219,003)	60.3 (40.08–91.05)	−1.03 (−1.16 to −0.89)
High-income North America	Incidence	5,378,090 (4,581,285–6,312,368)	1,442.25 (1,218.3–1,695.86)	5,871,693 (4,891,131–7,010,324)	1,931.2 (1,650.96–2,254.47)	−0.76 (−1.05 to −0.48)
	Prevalence	16,298,514 (15,102,006–17,479,238)	5,670.76 (5,277.49–6,082.16)	20,313,302 (18,658,729–22,271,772)	5,125.24 (4,694.11–5,591.68)	−0.1 (−0.21 −0.01)
	Deaths	2,369 (2,081–2,513)	0.65 (0.57–0.69)	13,502 (11,301–14,711)	1.83 (1.55–1.98)	2.51 (1.64 − 3.38)
	DALYs	223,783 (146,083–343,977)	74.54 (48.14–115.68)	639,568 (478,816–841,150)	136.95 (99.2–185.49)	2.2 (1.94 −2.47)
Eastern Sub-Saharan Africa	Incidence	96,784,365 (94,113,251–99,206,370)	21,823.69 (21,112.3–22,493.6)	93,965,431 (90,500,597–97,701,700)	49,502.75 (48,371.01–50,624.14)	−2.81 (−3.01 to −2.62)
	Prevalence	121,213,649 (118,849,394–123,447,870)	62,941.91 (61,867.39–63,958.65)	170,733,560 (166,269,936–175,406,207)	39,924.67 (38,899.57–40,949.31)	−1.58 (−1.68 to −1.49)
	Deaths	119,298 (93,822–156,123)	58.19 (48.78–70.56)	42,360 (33,761–51,309)	14.07 (12.08–16.2)	−4.51 (−5.4 to −3.61)
	DALYs	11,426,983 (9,185,969–14,528,585)	4,027.09 (3,326.07–5,033.9)	5,735,279 (4,535,287–7,231,087)	4,027.09 (3,326.07–5,033.9)	−3.87 (−4.57 to −3.16)
Southern Sub-Saharan Africa	Incidence	9,818,704 (9,107,991–10,632,703)	7,472.98 (6,864.25–8,169.85)	6,196,018 (5,683,778–6,800,900)	15,651.79 (14,539.82–16,853.75)	−2.2 (−2.29 to −2.11)
	Prevalence	19,125,680 (18,322,312–20,066,055)	33,546.77 (32,258.91–34,972.23)	19,735,678 (18,920,372–20,580,350)	24,205.61 (23,254.68–25,194.45)	−1.01 (−1.05 to −0.97)
	Deaths	7,449 (6,268–8,989)	13.03 (11.35–15.11)	5,743 (4,640–6,999)	8.31 (6.81–9.99)	−0.81 (−1.04 to −0.58)
	DALYs	992,200 (821,072–1,205,771)	1,554.26 (1,276.73–1,910.12)	866,524 (684,644–1,089,205)	1,089.95 (865.1–1,364.73)	−0.64 (−0.83 to −0.46)
Western Sub-Saharan Africa	Incidence	77,110,003 (74,778,092–79,502,456)	36,033.07 (34,996.05–36,992.6)	83,209,354 (79,910,374–86,670,835)	15,398.37 (14,847.81–15,975.94)	−2.72 (−2.81 to −2.63)
	Prevalence	107,840,088 (105,505,628–110,452,943)	52,720.3 (51,569.5–53,927.15)	198,001,824 (192,301,049–204,421,978)	38,270.03 (37,335.88–39,318.13)	−1.04 (−1.06 to −1.02)
	Deaths	50,191 (37,720–68,990)	19.01 (15.24–24.28)	28,101 (20,058–36,319)	6.41 (5.13–7.78)	−3.42 (−3.54 to −3.3)
	DALYs	6,238,640 (4,995,124–8,008,122)	2,149.11 (1,728.04–2,711.32)	6,620,690 (4,927,381–8,770,740)	1,109.8 (820.77–1,479.62)	−2.15 (−2.23 to −2.07)
Central Sub-Saharan Africa	Incidence	22,761,327 (21,351,921–24,189,196)	36,820.85 (34,735.99–38,886.94)	34,376,562 (31,334,297–37,468,949)	23,067.04 (21,152.87–24,805.25)	−1.4 (−1.83 to −0.97)
	Prevalence	34,424,749 (33,307,033–35,637,673)	61,414.59 (59,722–63,172.75)	61,913,266 (58,993,967–65,101,628)	44,824.1 (42,768.74–46,890.42)	−0.94 (−1.17 to −0.71)
	Deaths	23,988 (17,721–34,877)	35.21 (28.18–47.46)	9,213 (6,450–12,630)	10.14 (7.46–13.33)	−4.24 (−4.58 to −3.9)
	DALYs	2,536,795 (1,949,159–3,505,987)	3,065.62 (2,429.43–3,991.66)	1,475,532 (1,077,598–1,999,448)	1,004.31 (738.83–1,354.82)	−3.76 (−4.05 to −3.47)
North Africa and Middle East	Incidence	51,639,851 (49,635,138–53,818,815)	5,480.22 (5,202.93–5,800.61)	34,091,907 (32,339,077–36,111,544)	14,294.83 (13,771.41–14,832.41)	−2.94 (−3.08 to −2.79)
	Prevalence	110,410,392 (107,908,789–113,281,399)	19,514.2 (19,004.69–20,060.55)	120,972,869 (117,686,883–124,467,698)	31,522.61 (30,838.2–32,235.17)	−1.49 (−1.51 to −1.47)
	Deaths	14,166 (11,135–20,219)	4.07 (3.38–5.3)	5,275 (4,462–6,227)	1.28 (1.09–1.49)	−3.87 (−4.04 to −3.7)
	DALYs	3,383,308 (2,615,337–4,436,262)	844.89 (644.14–1,118.31)	2,801,435 (1,987,899–3,873,220)	455.79 (325.84–629.01)	−2.04 (−2.09 to −1.99)

**Figure 1:**
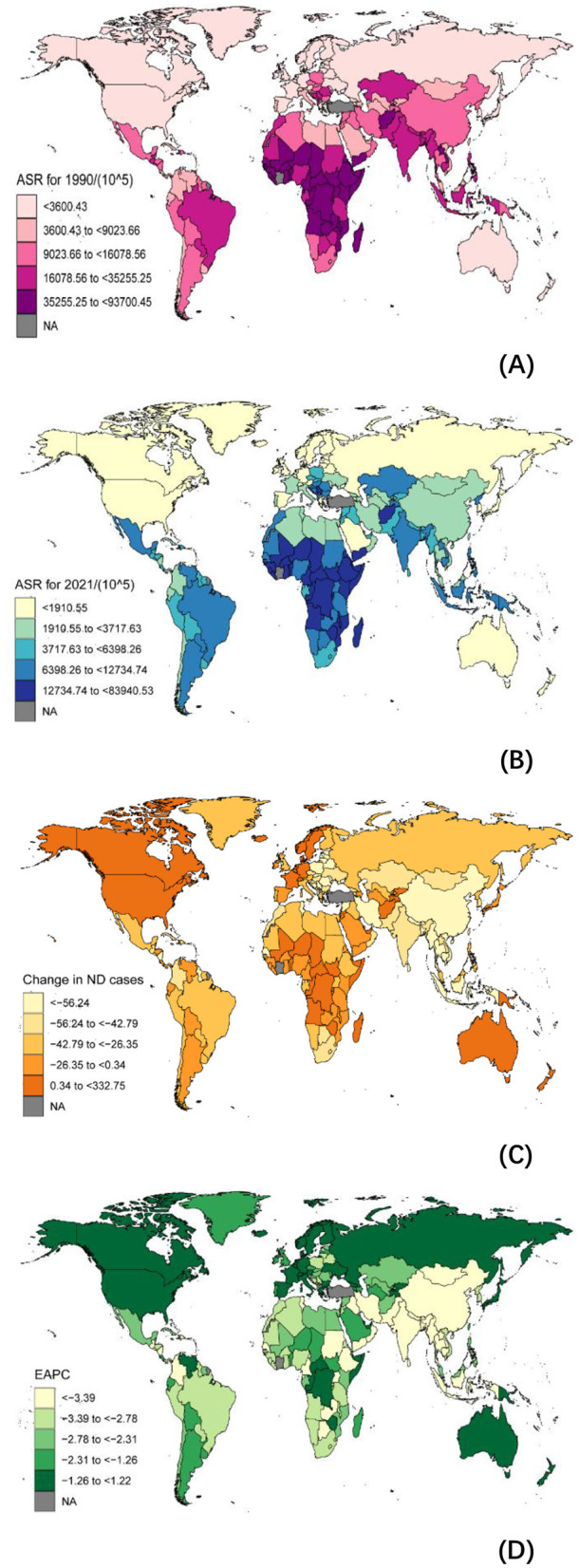
Global Burden of Disease (GBD) and temporal trends of Nutritional Deficiencies (NDs) incidence globally. ASR, age standardized rate; EAPC, estimated Annual Percentage Change; GBD, global burden of disease. **(A)** The ASR per 100,000 people in 1990; **(B)** The ASR per 100,000 people in 2021; **(C)** The change in NDs cases; **(D)** EAPC in different countries or territories.

In the past 30 years, the burden of NDs has significantly decreased globally and in 204 countries (Supplementary Tables S1–S5, Supplementary Figures S1–S3, Figure 1). However, disparities remain between countries. For instance, in India, the incidence of NDs decreased from 30,990.78 per 100,000 in 1990 to 10,620.96 per 100,000 in 2021, representing a reduction of approximately 3.46 times. A similar trend is observed in China, where the incidence rate dropped from 11,654.93 per 100,000 in 1990 to 3,408.88 per 100,000 in 2021, a decrease of 3.73 times (Supplementary Table S2). In terms of mortality rates (Supplementary Table S4), substantial variations exist among countries between 1990 and 2021. In Bolivia, for example, the mortality rate fell significantly from 40.35 per 100,000 to 10.27 per 100,000. Countries like China, India, and Nigeria also reported notable declines in mortality. While Mexico and the Philippines experienced reductions in mortality, these changes were less pronounced. Conversely, in a few nations, including the United States and Zimbabwe, mortality rates have increased. These trends illustrate both the progress made and the ongoing challenges in public health and disease prevention across different regions.

**Figure 2:**
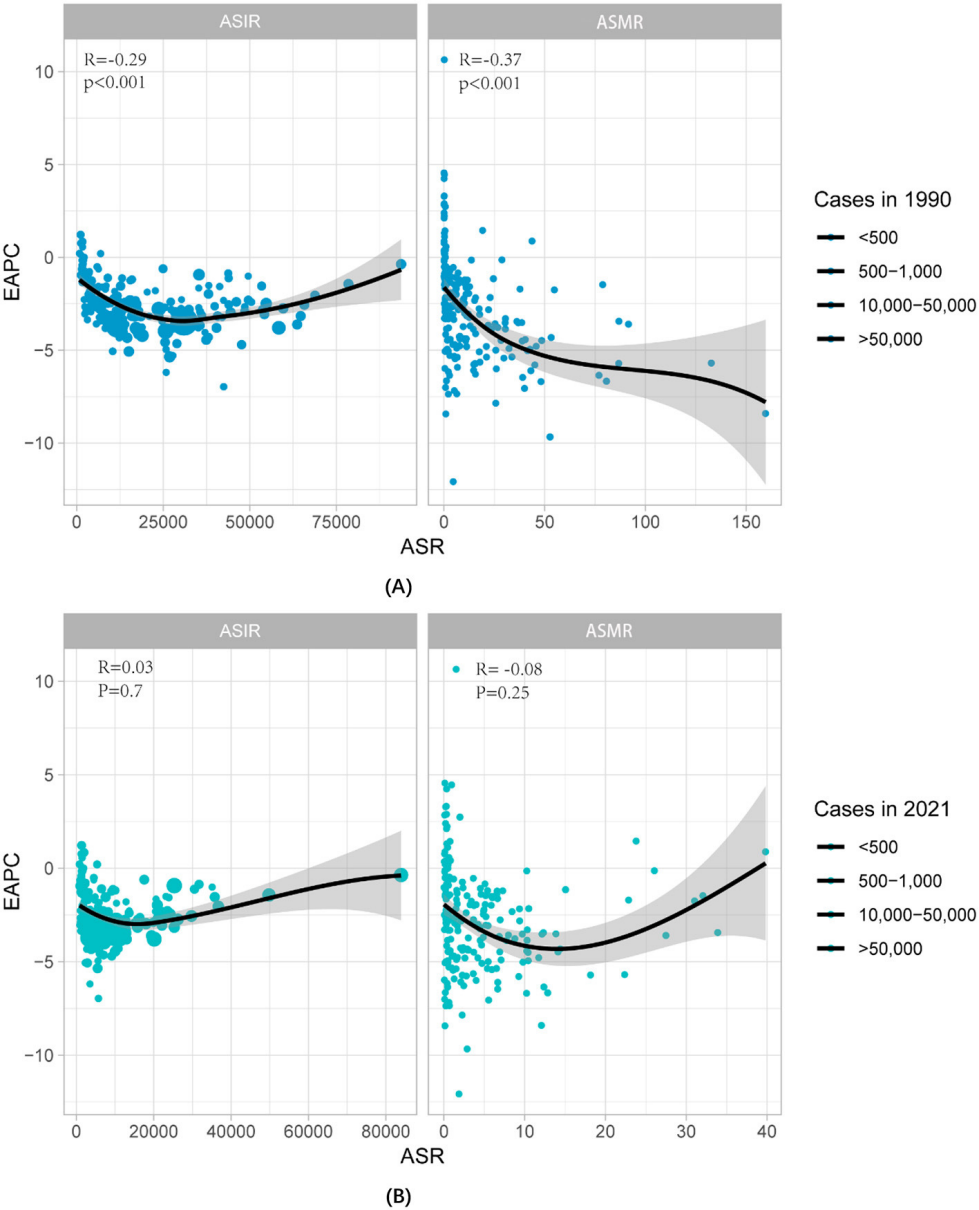
The correlation between EAPC with NDs from 1990-2021 and ASMR and ASIR. **(A)** Data for 1990, and **(B)** data for 2021. The size of the circles is directly proportional to the number of cases of nutritional deficiency. ASMR, Age-standardized mortality rate; ASIR, Age-standardized incidence rate.

From the significant negative correlation between ASIR, ASMR, and EAPC in 1990 (R = −0.29, *P* < 0.001) (R = −0.37, *P* < 0.01) to 2021, the relationship between ASIR and EAPC has become blurred (R = 0.03, *P* = 0.07), and ASMR shows a slight negative trend (R = −0.08, *P* = 0.25). The changes indicate that prevention and treatment of nutritional deficiencies are crucial (Figure 2).

### 3.2 Regional structure

**Figure 3:**
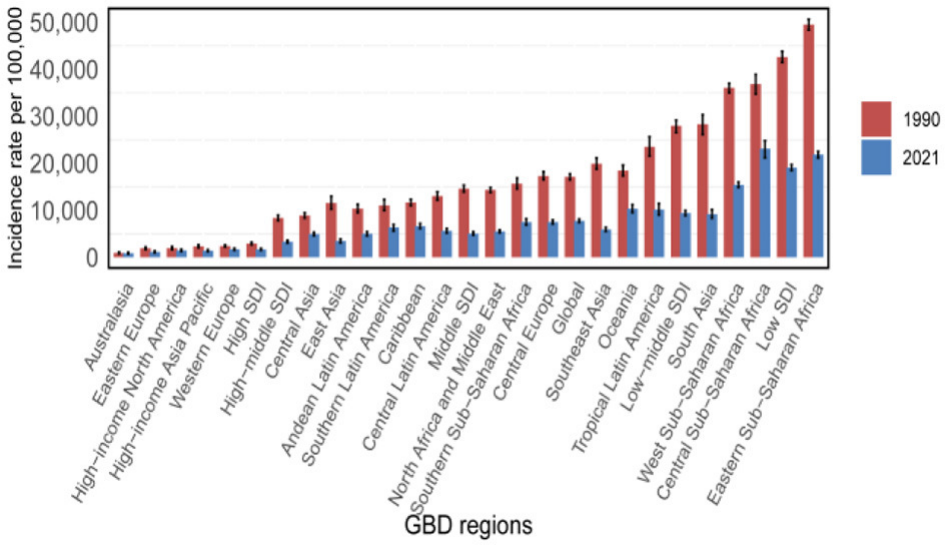
Incidence rate per 100,000 populations in 1990 and 2021.

In 2021, the global burden of nutritional deficiencies revealed significant regional disparities based on SDI (Table 1, Figure 3, Supplementary Figures S2 A–C). In areas with low SDI, ASIR was 19,047.59 cases per 100,000 populations (95% UI: 18,448.14–19,697.06), with ASPR of 44,208.88 cases per 100,000 populations (95% UI: 43,375.1–45,081.24). ASMR in these regions was 8.73 per 100,000 populations (95% UI: 7.44–10.02), and the DALYs amounted to 1,319.28 (95% UI: 1,002.38–1,722.91). In contrast, high SDI regions reported significantly lower figures, with ASIR of 1,671.71 cases per 100,000 populations (95% UI: 1,484.68–1,885.64), ASPR of 6,458.84 cases per 100,000 populations (95% UI: 6,173.06–6,781.27), ASMR of 0.95 per 100,000 populations (95% UI: 0.8–1.03), and a DALYS of 118.26 cases per 100,000 populations (95% UI: 83.05–165.11).

According to our data, South Asia has the greatest frequency of NDs in the world. ASPR is highest in central sub-Saharan Africa, at 44,824.1 per 100,000 people (95% UI: 42,768.74–46,890.42), followed by South Asia at 40,854.69 per 100,000 people (95% UI: 39,854.44–41,943.76) (Table 1, Figure 2A). Sub-Saharan Africa likewise has a high ASPR, with 39,924.67 per 100,000 persons (95% UI: 38,899.57–40,949.31), placing third among the examined areas. The central area of Sub-Saharan Africa bears the greatest burden of worldwide NDs prevalence. This might be related to genetic predisposition, greater prevalence of hypertension and diabetes, inadequate access to appropriate health care, and a lack of understanding of stroke risk factors. The high ASPR in South Asia might be attributed to food choices, excessive salt consumption, and fast urbanization.

Our findings indicate that the incidence rate of NDs in the Sahara area warrants special attention. According to the research findings, Eastern Sub-Saharan Africa and Central Sub-Saharan Africa have some of the highest ASIRs in the world. The reported ASIR for Eastern Sub-Saharan Africa is 49,502.75 per 100,000 people (95% UI: 48,371.01–50,624.14), whereas the ASIR for Central Sub–Saharan Africa is 23,067.04 per 100,000 people (95% UI: 21,152.87–24,805.25). These ratios rank first and second, respectively. In contrast, Australia has the lowest ASIR, with 845.61 per 100,000 persons (95% UI: 741.33–996.47) (Table 1, Figure 2). Furthermore, the temporal trend from 1990 to 2021 reveals distinct trends throughout the Sahara area. The greatest substantial decline in ASIR occurs in sub-Saharan Africa, with an EAPC of −2.81 (−3.01 to 2.62). On the contrary, the Australian area is trending higher, with an EAPC of 0.03 (−0.18–0.24). These opposing patterns emphasize the complexity and variety of NDs incidence rates in different locations of the Sahara, emphasizing the need for focused intervention and more study to address these disparities.

The ASDR for NDs is much greater in eastern, central, and western Sub-Saharan Africa. Between 1990 and 2021, the ASDR of NDs declined the highest in East Asia [EAPC-8.07 (95% UI: −10.13–5.97)] (Table 1). Sub-Saharan Africa continues to have the greatest age-standardized disability adjusted life expectancy, with 4,027.09 per 100,000 people (95% UI: 3,326.07–5,033.9). From 1990 to 2021, South Asia had the lowest age-standardized DALY incidence of NDs [EAPC-6.17 (95% UI: −7.52–4.8)].

While the statistics show obvious disparities in nutritional burden and nutritional deficiencies (ND) among locations with varying SDI levels, the underlying reasons are diverse. These determinants include disparities in health-care access, socioeconomic position, public health policy, food habits, and environmental conditions. Nutritional deficiencies is more prevalent in low-SDI regions, owing mostly to a lack of health resources, poverty, food insecurity, and ineffective public health policy. Nutritional deficiencies is less prevalent in high SDI regions because to well-established health systems, better incomes, healthy diets, and effective public health treatments. Nutritional deficiencies may be more prevalent in South Asia and Sub-Saharan Africa due to poor eating habits (e.g., excessive salt, processed foods), fast urbanization, hereditary factors, and insufficient health-care resources. Nutritional deficiencies in these communities might be exacerbated by food shortages and inadequate sanitation. Economic growth, improved public health regulations, healthier diets, and lifestyle changes have all contributed to improved nutritional status in East Asia, resulting in a decrease in the burden of nutritional deficiencies.

In addition, Potential confounding factors can potentially influence the outcome. Low SDI regions, like as Sub-Saharan Africa and South Asia, frequently compound the burden of nutritional deficiencies as a result of food supply disruptions, infrastructure loss, and a lack of health resources caused by political instability and violence. These places are also susceptible to natural disasters (such as droughts and floods), which affect agricultural productivity and produce food shortages, increasing hunger. Weak health systems and a lack of health resources in low-SDI regions result in many nutritional deficiencies cases that are not treated in a timely way, increasing the disease burden. High-salt, high-fat diets in South Asia and Sub-Saharan Africa, combined with fast urbanization, have resulted in an increase in metabolic disorders (such as hypertension and diabetes) associated with nutritional deficiencies.

### 3.3 Temporal connection point analysis

**Figure 4:**
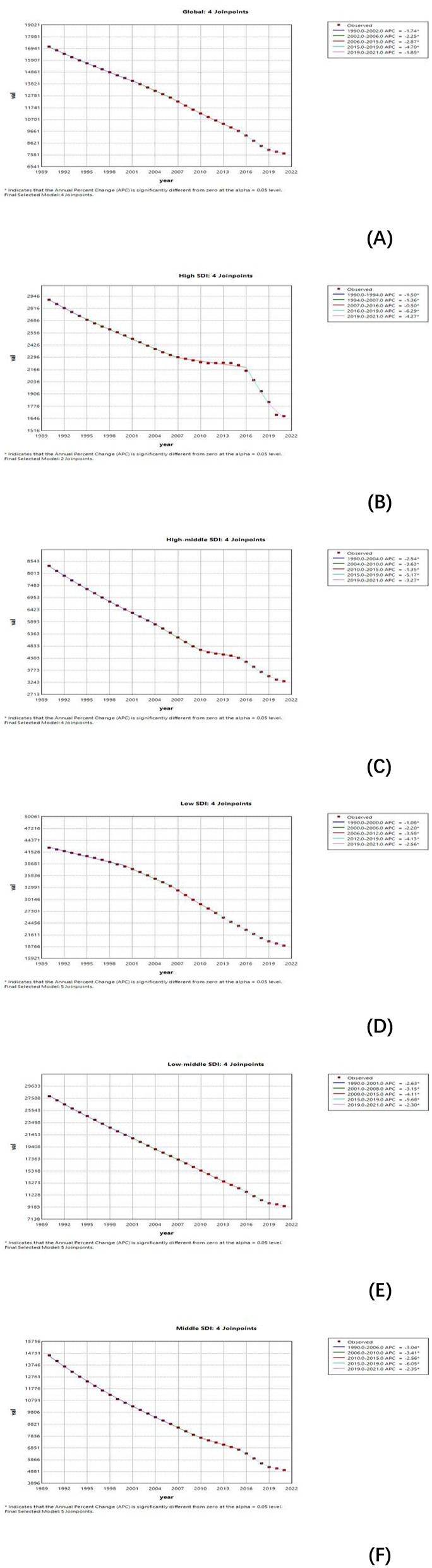
Joinpoint regression analysis of age-standardized Incidence rate (ASIR) for NDs in global and different SDI from 1990 to 2021. **(A–F)** Shows the results of Joinpoint regression analysis of nutritional deficiencies incidence globally and SDI region, covering 1990–2021. Each graph shows the APC for each time period, indicating statistically significant results (*P* < 0.05).

From 1990 to 2021, the global and those of the five SDI regions of ASIR generally showed a downward trend. Notably, high-SDI regions experienced a marked decline between 2016 and 2019 (Figure 4). Similarly, the ASPR exhibited an overall decrease during this period (Supplementary Figure S3A). The ASMR showed more variation across regions. While there was a global decline, ASMR experienced a temporary increase between 2008 and 2011 before resuming its downward trajectory. Regional trends revealed upward fluctuations in ASMR for high-SDI, medium-low SDI, and low-SDI regions during specific periods, followed by subsequent declines (Supplementary Figure S3B). Disability-Adjusted Life Years (DALYs) also displayed diverse fluctuation patterns across regions, reflecting varying disease burdens. Trends in the Age-Standardized Disability Rate (ASDR) underscore the need for sustained, targeted interventions, particularly in low-SDI and low-to-medium SDI regions, to mitigate the persistent burden of non-communicable diseases (NDs) (Supplementary Figure S3C).

### 3.4 Age and gender patterns

**Figure 5:**
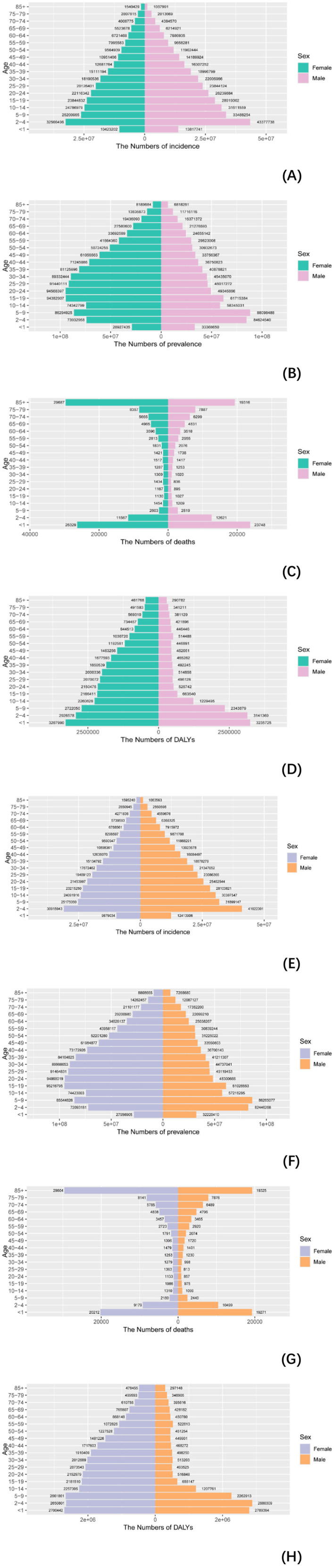
Comparison of incidence, prevalence, deaths, and DALYS in males and females. **(A–D)** Comparison of incidence, prevalence, mortality, and DALYS numbers in males and females in 1990; **(E–H)** comparison of incidence, prevalence, deaths, and DALYs between men and women in 2021.3.5. Hierarchical cluster analysis.

In 2021, significant disparities in the global burden of NDs emerged across genders, regions, and age groups. Data presented (Supplementary Tables S6–S12, Figure 5) reveal a decline in the incidence rate for both men and women since 1990, with average decreases of 2.76% for men and 2.22% for women. Notably, in high-SDI regions, individuals experienced a slow reduction, with incidence rates falling by 1.26% for men and 1.55% for women. In Low-middle SDI regions, the decline was steeper, showing a decrease of 3.76% for men and 3.06% for women. This indicates that different levels of development influence the trends in nutritional deficiency burdens across genders and age groups. Regarding prevalence, the average annual decline rates were 0.37% for men and 0.15% for women. The mortality rates for individuals showed a more significant decline, with average annual decreases of 0.71% for men and 0.74% for women. In terms of DALYs, the reductions were 0.59% for men and 0.47% for women. Overall, these findings highlight that while the global burden of NDs has diminished, substantial differences remain across genders, age groups, and regions, with a more rapid decline observed in low-SDI areas compared to high-SDI areas.

### 3.5 Hierarchical cluster analysis

**Figure 6:**
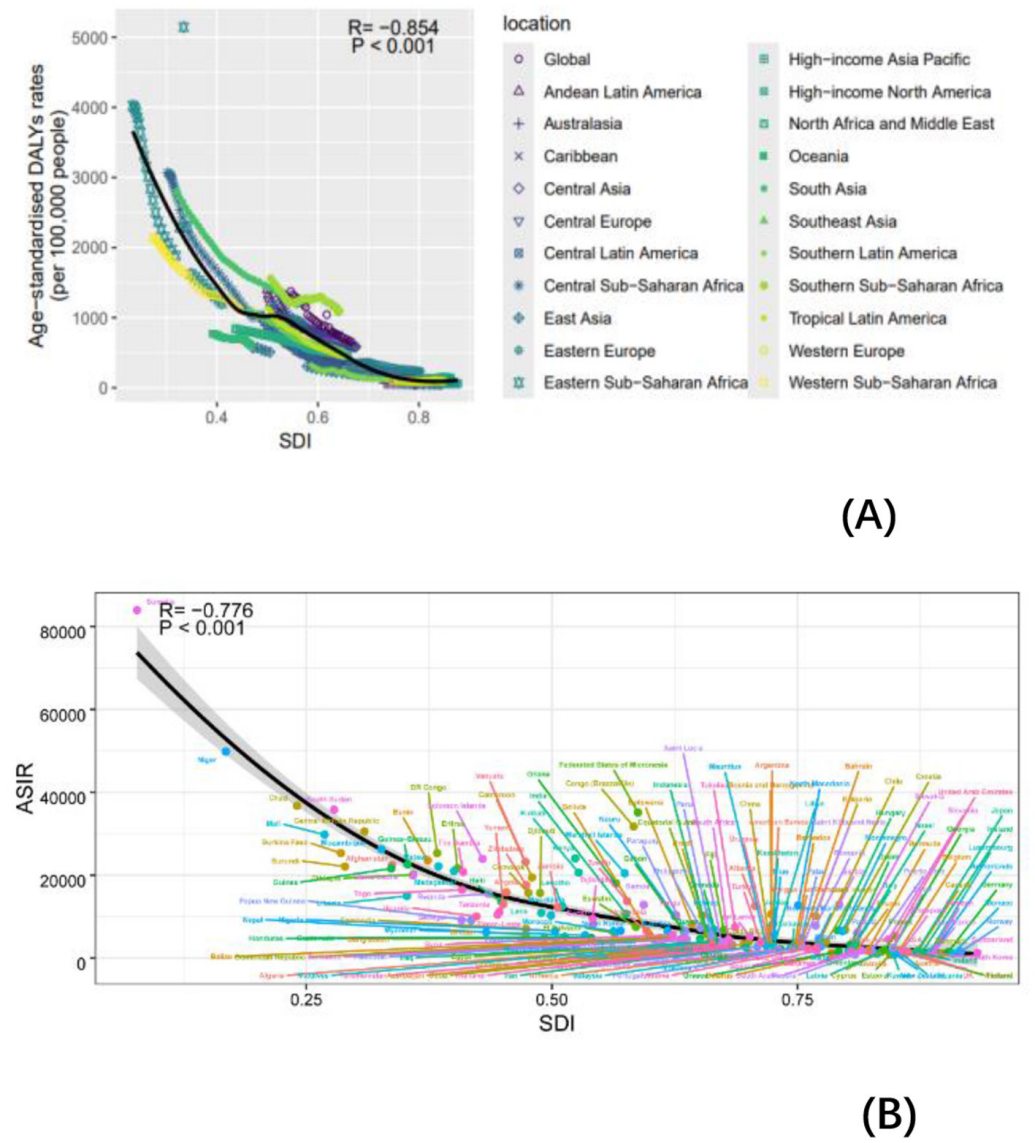
Incidence rate per 100,000 populations in 1990 and 2021. ASIR for NDs of 21 regions and 204 countries and territories by SDI. **(A)** ASIR for NDs of 21 regions from 1990 to 2021 according to the SDI. **(B)** ASIR for NDs of 204 countries and territories in 2021 according to the SDI.

Our analysis of age-standardized rates (ASR) across varying socio-demographic index (SDI) levels included data from 204 countries and regions. Figure 4 illustrates the relationship between observed ASR at regional and national levels and the expected values based on SDI for each region. In 2021, the incidence, mortality, and disability-adjusted life year (DALY) rates for non-communicable diseases (NDs) were inversely correlated with SDI (Supplementary Figure 4B, Figure 6). Similarly, across 21 regions, the prevalence, incidence, mortality, and DALY rates for NDs showed a consistent negative correlation with SDI (Supplementary Figure 4A, Figure 6). Higher SDI levels were associated with lower DALYs, as well as reduced age-standardized incidence rates (ASIR), prevalence rates (ASPR), and mortality rates (ASMR). Throughout the study period, regions with high SDI, such as Western Europe and high-income Asia Pacific, exhibited significantly lower ASR rates, while low-SDI regions, including Sub-Saharan Africa and South Asia, had substantially higher ASR rates. These findings underscore the urgent need for stronger public health policies and targeted interventions in low-SDI regions.

## 4 Discussion

### 4.1 Trends in the global burden of NDs

This is the detailed report on the incidence, prevalence, deaths and DALYs of NDs in the period 1990–2021 globally and across specific countries, based on the newly update data from the GBD 2021. Globally, from 1990 to 2021, we show that the absolute incidence was reduced by 39%, deaths by 61% and DALYs by 38%, but with prevalence increased by 4.5%. All age-adjusted metrics showed increase, especially prevalence. Across GBD regions, in 2021, the highest incidence, prevalence, deaths were found in South Asia and DALYs in Eastern Sub-Saharan Africa. Over the past 30 years, incidence and DALYs showed that the decline rate for males is higher than that for females, both in terms of absolute decline rate and EAPC. However, the opposite trend is observed for prevalence and mortality rates. For incidence, prevalence, and DALYs, the overall rates decrease with increasing age. However, mortality rates are higher at both ends of the age spectrum—among the young and the elderly—exhibiting a dumbbell-shaped pattern. Furthermore, studies have shown that childhood nutrition deficiency is associated with a variety of diseases, including diabetes risk in adulthood (20), hypertension (21), adult diabetes (22), and depressive symptoms (23). This indicates that children are a high-risk age group for NDs, warranting special attention.

From 1990 to 2021, the global absolute prevalence of NDs decreased by 4.5%, while the global age-standardized prevalence of NDs increased by 26%. Several factors may explain the significant discrepancies between the adjusted GBD prevalence estimates and absolute changes. These differences are largely attributed to incomplete reporting and poor data quality (24). Carl Lachat noted similar issues in 17 literature reviews conducted prior to the fifth revision of the Nordic Nutrition Recommendations (25), highlighting that the lack of methodological details was a common problem (24).

The findings show that the incidence, prevalence, mortality, and DALYs of dietary deficiencies differ among regions. This variance may be linked to a number of things. First, improved dietary patterns and increased nutritional treatments, notably in China and India, have led to a reduction in ND incidence and mortality. Furthermore, government measures such as food fortification and nutritional supplementation programs, particularly in disadvantaged regions, have greatly reduced the prevalence of critical nutrient shortages. Second, advancements in healthcare infrastructure have made early detection and treatment more accessible, resulting in fewer fatalities due to nutritional deficiencies. Although mortality rates have decreased in certain countries, such as Mexico and the Philippines, the improvements have been minimal, presumably due to inequalities in resource allocation and policy execution. Rising mortality rates in nations such as the United States and Zimbabwe might be attributed to poverty, unequal access to healthcare, and ongoing dietary concerns. Overall, changes in nutritional deficiencies reflect the interaction of food habits, healthcare systems, governmental measures, and socioeconomic inequities.

### 4.2 Regional differences: burden of NDs

In 2021, the incidence, prevalence, deaths, DALYs of NDs cases was highest in low- and middle-SDI regions, age-adjusted rankings of all indicators are similar to the actual rankings, indicating that the burden of NDs remains significant in these areas. The burden of malnutrition in Nepal demonstrates a rapid increase in the frequency of high BMI and the ongoing presence of malnutrition suggests a dual burden of malnutrition in Nepal (26). Meanwhile, the absolute change in four indicators in low-middle SDI regions have most significant change, demonstrating that measures implemented by these countries have been notably effective. To further reduce this burden, continued strengthening of public health interventions, particularly in nutrition, basic healthcare, and disease prevention, is crucial.

The high actual prevalence, incidence, mortality, and DALYs of nutritional deficiencies in 2021 were mainly observed in China and India. Wei et al. used the CHARLS database to investigate the frequency and predictors of malnutrition among China's elderly (27). Furthermore, regional research on malnutrition in India is more refined, as seen in specific regions such as the Great Andamanese tribe (28), West Bengal (29) and South India (30), where malnutrition research has been conducted. However, the age-standardized incidence, prevalence, mortality, and DALYs were highest in Somalia. The regional differences can be explained by two main hypotheses. First, due to the large population base in China and India (31, 32), even though the incidence rate per person is relatively low, the total number of cases remains high. Another explanation could be that Somalia and Sierra Leone have a younger population, and factors such as prolonged conflict, poverty, and a weak healthcare system (33) have led to higher nutritional deficiencies risks for these younger groups, which in turn result in higher mortality, incidence, and DALYs. International organizations and NGOs are encouraged to increase support with emergency medical aid, food security, and healthcare, especially for child and maternal nutrition. For example, the Indian government established the Integrated Child Development Services (ICDS) in 1975, the National Rural Health Mission (NRHM) in 2005, and the National Noncommunicable Disease Surveillance Framework and Action Plan in 2014 (34). These programs attempt to provide the most basic nutritional interventions while also protecting the most disadvantaged populations. Furthermore, the Global Alliance for Nutrition Improvement has conducted programs to boost dietary iron intake in 14 countries, including Vietnam's fish sauce iron fortification program and South Africa's wheat and corn meal iron fortification project (35).

Although the GBD database collects macro health data from 204 nations and 21 regions. However, it lacks additional information on neighborhoods and households. To improve the credibility of future research findings, we might merge data from the China Health and Retirement Longitudinal Survey (CHARLS) (27) and the National Health and Nutrition Examination Survey (NHANES), focusing on more specific data at the household or community level.

### 4.3 Differences among age groups: burden characteristics of NDs

The burden of nutritional deficiencies varies greatly among different age groups. In 2021, the greatest incidence rate will be 94,031,184 children aged 0–4 years old. Children under 5 years old are the important period of growth and development, thus policy guarantee is urgently needed to reduce nutrition deficit (24). The GBD research in 2015 and 2019 found that countries/regions in sub-Saharan Africa and South Asia suffer more from childhood nutritional deficiencies (36, 37). This might be attributed to limited food availability, low household income, and inadequate child care (38). Nutritional deficiencies include trace elements (39) and protein (40).

The recommended dietary protein intake for healthy persons with low physical activity is 0.8 g per kilo-gram of body weight per day. This figure is significantly greater for newborns and toddlers as they develop and absorb protein (41). As a result, we must pay more attention to the problem of dietary deficits in children. An observational study in several countries recommends improving the quality of school meals to address nutritional concerns such as developmental delay in children aged four and above (42). The analysis revealed a dumbbell-shaped age distribution in terms of death rate. This phenomenon serves as a warning that mortality due to nutritional inadequacies are not only common in youngsters, but also a major worry for the elderly.

As a result, a decent diet and proper nutritional supplements are essential for children's healthy growth and the long-term survival of the elderly (43, 44).

### 4.4 Gender differences: comparison of the burden between men and women

From 1990 to 2021, all four indicators showed a downward trend in both men and women, indicating that public health and medical interventions have made some progress in reducing the health burden. The decline of incidence and DALYs in men was greater than that in women, indicating that many high-risk factors affecting men's health, such as smoking (45), alcoholism (46), poor eating habits, etc., have been greatly controlled and improved in the past few decades. The decrease in prevalence and deaths in women was greater than that in men, indicating that the improvement of maternal health, such as maternal care, childbirth care (47), vaccination, etc., may have achieved significant results in reducing female mortality and morbidity.

For the four indicators, incidence, prevalence, deaths and DALYs, the highest regions for men and women in 2021 were low SDI and low-middle SDI, which were unchanged after age-adjusted standardization. This shows that the burden of nutritional deficiencies in both regions is severe for both men and women, and the government should take active measures. Incidence, prevalence and DALYs, except for deaths, the highest figures for men and women in 2021 are all South Asian, but after age-adjusted standardization, they are Central and Eastern Sub-Saharan Africa (48, 49), respectively, and the possible reasons for this phenomenon are as mentioned above. In terms of absolute change, South and East Asia are leading the way, indicating that nutritional deficiencies has improved dramatically in these contiguous regions.

In low-income regions such as Southeast Asia, despite declining incidence rates, the need for public health intervention remains high due to scarce resources. These areas need to strengthen infrastructure and increase access to medical resources to effectively reduce the burden of NDs. In particular, the EAPC in Southeast Asia shows that ASIR has decreased at an average annual rate of −3.74%, which shows that public health intervention in this region has significant effects and should serve as a reference template for other low-SDI regions.

## 5 Limitation

The study has significant drawbacks. First, because the study depends primarily on data at the regional and national levels for analysis, the ecological fallacy may emerge due to a lack of meaningful changes at a local geographic level or within a specific subgroup. Furthermore, while GBD is a reliable source, some variations in the completeness and accuracy of GBD data are unavoidable because to disparities in medical standards and economic situations among countries and regions.

## 6 Conclusions

Although the global burden of NDs has declined overall, age-specific, gender- and region-specific disparities remain significant. Future policies should pay more attention to low-SDI areas, children, male groups, and areas with scarce medical resources to achieve a more balanced distribution of health resources and reduce the burden. In the future, it will also be important for clinicians or nutritionists to provide an affordable nutritional treatment or a predictive and effective nutritional status assessment program for high-risk groups.

## Data Availability

The original contributions presented in the study are included in the article/Supplementary material, further inquiries can be directed to the corresponding authors.
