# Supplementary material for: Analysis of study Global Burden of Disease in 2021: global, regional, and national burden of nutritional deficiency from 1990 to 2021
Source: Front Nutr. 2025 Jan 15;11:1540485. doi: 10.3389/fnut.2024.1540485 (PMC11774684; doi:10.3389/fnut.2024.1540485)

Supplementary Material

# **1 Supplementary Tables and Figures**

## 1.1 Supplementary Tables

## Table S1: The global Incidence, prevalence, deaths and DALYs of Nutritional Deficiencies in cases and age-standardized rates for both sexes combined from 1990 to 2021 by year.

| Year | Incidence | | Prevalence | | deaths | | DALYs | |
| --- | --- | --- | --- | --- | --- | --- | --- | --- |
|  | All-ages cases (both sexes) | Age-standardized rates per 100,000 people | All-ages cases (both sexes) | Age-standardized rates per 100,000 people | All-ages cases (both sexes) | Age-standardized rates per 100,000 people | All-ages cases (both sexes) | Age-standardized rates per 100,000 people |
| 1990 | 964881696 (928560815,1001236465) | 17112.55 (16470.31,17731.43) | 1765110763 (1735880718,1794376240) | 32217.95 (31693.55,32740.92) | 570119 (485777,693881) | 10.9 (9.44,12.97) | 78674224 (64859007,97375904) | 1367.15 (1126.3,1708.49) |
| 1991 | 958455985 (924715198,993665124) | 16787.91 (16184.76,17399.89) | 1770858334 (1743452382,1799202525) | 31880.19 (31381.24,32405.75) | 561614 (480700,682515) | 10.67 (9.26,12.63) | 77999471 (64444773,96515218) | 1345.78 (1114.41,1675.79) |
| 1992 | 951563955 (917370886,989315049) | 16475.57 (15869.46,17110.85) | 1775596630 (1748442760,1804745731) | 31552.29 (31067.2,32078.17) | 552738 (475662,672970) | 10.45 (9.1,12.45) | 77287329 (63810013,95588468) | 1326.03 (1097.14,1650.36) |
| 1993 | 944672154 (911038981,982170998) | 16183.13 (15604.43,16804.74) | 1779945726 (1753065428,1809795607) | 31245.02 (30769.11,31760.9) | 528405 (456853,634486) | 9.92 (8.7,11.73) | 76349532 (62948987,94397626) | 1303.43 (1076.47,1620.5) |
| 1994 | 937542906 (904545162,974821602) | 15907.34 (15354.07,16524.71) | 1783676531 (1757620697,1813149162) | 30957.95 (30518.42,31456.01) | 616151 (497919,797431) | 11.64 (9.51,15.11) | 75373879 (61832725,93905901) | 1281.2 (1053.5,1599.85) |
| 1995 | 930744557 (898557650,966377826) | 15650.26 (15117.72,16245.26) | 1787898117 (1761465786,1817428623) | 30696.21 (30253.96,31185.98) | 564152 (466072,704612) | 10.51 (8.75,13.1) | 81091993 (66202662,101509720) | 1372.72 (1120.15,1718.87) |
| 1996 | 923771718 (892429262,958355444) | 15395.2 (14866.05,15975.39) | 1791796590 (1765746006,1821943253) | 30434.17 (29989.76,30924.94) | 547192 (453455,690184) | 10.14 (8.46,12.74) | 78430111 (64709812,98511843) | 1321.84 (1090.21,1659.15) |
| 1997 | 915801107 (885571225,950987506) | 15128.23 (14624.36,15693.15) | 1793928987 (1767312866,1824834727) | 30146.03 (29719.38,30636.56) | 529138 (436816,667110) | 9.73 (8.11,12.18) | 77347204 (63766229,97109992) | 1297.4 (1070.36,1626.82) |
| 1998 | 907255044 (876890159,943085766) | 14858.1 (14354.64,15424.36) | 1795636825 (1769474225,1826849644) | 29856.8 (29429.95,30347.68) | 513945 (424674,650314) | 9.37 (7.81,11.79) | 75998340 (62298314,95682153) | 1268.46 (1041.74,1592.66) |
| 1999 | 898858297 (868480305,935353120) | 14595.36 (14097.77,15178.96) | 1798399581 (1772398244,1829890369) | 29589.49 (29158.8,30079.01) | 385511 (342890,445167) | 6.84 (6.11,7.81) | 74673003 (61290244,94117285) | 1239.34 (1019.67,1558.32) |
| 2000 | 891427153 (860677265,929382439) | 14350.19 (13850.18,14951.02) | 1803520704 (1778068646,1835209205) | 29359.98 (28938.94,29862.02) | 496189 (410466,633125) | 8.97 (7.48,11.35) | 73637327 (60242627,93089874) | 1214.14 (996.83,1531.28) |
| 2001 | 883555172 (852665929,921477730) | 14097.5 (13600.41,14680.48) | 1809901289 (1784014247,1840550750) | 29145.56 (28723.42,29628.65) | 365749 (325670,421061) | 6.41 (5.76,7.31) | 72330343 (58875243,92233086) | 1183.8 (966.56,1505.4) |
| 2002 | 874171850 (842645732,911199424) | 13817.95 (13320.71,14398.14) | 1816121312 (1789172279,1847082992) | 28918.54 (28490.77,29396.03) | 351561 (314776,400232) | 6.08 (5.45,6.88) | 70987510 (57865193,90851881) | 1152.13 (941.53,1473.27) |
| 2003 | 864041899 (832743108,900083359) | 13525.88 (13039.28,14100.58) | 1822175610 (1794496155,1853032942) | 28683.08 (28252.56,29161.47) | 340129 (305025,386465) | 5.79 (5.24,6.54) | 64832370 (52601792,83409843) | 1041.27 (846.5,1332.94) |
| 2004 | 853505176 (821436512,890610045) | 13228.57 (12739.71,13809.79) | 1827835354 (1799191359,1858942332) | 28437.6 (28003.42,28912.38) | 321225 (288809,360772) | 5.3 (4.8,5.92) | 63470011 (51183950,81876635) | 1009.02 (815.4,1296.83) |
| 2005 | 843258865 (810242704,880980963) | 12937.65 (12433.41,13520.16) | 1833185826 (1804391034,1865142690) | 28185.39 (27753.72,28659.08) | 330060 (297177,372398) | 5.53 (5.01,6.19) | 62504741 (50360768,80330537) | 983 (793.76,1259.67) |
| 2006 | 832033872 (798973642,869826203) | 12631.54 (12137.26,13210.84) | 1836123322 (1807284454,1868210550) | 27891.65 (27460.57,28370.58) | 311414 (281334,349251) | 5.06 (4.6,5.65) | 61661247 (49387862,79557042) | 958.42 (769.52,1234.58) |
| 2007 | 818425471 (785738009,856664345) | 12288.89 (11795.41,12859.64) | 1835409278 (1806119374,1868789689) | 27537.69 (27113,28028.63) | 330764 (301093,364765) | 5.28 (4.8,5.79) | 60699758 (48445232,78363040) | 931.86 (745.16,1201.17) |
| 2008 | 803450790 (770398397,840329747) | 11927.19 (11438,12470.39) | 1833347523 (1804242791,1868075011) | 27161.1 (26741.08,27650.11) | 490431 (455286,526643) | 7.56 (7.01,8.11) | 59742238 (47481299,76854190) | 905.66 (721.48,1163.32) |
| 2009 | 788293779 (755492489,825023291) | 11568.36 (11086.26,12101.12) | 1832323531 (1803491074,1867359246) | 26804.16 (26397.02,27306.14) | 269931 (243379,298610) | 3.99 (3.61,4.42) | 58757296 (46440840,75605474) | 879.81 (697.06,1130.48) |
| 2010 | 773876033 (741211553,811121639) | 11236.35 (10755.52,11768.05) | 1834144695 (1805163277,1870487098) | 26512.83 (26105.95,27029.76) | 325556 (296454,356105) | 5.03 (4.58,5.49) | 59762510 (47623249,76379148) | 885.87 (707.61,1129.87) |
| 2011 | 760155720 (728034426,797907983) | 10929.11 (10458.11,11463.05) | 1838664158 (1809323865,1876695795) | 26281.96 (25870.14,26820.71) | 281763 (255007,310730) | 4.31 (3.91,4.74) | 70805078 (58765659,87824953) | 1042.85 (868.26,1290.2) |
| 2012 | 746437990 (714223004,784977601) | 10624.96 (10158.15,11163.01) | 1844027408 (1814329176,1881652422) | 26061.13 (25652.28,26589.48) | 275132 (248763,304718) | 4.14 (3.75,4.58) | 59171610 (47032999,75784685) | 859.95 (685.8,1098.69) |
| 2013 | 732526809 (699985970,770836151) | 10320.28 (9863.48,10854.72) | 1849530590 (1819135250,1887520970) | 25839.93 (25426.23,26364.26) | 265664 (238944,295117) | 3.86 (3.47,4.3) | 56087328 (43701529,72571515) | 804.88 (630.33,1039.12) |
| 2014 | 717919908 (685137691,756189411) | 10011.97 (9560.31,10539.08) | 1854155194 (1823300350,1892304160) | 25611.95 (25191.92,26123.54) | 259387 (234373,287763) | 3.71 (3.34,4.12) | 55411399 (42926403,72250335) | 786.46 (613.33,1023.58) |
| 2015 | 702325961 (669341249,741178102) | 9694.72 (9244.41,10219.65) | 1857312688 (1825361488,1895906391) | 25367.18 (24942.89,25872.36) | 251771 (227797,280100) | 3.55 (3.2,3.96) | 54805600 (42301745,71824556) | 769.25 (595.35,1006.72) |
| 2016 | 680864836 (648779586,718392414) | 9302.21 (8870.11,9806.65) | 1855760458 (1824304181,1893794688) | 25063.77 (24643.84,25571.15) | 578013 (473364,722754) | 10.84 (8.98,13.55) | 54076992 (41601622,70961970) | 750.63 (578.8,983.07) |
| 2017 | 652923885 (622943977,688043865) | 8833.92 (8430.86,9305.07) | 1849157502 (1817870411,1885954520) | 24709.16 (24302.55,25193.76) | 222274 (199731,247630) | 3.03 (2.69,3.4) | 52984276 (40387411,69813551) | 728.5 (557.36,957.51) |
| 2018 | 624892951 (597660751,657705682) | 8381.89 (8006.84,8819.2) | 1841854276 (1809785087,1878095019) | 24371.29 (23955.45,24838.86) | 540833 (464378,653821) | 10.19 (8.9,12.07) | 51785775 (39158740,68258899) | 706.91 (536.95,931.34) |
| 2019 | 603357453 (578258542,634258616) | 8034.02 (7684.13,8445.76) | 1838645642 (1806285696,1874078044) | 24112.8 (23700.89,24566.55) | 243871 (219080,270465) | 3.4 (3.04,3.79) | 50724460 (37942261,67037817) | 688.43 (518.72,908.78) |
| 2020 | 594974608 (570420155,623406422) | 7874.55 (7542.29,8262.33) | 1841927844 (1808527525,1878199742) | 23968.89 (23550.15,24429.35) | 478506 (397610,611889) | 8.57 (7.16,10.94) | 49733301 (36685597,65835832) | 671.17 (500.63,886.6) |
| 2021 | 586129387 (562138520,614585958) | 7725.1 (7404.01,8109.01) | 1845246558 (1811946448,1882683059) | 23858.99 (23445.77,24320.82) | 231465 (208216,256251) | 3.18 (2.84,3.55) | 48919261 (35986110,64985437) | 657.62 (489.93,869.58) |
| Estimated Annual Percentage Change between 1990 and 2021 | -0.39 (-0.42,-0.36) | -0.55 (-0.57,-0.53) | 0.05 (0.03,0.07) | -0.26 (-0.27,-0.25) | -0.61 (-0.68,-0.53) | -0.72 (-0.76,-0.67) | -0.38 (-0.48,-0.29) | -0.52 (-0.59,-0.46) |

## Table S2: Incidence of Nutritional Deficiencies in cases and age-standardized rates for both sexes combined in 1990 and 2021, with percentage change between 1990 and 2021 by GBD 204 nations.

| Incidence | | | | | |
| --- | --- | --- | --- | --- | --- |
| nations | **1990** | | **2021** | | **1990-2021 EAPC** |
|  | **All-ages cases(both sexs)** | **Age-standardized rates per 100,000 people** | **All-ages cases(both sexs)** | **Age-standardized rates per 100,000 people** |  |
|  | **n (95% UI)** | **n (95% UI)** | **n (95% UI)** | **n (95% UI)** | **n (95% UI)** |
| Slovenia | 173352 (151768-197744) | 9022.92 (7931.63-10264.66) | 90163 (79735-101876) | 4507.64 (3914.94-5123.04) | -2.31 (-2.41--2.22) |
| North Macedonia | 543183 (490130-603643) | 27320.28 (24692.92-30312.41) | 267024 (236509-300857) | 12663.59 (11219.94-14413.87) | -2.82 (-2.97--2.67) |
| Australia | 144280 (121417-174039) | 829.14 (699.27-1000.29) | 252416 (221050-297617) | 830.56 (723.68-991.89) | 0.21 (0.02-0.41) |
| Lithuania | 83925 (74030-94170) | 2324.99 (2049.67-2621.17) | 23769 (20312-27859) | 887.94 (744.57-1055.31) | -3.04 (-3.12--2.96) |
| New Zealand | 45209 (38679-53067) | 1331.5 (1141.53-1563.26) | 48609 (41076-62471) | 910.44 (767.95-1144.72) | -0.63 (-0.91--0.35) |
| Japan | 2358392 (1999574-2780272) | 2107.94 (1785.35-2480.56) | 1819996 (1579817-2132337) | 1450.41 (1256.6-1693.66) | -0.93 (-1.14--0.72) |
| Andorra | 865 (747-1021) | 1775.87 (1541.7-2074.36) | 1052 (764-1474) | 1219.75 (922.6-1656.22) | -0.84 (-1.02--0.66) |
| Taiwan (Province of China) | 915995 (804672-1046565) | 4501.03 (3975.61-5126.78) | 473582 (395548-561352) | 1956.31 (1626.83-2320.74) | -2.13 (-2.47--1.8) |
| Democratic People's Republic of Korea | 2441264 (2131210-2751647) | 11307.98 (9885.31-12795.01) | 1476655 (1270020-1710328) | 6536.66 (5574.4-7592.94) | -3.36 (-3.92--2.8) |
| China | 142370986 (124804697-160715339) | 11654.93 (10317.82-13127.84) | 46003560 (40820797-51637763) | 3408.88 (3026.97-3830.22) | -3.73 (-3.85--3.6) |
| Argentina | 4232766 (3748778-4762751) | 12680.9 (11282.51-14243.45) | 3372244 (2902874-3787885) | 7561.17 (6495.81-8556.88) | -1.54 (-1.79--1.3) |
| Malta | 11060 (9612-12680) | 3220.32 (2787.99-3697.76) | 6452 (5556-7766) | 1490.39 (1274.19-1793.7) | -1.99 (-2.17--1.81) |
| Israel | 437230 (365478-511946) | 8389.48 (7061.53-9800.11) | 347176 (290289-414733) | 3687.01 (3083.02-4378.49) | -2.27 (-2.51--2.03) |
| Fiji | 130284 (113673-146338) | 15517.98 (13688.7-17303.33) | 62700 (53749-71853) | 6734.39 (5797.53-7715.7) | -2.48 (-2.6--2.36) |
| Norway | 66608 (56503-78190) | 1696.5 (1437.11-1986.24) | 92556 (73098-117956) | 1497 (1201.76-1888.54) | 0.56 (0.22-0.91) |
| Canada | 686235 (607474-806491) | 2734.5 (2388.53-3220.6) | 774295 (527351-1049100) | 1869.34 (1353.77-2427.67) | -0.95 (-1.08--0.83) |
| Belarus | 284453 (249577-324485) | 2732.88 (2390.1-3109.03) | 108859 (94216-125418) | 1136.18 (990.77-1301.95) | -2.86 (-2.92--2.8) |
| Papua New Guinea | 693131 (614388-780126) | 15487.05 (13887.33-17124.97) | 1050090 (928693-1194008) | 9065.35 (8097.26-10173.26) | -1.24 (-1.44--1.04) |
| United Kingdom | 1075197 (928508-1228180) | 2084.38 (1775.83-2408.58) | 708459 (592305-842431) | 1081.58 (895.39-1284.6) | -1.27 (-1.58--0.97) |
| Armenia | 56171 (48896-64444) | 1632.78 (1426.39-1866.03) | 31815 (27648-37367) | 1117.55 (965.6-1312.52) | -1.26 (-1.38--1.13) |
| Lao People's Democratic Republic | 1811876 (1702927-1936790) | 38115.12 (35756.25-40619.03) | 843232 (737711-970613) | 10886.8 (9580.99-12395.73) | -4.3 (-4.43--4.16) |
| Maldives | 74234 (67187-81266) | 25850.26 (23425.35-28380.84) | 16205 (14221-18384) | 3517.01 (3105.37-3991.61) | -6.19 (-6.71--5.67) |
| Timor-Leste | 342995 (316861-369901) | 35139.01 (32324.9-38140.61) | 175005 (152771-200491) | 10462.13 (9223.45-11870.23) | -3.79 (-3.96--3.62) |
| Viet Nam | 7156798 (6341033-8085326) | 8265.89 (7345.4-9325.07) | 2573678 (2210006-3012525) | 2874.06 (2460.62-3384.62) | -3.07 (-3.3--2.84) |
| Micronesia (Federated States of) | 53603 (49504-57357) | 49542.99 (45943.2-52735.53) | 36430 (33180-39846) | 35111.52 (32130.6-38368.97) | -1.01 (-1.06--0.96) |
| Turkmenistan | 211375 (186671-244272) | 5245.61 (4667.07-5984.58) | 168109 (146592-189821) | 3241.01 (2845.92-3653.11) | -1.63 (-2.03--1.23) |
| Chile | 1054377 (907987-1220613) | 7546.64 (6536.63-8729.7) | 631946 (541942-738352) | 3573.67 (3049.64-4167.6) | -2.34 (-2.61--2.08) |
| Switzerland | 75795 (62164-92286) | 1130.92 (933.76-1373.36) | 132828 (104483-195403) | 1227.96 (968.72-1757.54) | 0.76 (0.56-0.97) |
| Cambodia | 3732611 (3396133-4075635) | 28950.43 (26413.31-31842.91) | 1250819 (1075867-1446918) | 7041.68 (6097.68-8132.37) | -4.64 (-4.79--4.5) |
| Indonesia | 52424461 (47238674-57576385) | 25695.06 (23340.77-28150.9) | 18917855 (17013054-21453747) | 7120.73 (6419.23-8080.17) | -3.96 (-4.09--3.84) |
| Malaysia | 1180162 (1049994-1334165) | 5916.56 (5295.54-6654.76) | 685042 (607503-775285) | 2282.71 (2013.01-2578.93) | -2.72 (-2.91--2.52) |
| Myanmar | 12471493 (11272315-13806418) | 26845.97 (24271.33-29556.59) | 3074228 (2661416-3522810) | 5486.51 (4754.81-6272.97) | -5.35 (-5.48--5.23) |
| Samoa | 43989 (39132-49398) | 22714.96 (20433.27-25180.52) | 30423 (26132-34945) | 12841.46 (11039.82-14680.92) | -1.8 (-1.83--1.76) |
| Philippines | 12486143 (11134970-13915442) | 17110.52 (15294.48-19062.93) | 8473972 (7292978-9704528) | 7201.99 (6205.97-8202.69) | -2.53 (-2.72--2.33) |
| Thailand | 8721867 (7761208-9725786) | 15056.83 (13588.15-16670.64) | 1760950 (1589206-1947278) | 2847.43 (2548.1-3181.23) | -5.07 (-5.43--4.7) |
| Kiribati | 29909 (27457-32334) | 35251.45 (32299.73-38082.04) | 27472 (24130-31238) | 20594.94 (18230.77-23245.36) | -1.65 (-1.71--1.6) |
| Georgia | 145553 (126729-166785) | 2675.15 (2335.33-3087.6) | 75494 (67398-85984) | 2113.1 (1861.8-2397.37) | -0.78 (-1.29--0.27) |
| Albania | 885169 (798364-980134) | 25380.22 (23093.37-27831.6) | 315809 (282833-354719) | 12539.91 (11167.38-14068.54) | -2.69 (-2.92--2.47) |
| Slovakia | 732652 (643523-824638) | 14096.61 (12381.35-15862.13) | 318237 (281770-360443) | 6029.48 (5388.47-6838.47) | -2.75 (-2.85--2.65) |
| Solomon Islands | 162756 (150343-175124) | 41287.62 (38380.77-44279.08) | 187372 (167802-209241) | 23895.34 (21422.03-26518.04) | -1.57 (-1.67--1.47) |
| Kyrgyzstan | 277275 (239588-321700) | 5911.37 (5166.38-6736.27) | 313234 (269892-359313) | 4504.49 (3915.62-5118.9) | -0.51 (-0.79--0.23) |
| Kazakhstan | 2849439 (2537939-3178802) | 16984.3 (15107.89-18913.08) | 1572077 (1371876-1797252) | 8266.64 (7192.46-9463.2) | -2.32 (-2.43--2.22) |
| Vanuatu | 67160 (61336-73066) | 38143.31 (35130.09-41313.95) | 82173 (71883-92109) | 23178.03 (20438.47-25867.57) | -1.51 (-1.58--1.45) |
| Tajikistan | 776384 (686516-871087) | 13107.05 (11754.63-14495.1) | 998677 (871866-1143982) | 9464.25 (8343.43-10744.55) | -1.12 (-1.49--0.75) |
| Tonga | 22959 (20275-26059) | 20366.07 (18079.57-22788.79) | 12153 (10518-14069) | 10363.25 (9014.72-11812.52) | -1.87 (-1.99--1.75) |
| Antigua and Barbuda | 3755 (3377-4196) | 6260.33 (5640.31-6958.53) | 3371 (3060-3729) | 3623.1 (3290.28-3992.06) | -1.55 (-1.69--1.41) |
| Marshall Islands | 20344 (18450-22171) | 42421.35 (39016.67-45829.49) | 11963 (10517-13534) | 20435.74 (18015.57-22960.17) | -2.31 (-2.36--2.26) |
| Mongolia | 135273 (115659-156909) | 5685.54 (4950.76-6518.93) | 66941 (58371-77211) | 1978.5 (1736.07-2269.38) | -3.58 (-3.7--3.46) |
| Sri Lanka | 3205633 (2870943-3569678) | 17689.51 (15982.02-19548) | 1052732 (936090-1181460) | 5182.72 (4579.93-5814.81) | -4.16 (-4.29--4.03) |
| Bosnia and Herzegovina | 1072833 (967277-1196474) | 23961.41 (21575.12-26618.7) | 332971 (296813-377037) | 10574.34 (9265.24-12085.39) | -3.21 (-3.43--2.99) |
| Bulgaria | 1320583 (1173240-1480064) | 15960.72 (14163.11-18015.86) | 642196 (570341-715763) | 10092.99 (8922.32-11314.93) | -1.19 (-1.38--0.99) |
| Estonia | 36107 (32104-41342) | 2326.26 (2046.54-2663.48) | 12969 (10930-15280) | 980.36 (821.8-1162.6) | -2.66 (-2.83--2.48) |
| Serbia | 2971671 (2697461-3257822) | 31462.23 (28666.85-34472.86) | 1104248 (988202-1233331) | 12917.09 (11551.64-14501.8) | -3.23 (-3.48--2.98) |
| Azerbaijan | 342363 (299476-391008) | 4496.31 (3938.75-5078.47) | 325014 (289105-363254) | 3264.06 (2888.78-3670.19) | -1.27 (-1.76--0.78) |
| Guyana | 112725 (98972-126842) | 13547.24 (11980.78-15206.23) | 35123 (31290-39533) | 4593.41 (4113.92-5150.91) | -3.31 (-3.37--3.25) |
| Haiti | 1878246 (1693108-2063611) | 27155.12 (24458.38-29721.34) | 1788677 (1582763-2026041) | 13635.23 (12090.37-15282.46) | -2.39 (-2.45--2.34) |
| Grenada | 11742 (10192-13407) | 13115.66 (11492.76-14774.77) | 3879 (3419-4374) | 3813.07 (3374.42-4332.77) | -3.87 (-4.07--3.66) |
| Jamaica | 180253 (156288-207537) | 7460.34 (6554.45-8545.04) | 87500 (77047-97655) | 3163.96 (2800.73-3541.34) | -2.72 (-2.79--2.66) |
| Uzbekistan | 1704409 (1476625-1957410) | 7320.35 (6433.19-8321) | 1198367 (1043749-1376013) | 3450.76 (3015.92-3941.1) | -2.52 (-2.58--2.45) |
| Poland | 5955579 (5306729-6707850) | 15906.48 (14157.99-17908.28) | 2184705 (1928919-2460983) | 5851.15 (5105.5-6558.17) | -3.16 (-3.22--3.11) |
| Saint Vincent and the Grenadines | 12522 (10845-14366) | 11141.18 (9840.64-12610.8) | 5124 (4576-5699) | 4525.89 (4018.2-5058.8) | -2.83 (-2.94--2.72) |
| Czechia | 1180583 (1037299-1328063) | 11858.87 (10433.69-13411.64) | 601212 (535223-667988) | 5581.26 (4969.18-6274.69) | -2.07 (-2.28--1.85) |
| Brunei Darussalam | 10176 (8846-11590) | 3618.44 (3170.01-4125.97) | 8374 (7296-9484) | 1938.02 (1699.84-2189.7) | -1.83 (-1.96--1.7) |
| Hungary | 1386463 (1220497-1570928) | 13926.1 (12261.85-15799.96) | 619087 (543895-698213) | 6674.31 (5853.18-7564.85) | -2.39 (-2.5--2.29) |
| Croatia | 627743 (553791-701831) | 13386.48 (11857.19-15056.82) | 270684 (242215-304818) | 6771.46 (5998.09-7768.86) | -2.52 (-2.71--2.33) |
| Ukraine | 1707750 (1488037-1957176) | 3430.51 (2981.4-3955.33) | 840034 (726433-961967) | 2296.65 (1959.82-2678.04) | -0.83 (-1.04--0.63) |
| Latvia | 56610 (49026-64213) | 2176.26 (1881.25-2474.31) | 18889 (16106-21888) | 1027.8 (861.73-1206.93) | -2.27 (-2.42--2.12) |
| Montenegro | 75812 (66915-85752) | 12164.09 (10767.56-13734.59) | 38781 (34616-43441) | 6451.62 (5682.2-7242.35) | -2.62 (-3.01--2.23) |
| Romania | 3782195 (3361002-4221152) | 16638.69 (14792.67-18552.31) | 1415175 (1240174-1608919) | 7765.5 (6755.25-8921.49) | -2.57 (-2.73--2.41) |
| Singapore | 103124 (89280-120101) | 3573.42 (3099.35-4120.56) | 96930 (79299-117889) | 1844.55 (1505.13-2241.79) | -1.98 (-2.05--1.91) |
| Republic of Moldova | 143813 (126864-163736) | 3230.46 (2855.75-3667.21) | 57307 (49603-65902) | 1608.17 (1380.26-1867.07) | -2.24 (-2.43--2.06) |
| Russian Federation | 1745083 (1451060-2070278) | 1247.86 (1030.67-1484.62) | 1109268 (898981-1357447) | 837.81 (663.81-1032.6) | -0.95 (-1.11--0.79) |
| Iceland | 4610 (4011-5359) | 1855.05 (1606.19-2152.68) | 4851 (3968-6817) | 1285.56 (1049.09-1758.01) | -0.8 (-0.94--0.66) |
| Finland | 85331 (73776-100100) | 1887.99 (1617.93-2204.56) | 60845 (49335-73999) | 1120.46 (923.99-1356.08) | -1.19 (-1.36--1.02) |
| France | 1042806 (920057-1192518) | 1670.66 (1470.24-1898.18) | 1639173 (1342103-2377297) | 1940.24 (1598.86-2759.26) | 0.84 (0.65-1.04) |
| Belgium | 198154 (175490-224567) | 2138.65 (1855.47-2442.43) | 248397 (200656-352682) | 1817.32 (1490.15-2502.16) | 0 (-0.18-0.19) |
| Netherlands | 283794 (234530-375097) | 1941.51 (1612.86-2496.06) | 330181 (227820-444400) | 1629.57 (1166.39-2169.91) | -0.14 (-0.4-0.11) |
| Austria | 145843 (124870-172336) | 2064.92 (1749.82-2440.72) | 104421 (80651-148751) | 1238.33 (956.54-1719) | -1.13 (-1.31--0.94) |
| Italy | 2431566 (2175902-2705197) | 4269.22 (3790.66-4808.3) | 1597263 (1378678-1868367) | 2326.37 (2003.92-2646.71) | -1.25 (-1.5--1) |
| Denmark | 73224 (61577-86322) | 1536.77 (1305.55-1778.12) | 76778 (66712-98721) | 1160.41 (998.62-1432.66) | -0.42 (-0.62--0.21) |
| Cyprus | 25797 (22414-29788) | 3454.8 (2986.09-3987.38) | 20914 (18370-23695) | 1490.09 (1312.08-1682.86) | -2 (-2.34--1.66) |
| Peru | 2784405 (2429399-3122169) | 11994.38 (10623.91-13308.55) | 1984616 (1713871-2269983) | 5472.83 (4741.16-6226.42) | -2.98 (-3.22--2.74) |
| Republic of Korea | 1222904 (1076650-1388147) | 2840.58 (2517.91-3225.29) | 621069 (538845-723060) | 1143.91 (992.18-1326.67) | -2.92 (-3.04--2.8) |
| Germany | 865819 (737747-1034998) | 1122.72 (944.59-1339.68) | 1435553 (979763-1759863) | 1348.42 (956.94-1634.88) | 1.22 (0.98-1.46) |
| Greece | 237506 (207799-270322) | 2603.88 (2252.93-3026.55) | 143443 (117702-191795) | 1569.91 (1265.92-2077.03) | -0.94 (-1.2--0.68) |
| Algeria | 2689258 (2360131-3067221) | 9919.47 (8774.26-11200.73) | 1638385 (1447856-1873668) | 3693.77 (3286.33-4188.3) | -3.12 (-3.15--3.09) |
| Ireland | 74016 (63457-84737) | 2108.68 (1804.34-2414.39) | 59302 (44269-81622) | 1167.1 (883.54-1581.21) | -1.36 (-1.57--1.14) |
| Luxembourg | 6666 (5879-7552) | 1825.92 (1605.46-2081.29) | 10211 (8928-12059) | 1407 (1236.63-1644.32) | -0.45 (-0.6--0.3) |
| Uruguay | 247028 (213575-288711) | 8280.53 (7141.47-9686.21) | 147274 (131078-166340) | 4810.64 (4217.05-5484.53) | -1.7 (-1.97--1.43) |
| Spain | 1085220 (936506-1235295) | 2889.09 (2495-3296.68) | 873536 (771928-994914) | 1759.27 (1536.8-2024.42) | -0.99 (-1.19--0.78) |
| Portugal | 390591 (346176-443338) | 4252.77 (3733.77-4877.25) | 225090 (201886-251053) | 2085.59 (1834.7-2353.08) | -1.78 (-1.98--1.57) |
| Suriname | 39306 (34527-44358) | 9858.79 (8761.59-11038.85) | 26471 (23165-30209) | 4631.2 (4053.75-5267.77) | -2.5 (-2.56--2.45) |
| Venezuela (Bolivarian Republic of) | 1720607 (1524683-1934483) | 8971.67 (8056.42-10012.14) | 1705864 (1497843-1917366) | 6504.81 (5707.51-7320.92) | -1.1 (-1.41--0.79) |
| Belize | 24282 (20955-27841) | 12253.36 (10833.85-13890.26) | 21152 (18568-24165) | 4932.24 (4367.44-5551.13) | -2.75 (-2.82--2.68) |
| Sweden | 149399 (128600-173408) | 1851.99 (1603.7-2151.63) | 153068 (121291-209312) | 1344.84 (1079.9-1759.69) | -0.35 (-0.62--0.07) |
| Bahamas | 8477 (7358-9690) | 3268.11 (2891.76-3683.47) | 6226 (5561-6970) | 1634.53 (1457.85-1829.48) | -2.06 (-2.13--2) |
| Barbados | 10899 (9660-12349) | 4383.43 (3893.43-4910.45) | 8102 (7379-8961) | 2718.07 (2445.32-3033.57) | -1.28 (-1.39--1.16) |
| Dominica | 4783 (4127-5459) | 6497.68 (5638.67-7314.19) | 1516 (1348-1728) | 2296.79 (2054.72-2611.45) | -3.23 (-3.35--3.1) |
| Tunisia | 708825 (617579-816690) | 8301.65 (7343.83-9442.4) | 293479 (255890-330726) | 2513.22 (2214.04-2817.31) | -3.81 (-3.93--3.69) |
| Saudi Arabia | 661608 (561807-758404) | 3701.14 (3207.09-4225.67) | 629509 (528834-747473) | 1806.15 (1523.03-2122.24) | -1.43 (-1.77--1.09) |
| United States of America | 4690880 (3924612-5618500) | 1849.31 (1551.92-2197.31) | 5096680 (4232971-6147645) | 1395.06 (1175.42-1648.64) | -0.75 (-1.06--0.43) |
| Bolivia (Plurinational State of) | 758174 (664008-869213) | 10639.07 (9472.57-12052.76) | 683744 (595170-777295) | 5685.73 (4977.56-6432.61) | -2.19 (-2.42--1.96) |
| Dominican Republic | 1033573 (918792-1148130) | 14044.45 (12662.93-15487.1) | 539836 (469731-615078) | 4905.63 (4276.49-5578.75) | -3.6 (-3.7--3.51) |
| Colombia | 2781371 (2419773-3178878) | 8418.45 (7394.35-9522.75) | 1441769 (1271474-1640799) | 2942.99 (2591.67-3317.81) | -3.46 (-3.52--3.41) |
| Trkiye | 8499830 (7696152-9310039) | 14588.49 (13356.21-16039.28) | 3211857 (2853229-3668237) | 3838.21 (3402.4-4341.59) | -4.33 (-4.49--4.17) |
| Saint Lucia | 12672 (11042-14571) | 9026.64 (7972.77-10228.04) | 6855 (6146-7695) | 3856.93 (3408.14-4348.1) | -2.53 (-2.65--2.4) |
| Cuba | 563484 (487927-639439) | 5202.91 (4547.84-5869.45) | 296325 (263007-332637) | 2637.06 (2320.45-2981.17) | -2.33 (-2.44--2.21) |
| Afghanistan | 4871375 (4503859-5227340) | 46107.97 (42653.32-49269.1) | 7893880 (7003998-8812374) | 22439.46 (20337.73-24822.44) | -2.39 (-2.77--2.01) |
| Bangladesh | 23335284 (21143989-25553119) | 18954.78 (17341.35-20820.08) | 7696021 (6917076-8593430) | 4812.64 (4323.23-5336.61) | -4.21 (-4.38--4.03) |
| Brazil | 35771017 (32531763-39341739) | 23671.25 (21641.41-25843.53) | 22520895 (19924002-25629430) | 10270.31 (9076.28-11593.68) | -2.78 (-2.87--2.68) |
| El Salvador | 1043542 (924525-1179659) | 18760.83 (16741.71-21095.93) | 382172 (334768-435430) | 5940.02 (5225.05-6727.94) | -3.77 (-4.01--3.52) |
| Trinidad and Tobago | 64927 (57915-73304) | 5330.29 (4781.94-5963.06) | 32392 (29355-36141) | 2386.24 (2141.36-2653.41) | -2.62 (-2.68--2.56) |
| Ecuador | 781472 (673412-892237) | 6570.15 (5749.98-7450.89) | 632211 (548620-722776) | 3477.65 (3022.97-3961.9) | -2.22 (-2.53--1.91) |
| Costa Rica | 230880 (199888-267815) | 7368.4 (6397.76-8430.09) | 184602 (161163-207545) | 3854.22 (3375.64-4350.42) | -1.6 (-1.81--1.38) |
| Guatemala | 1443991 (1308347-1587716) | 17241.82 (15845.86-18838.92) | 744405 (663567-848504) | 4866.66 (4351.16-5482.96) | -4.03 (-4.14--3.92) |
| India | 281212700 (257064014-304702551) | 30990.78 (28283.66-33508.25) | 145545615 (130010586-163098158) | 10620.96 (9523.35-11919.26) | -3.46 (-3.73--3.19) |
| Bahrain | 30183 (26755-34178) | 5925 (5294.95-6620.76) | 45528 (40410-51862) | 3034.94 (2717.84-3412.23) | -1.72 (-1.89--1.56) |
| Mexico | 14766354 (13286291-16395255) | 15436.17 (13883.56-17035.44) | 8709494 (7585599-9939088) | 6874.68 (6017.64-7809.91) | -2.35 (-2.5--2.19) |
| Jordan | 487672 (429787-551456) | 12325.11 (11050.3-13666.72) | 631698 (550907-720340) | 5169.39 (4553.98-5821.13) | -2.83 (-3.19--2.46) |
| Palestine | 418297 (368034-473735) | 19232.3 (17132.3-21551.47) | 235543 (202696-272242) | 4538.64 (3983.35-5152.76) | -4.25 (-4.41--4.09) |
| Qatar | 16551 (14402-18896) | 3676.34 (3209.91-4149.73) | 71627 (58485-87309) | 2448.33 (2010.63-2970.73) | -0.76 (-0.96--0.56) |
| Syrian Arab Republic | 1732000 (1508471-1971654) | 12370.68 (10986.78-13896.48) | 730309 (647906-820723) | 5551.21 (4951.83-6178.87) | -2.4 (-2.53--2.26) |
| Nicaragua | 423135 (374211-474908) | 10397.43 (9340.95-11542.68) | 151671 (135724-168451) | 2332.45 (2100.78-2585.19) | -5.06 (-5.5--4.62) |
| Iran (Islamic Republic of) | 5905498 (5113861-6778997) | 9531.15 (8439.46-10769.53) | 2118693 (1875960-2400707) | 2566.36 (2250.25-2911.97) | -3.57 (-3.92--3.22) |
| Honduras | 631096 (555622-712911) | 13064.31 (11664.96-14592.26) | 505622 (437113-573993) | 5075.95 (4445.99-5727.55) | -2.97 (-3.02--2.92) |
| Congo | 1169918 (1081826-1253952) | 43770.93 (40730.08-47055.8) | 1800700 (1631676-1990887) | 31721.84 (29005.68-34754.15) | -0.86 (-1.14--0.59) |
| Paraguay | 708433 (609398-809175) | 16258.52 (14251.77-18334.72) | 466073 (403065-529652) | 6443.47 (5624.6-7273.42) | -2.81 (-2.87--2.75) |
| Egypt | 4710332 (4168974-5274255) | 7940.66 (7103.26-8884.4) | 3122400 (2757543-3527466) | 2901.98 (2568.94-3245.26) | -2.36 (-2.69--2.02) |
| Panama | 161067 (139845-183108) | 6649.39 (5838.68-7494.05) | 115362 (101905-131229) | 2703.23 (2388.86-3078.95) | -2.79 (-3.05--2.53) |
| Comoros | 200018 (183993-217170) | 42049.57 (39104.44-45248.85) | 116331 (102206-130696) | 15718.24 (13899.29-17525.34) | -3.22 (-3.28--3.17) |
| Kuwait | 54341 (47125-63473) | 3096.15 (2731.24-3555.14) | 49397 (41960-58091) | 1118.41 (956.37-1297.69) | -3.36 (-3.45--3.27) |
| Iraq | 2691967 (2343508-3091894) | 13383.44 (11798.4-15156.53) | 1624648 (1417769-1858900) | 3902.98 (3451.35-4406.11) | -3.84 (-4.21--3.47) |
| United Arab Emirates | 134949 (113157-157878) | 6904.07 (5837.04-7997.76) | 481513 (374560-598062) | 5359.62 (4247.42-6621.84) | 0.2 (-0.17-0.58) |
| Angola | 4727144 (4344406-5085842) | 40701.18 (37337.44-43854.09) | 5833293 (5126240-6606918) | 15820.28 (13977.9-17680.1) | -3.16 (-3.35--2.97) |
| Lebanon | 204038 (179414-231472) | 6582.9 (5817.25-7425.45) | 147304 (129459-168196) | 2718.89 (2396.4-3105.75) | -2.72 (-2.96--2.49) |
| Morocco | 4975383 (4406112-5593284) | 18505.85 (16601.47-20565.19) | 2360948 (2083127-2701638) | 6426.71 (5673.39-7338.87) | -3.19 (-3.43--2.95) |
| Nepal | 5413751 (4954881-5892301) | 25525.59 (23376.44-27630.28) | 1923010 (1683870-2149174) | 6214.96 (5493.22-6915.18) | -4.57 (-4.66--4.48) |
| Libya | 313217 (273285-358044) | 6970.61 (6205.88-7868.13) | 180212 (159872-203061) | 2821.09 (2521.56-3167.27) | -2.34 (-2.59--2.09) |
| Oman | 261713 (230892-300424) | 12312.27 (10890.62-14040.26) | 124675 (107166-144339) | 2713.9 (2346.65-3123.94) | -4.33 (-4.67--3.99) |
| Yemen | 6093670 (5558178-6606459) | 40377.99 (37364.56-43528.64) | 4673553 (4142674-5348798) | 12960.88 (11637.59-14631.89) | -3.8 (-3.88--3.71) |
| Burundi | 2465368 (2285234-2679335) | 43906.05 (40814.52-47373.78) | 2744341 (2449528-3085389) | 22072.28 (19934.47-24428.87) | -2.54 (-2.73--2.36) |
| Malawi | 5416269 (5057553-5756540) | 54205.28 (50994.6-57005.28) | 4222315 (3746515-4731201) | 22116.9 (20021.2-24295.95) | -3.07 (-3.29--2.84) |
| Bhutan | 174092 (155816-194131) | 25820.22 (23382.14-28451.66) | 42078 (36979-47646) | 5616.02 (4993.86-6335.5) | -4.96 (-5.15--4.76) |
| Pakistan | 22256316 (20072290-24678864) | 17131.03 (15488.51-18783.73) | 10975268 (9591544-12349522) | 4404.01 (3885.47-4919.47) | -3.81 (-4.08--3.55) |
| Democratic Republic of the Congo | 15164899 (13795476-16462940) | 35321.19 (32521.18-38048.81) | 24700165 (21842708-27645155) | 25307.95 (22591.28-28050.43) | -0.93 (-1.47--0.39) |
| Central African Republic | 1316278 (1231682-1406093) | 43888.85 (40980.92-46488.92) | 1827472 (1657598-2017590) | 30527.25 (27834.48-33510.09) | -1.12 (-1.16--1.08) |
| Equatorial Guinea | 206365 (190523-221348) | 42456.83 (39191.39-45590.87) | 94736 (80047-109829) | 5710.43 (4954.91-6491.78) | -6.97 (-7.22--6.71) |
| Gabon | 176723 (155106-199866) | 16108.02 (14340.98-18207) | 120196 (103927-139552) | 6284.44 (5515.17-7210.27) | -2.72 (-2.93--2.52) |
| Rwanda | 2703812 (2455882-2960673) | 36079.1 (32935.6-39202.97) | 2054556 (1819337-2324023) | 15485.62 (13804.5-17347.42) | -3.12 (-3.41--2.82) |
| Uganda | 6443542 (5848086-7006013) | 37413.01 (34452.7-40207.56) | 4433309 (3874439-5116731) | 10029.67 (8892.75-11402.04) | -4.44 (-4.56--4.32) |
| Djibouti | 148619 (134505-163926) | 35260.95 (32425.59-38096.02) | 198044 (176967-225816) | 15674.32 (14118.62-17669.22) | -2.71 (-2.86--2.56) |
| Eritrea | 1906723 (1754302-2039818) | 53865.19 (50221.87-57115.89) | 1450606 (1282098-1633450) | 21620.07 (19189.91-24049.13) | -2.85 (-3.02--2.68) |
| Kenya | 13424944 (12593234-14310623) | 54731.85 (51451.69-58076.28) | 12335925 (10670502-13892448) | 24032.94 (21199.38-26738.03) | -2.48 (-2.73--2.24) |
| Madagascar | 4678569 (4234715-5088377) | 38072.75 (34742.05-41129.95) | 6025799 (5352128-6747259) | 20941.16 (18986.34-23044.73) | -1.97 (-2.21--1.73) |
| Ethiopia | 30054814 (28215411-31750712) | 58373.93 (55335.34-61511.14) | 21480763 (19021013-24082579) | 20129.1 (18108.47-22200.32) | -3.79 (-4.13--3.45) |
| Zambia | 2992717 (2713907-3290293) | 35692.55 (32577.19-39081.03) | 2426532 (2101805-2777827) | 12184.67 (10792.54-13637.99) | -3.66 (-4.02--3.31) |
| Mauritius | 106148 (93746-120331) | 9321.67 (8292.45-10494.56) | 33001 (29342-36929) | 2949.2 (2581.23-3309.51) | -3.51 (-3.69--3.34) |
| Namibia | 257040 (226499-290272) | 15461.73 (13717.57-17436.76) | 170415 (147265-195766) | 6519.39 (5715.89-7405.2) | -2.82 (-2.96--2.69) |
| Cameroon | 6940576 (6661298-7209260) | 63668.24 (61466.2-65975.32) | 6441498 (5645853-7308004) | 19429.98 (17341.3-21646.7) | -3.61 (-3.85--3.37) |
| Guinea-Bissau | 564580 (527788-595749) | 50443.74 (47237.53-53035.15) | 516840 (459348-580600) | 22603.27 (20239.83-25019.2) | -2.41 (-2.48--2.34) |
| Senegal | 3915105 (3705206-4148321) | 47625.27 (45086.94-50111.97) | 1485490 (1291957-1717667) | 9145.38 (8070.97-10405.33) | -4.7 (-5.1--4.3) |
| Togo | 1644848 (1526258-1759770) | 39844.76 (37352.12-42504.85) | 1483392 (1288105-1682230) | 16439.94 (14494.26-18598.49) | -2.63 (-2.74--2.52) |
| Lesotho | 549306 (498395-603583) | 30166.94 (27511.95-32904.67) | 255279 (222313-292998) | 12501.88 (10902.28-14213.35) | -2.77 (-2.82--2.72) |
| Mozambique | 8182675 (7709377-8611951) | 59639.45 (56548.74-62425.76) | 8172074 (7230197-9046662) | 26230.48 (23756.97-28833.44) | -2.74 (-2.88--2.6) |
| Botswana | 371068 (332019-410619) | 24678.42 (22325.04-26912.43) | 201850 (174075-231470) | 8176.45 (7076.24-9344.84) | -3.43 (-3.52--3.35) |
| Zimbabwe | 2972765 (2686997-3306929) | 24944.33 (22681.76-27464.22) | 2992394 (2633386-3444943) | 17547.01 (15694.26-19849.6) | -0.61 (-0.86--0.36) |
| Seychelles | 5361 (4578-6171) | 6773.45 (5842.96-7750.04) | 1941 (1724-2187) | 2010.87 (1776.92-2284.84) | -3.44 (-3.77--3.11) |
| Eswatini | 213192 (189370-239433) | 21002.89 (18864.9-23336.13) | 96808 (82698-112452) | 7512.8 (6495.62-8653.49) | -3.17 (-3.25--3.1) |
| Cook Islands | 2833 (2446-3283) | 13763.65 (12067.39-15758.64) | 614 (539-701) | 3863.98 (3367.68-4435.29) | -4 (-4.05--3.95) |
| United Republic of Tanzania | 7384421 (6680379-8105742) | 28435.35 (26010.49-31034.01) | 6389771 (5663748-7280210) | 11129.78 (9923.16-12494.6) | -2.95 (-3.2--2.69) |
| Bermuda | 1900 (1695-2134) | 3217.9 (2868.24-3673.94) | 1155 (1044-1279) | 1724.75 (1545.25-1910.2) | -2.1 (-2.17--2.04) |
| Benin | 2713670 (2562305-2881253) | 49553.44 (46839.68-52368.71) | 3630174 (3211266-4068432) | 23525.24 (20845.09-26051.38) | -2.3 (-2.38--2.21) |
| Somalia | 7521926 (7366463-7657582) | 93700.45 (92122.57-94954.89) | 18350612 (17801435-18871516) | 83940.53 (81835.2-85919.55) | -0.36 (-0.39--0.34) |
| Saint Kitts and Nevis | 2717 (2385-3085) | 6522.62 (5779.6-7347.84) | 1558 (1412-1715) | 2603.56 (2346.74-2871.53) | -2.85 (-2.96--2.75) |
| South Africa | 5455333 (4805638-6101040) | 12383.54 (10972.35-13757.22) | 2479272 (2138832-2903972) | 4476.87 (3840.68-5251.53) | -3.26 (-3.3--3.22) |
| Mali | 6168575 (5930095-6432093) | 65755.91 (63313.45-68308.32) | 8026144 (7167842-8930451) | 29757.27 (27007.18-32864.04) | -2.55 (-2.62--2.49) |
| Sudan | 6150893 (5531935-6734465) | 27731.84 (25119.54-30241.22) | 3794951 (3281269-4354682) | 8257.58 (7275.21-9297.74) | -3.79 (-4.07--3.51) |
| Cabo Verde | 107900 (96163-119341) | 27776.54 (25177.12-30672.12) | 30484 (26606-34678) | 5457.62 (4816.05-6189.47) | -5.29 (-5.46--5.12) |
| Chad | 4514757 (4336772-4696903) | 68819.93 (66078.61-71365.29) | 7474329 (6830310-8188772) | 36706.18 (33813.2-39879.33) | -2.05 (-2.13--1.96) |
| Burkina Faso | 6701095 (6387987-6978801) | 64697.25 (61857.73-67443.8) | 6292830 (5595584-6963264) | 25305.35 (22719.04-27786.11) | -3.16 (-3.24--3.07) |
| Gambia | 518636 (481227-552592) | 48548.17 (45356.85-51590.46) | 535837 (472291-602193) | 20765.74 (18741.37-23070.19) | -2.67 (-2.7--2.64) |
| Cte d'Ivoire | 4716585 (4331990-5137700) | 32423.5 (29909.67-34907.63) | 3979559 (3440299-4532133) | 12633.55 (11023.06-14228.18) | -2.8 (-2.89--2.72) |
| Guinea | 3516342 (3323871-3714616) | 53641.76 (50705.73-56674.87) | 3254409 (2878817-3699433) | 21576.67 (19439.34-23957.6) | -2.83 (-2.9--2.76) |
| Sao Tome and Principe | 50420 (46292-54492) | 37063.17 (34148.86-39768.89) | 24802 (21633-28361) | 10891.41 (9601.14-12351.54) | -4.04 (-4.23--3.85) |
| American Samoa | 6049 (5242-6916) | 10957.16 (9568.97-12505.38) | 2878 (2449-3332) | 5922.17 (5053.16-6850.86) | -1.83 (-1.98--1.68) |
| Liberia | 971200 (896957-1045675) | 35660.82 (33190.66-38346.38) | 862540 (739939-991386) | 14968.42 (13003.23-17068.43) | -2.93 (-3.08--2.78) |
| San Marino | 340 (288-418) | 1498.22 (1267.22-1805.19) | 450 (356-662) | 1215.06 (973.36-1713.95) | -0.16 (-0.33-0.02) |
| Tokelau | 313 (273-354) | 18216.05 (16179.61-20358.54) | 100 (86-116) | 7454.15 (6459.11-8622.39) | -2.86 (-2.94--2.77) |
| Tuvalu | 3149 (2841-3466) | 30285.44 (27129.29-33380.51) | 1759 (1507-2007) | 13682.94 (11778.55-15597.14) | -2.27 (-2.38--2.17) |
| United States Virgin Islands | 4394 (3871-4976) | 4108.71 (3633.62-4644.75) | 1591 (1447-1761) | 1703.69 (1541.08-1890.06) | -2.94 (-3.08--2.79) |
| Ghana | 7341839 (6827438-7845005) | 44450.31 (41410.54-47381.2) | 6654644 (5858081-7519994) | 18006.01 (15935.27-20126.69) | -2.96 (-3.09--2.84) |
| Mauritania | 739303 (680353-803514) | 31057.18 (28560.57-33841.62) | 489193 (429417-558339) | 10252.01 (9157.11-11596.56) | -3.25 (-3.35--3.16) |
| Niger | 6777267 (6550818-6946614) | 78475.94 (76130.44-80516.11) | 13967810 (12982884-14953973) | 49780.97 (46485.54-52807.7) | -1.43 (-1.48--1.39) |
| Sierra Leone | 2213255 (2064987-2327561) | 48523.49 (45634.38-51197.13) | 1943008 (1711084-2207641) | 19904.67 (17876.31-22345.48) | -2.89 (-3.1--2.67) |
| Greenland | 852 (734-968) | 1559.21 (1357.32-1769.39) | 626 (543-811) | 1126.21 (979.17-1420.03) | -1.37 (-1.61--1.13) |
| Nigeria | 16991505 (15052716-18754707) | 16820.94 (14830.24-18538.02) | 16115401 (14107912-18508621) | 6540.55 (5781.76-7458.88) | -3.14 (-3.52--2.76) |
| Nauru | 1815 (1577-2072) | 15392.52 (13541.86-17393.32) | 1254 (1064-1452) | 10095.8 (8665.03-11646.75) | -1.22 (-1.68--0.75) |
| Puerto Rico | 145213 (128295-164330) | 4037.95 (3558.94-4559.78) | 64246 (58016-71562) | 1776.1 (1605.62-1971.14) | -2.58 (-2.69--2.46) |
| Guam | 10937 (9458-12689) | 7480.27 (6501.44-8656.68) | 4861 (4323-5623) | 3242.53 (2835.75-3777.99) | -2.71 (-2.85--2.57) |
| Monaco | 377 (298-522) | 1241.76 (999.94-1601.3) | 483 (349-676) | 1103.3 (800.35-1500.06) | 0.13 (-0.1-0.36) |
| Northern Mariana Islands | 3020 (2617-3478) | 6318.26 (5535.47-7278.53) | 2038 (1795-2308) | 4508.2 (3961.44-5127.97) | -0.63 (-0.82--0.43) |
| Palau | 1373 (1184-1588) | 8519.08 (7422.59-9800.35) | 750 (648-860) | 4863.76 (4134.81-5658.03) | -1.53 (-1.66--1.4) |
| South Sudan | 3190779 (2948584-3438499) | 53420.54 (50136.94-57081.46) | 3482648 (3160983-3854823) | 35775.01 (32737.42-38818.18) | -1.54 (-1.64--1.44) |
| Niue | 309 (264-360) | 12886.76 (11077.89-14755.6) | 89 (77-101) | 5799.23 (5020.27-6636.36) | -2.46 (-2.53--2.4) |

## Table S3：Prevalence of Nutritional Deficiencies in cases and age-standardized rates for both sexes combined in 1990 and 2021, with percentage change between 1990 and 2021 by GBD 204 nations.

| Prevalence | | | | | |
| --- | --- | --- | --- | --- | --- |
| nations | **1990** | | **2021** | | **1990-2021 EAPC** |
|  | **All-ages cases(both sexs)** | **Age-standardized rates per 100,000 people** | **All-ages cases(both sexs)** | **Age-standardized rates per 100,000 people** |  |
|  | **n (95% UI)** | **n (95% UI)** | **n (95% UI)** | **n (95% UI)** | **n (95% UI)** |
| Slovenia | 374344 (347394-413101) | 19631.77 (18200.01-21658.35) | 236943 (218530-258509) | 11853.65 (10950.59-12900.9) | -0.72 (-0.75--0.69) |
| North Macedonia | 762149 (711721-821017) | 38337.51 (35878.23-41250.61) | 471764 (437079-507330) | 22353.3 (20726.51-24154.67) | -1.68 (-1.84--1.53) |
| Australia | 928724 (785093-1219108) | 5641.14 (4594.01-7877) | 1201229 (1063622-1456161) | 4453.22 (3786.33-6069.07) | -1.24 (-1.26--1.22) |
| Lithuania | 521093 (484826-565640) | 14306.53 (13281.18-15520.27) | 283887 (262311-310251) | 10457.76 (9607.15-11415.08) | -2.02 (-2.26--1.77) |
| New Zealand | 230052 (197410-294178) | 6957.49 (5812.44-9376.4) | 258369 (221592-325652) | 5224.19 (4198-7230.14) | -2.75 (-2.81--2.69) |
| Japan | 10491456 (8806964-12639362) | 8444.46 (7104.56-10181.46) | 9630525 (8488688-11196238) | 6477.16 (5533.06-7783.1) | -1.55 (-1.63--1.47) |
| Andorra | 3203 (2881-3642) | 6290.34 (5695.34-7136.69) | 4322 (3878-4884) | 4885.97 (4394.91-5510.26) | -0.79 (-0.82--0.76) |
| Taiwan (Province of China) | 2614677 (2413857-2879131) | 13191.99 (12212.24-14369.26) | 1891722 (1733771-2058030) | 7570.22 (6975.27-8143.17) | -0.45 (-0.51--0.38) |
| Democratic People's Republic of Korea | 5355537 (4992132-5787604) | 25376.74 (23600-27411.87) | 4225751 (3894052-4557392) | 16967.54 (15671.26-18319.42) | -0.65 (-0.74--0.55) |
| China | 273163013 (257413969-290858168) | 23093.68 (21856.9-24477.24) | 146147146 (138504726-154667650) | 9890.92 (9410.55-10463.85) | -1.83 (-1.86--1.81) |
| Argentina | 7047807 (6119488-8306347) | 21214.1 (18413.33-24925.27) | 6319575 (5182025-8031166) | 14582.7 (11802.47-18744.18) | -1.42 (-1.52--1.32) |
| Malta | 34009 (30607-38759) | 9423.61 (8463.63-10911.21) | 26404 (24154-29046) | 5835.08 (5266.62-6478.9) | -0.5 (-0.56--0.45) |
| Israel | 732123 (656184-818243) | 14441.16 (12970.49-16106.19) | 794123 (724587-883834) | 8259.34 (7534.29-9232.19) | -1.01 (-1.17--0.84) |
| Fiji | 254560 (238748-270273) | 32835.2 (31019.54-34655.07) | 247504 (223258-280761) | 27188.18 (24626.29-30754.71) | -1.93 (-2.11--1.76) |
| Norway | 265892 (239876-300826) | 6471.79 (5797.87-7407.96) | 277447 (247699-311117) | 4813.6 (4288.95-5524.36) | -2.2 (-2.38--2.02) |
| Canada | 1752544 (1308155-2437231) | 6403.97 (4941.3-8668.26) | 1995907 (1582068-2808152) | 4951.54 (3898.01-7332.88) | -0.91 (-0.98--0.84) |
| Belarus | 1563748 (1449199-1681683) | 15093.4 (13985.02-16212.33) | 964966 (885371-1047828) | 10287.4 (9441.72-11380.56) | -1.33 (-1.42--1.25) |
| Papua New Guinea | 1542881 (1421218-1705185) | 36768.91 (34238.96-39919.63) | 3109379 (2758739-3531382) | 28736.05 (25722.53-32239.47) | -2.23 (-2.34--2.12) |
| United Kingdom | 4093691 (3791007-4474315) | 7581.15 (7005.13-8295.82) | 3787955 (3414244-4247306) | 5772.62 (5174.93-6629.83) | -2.6 (-2.79--2.41) |
| Armenia | 611495 (571687-658366) | 17722.32 (16560.01-19013.7) | 412363 (381082-447030) | 14256.66 (13198.92-15474.82) | -1.49 (-1.61--1.38) |
| Lao People's Democratic Republic | 2340345 (2237672-2449816) | 52741.88 (50666.76-55173.72) | 2103017 (1947557-2277055) | 28278.59 (26251.34-30644.07) | -2.21 (-2.29--2.12) |
| Maldives | 112677 (105742-120060) | 43788.99 (41249.65-46518.74) | 94646 (86063-104241) | 19551.99 (17826.64-21798.27) | -2.11 (-2.19--2.03) |
| Timor-Leste | 401358 (370772-434528) | 45356.36 (41594.02-48917.61) | 379794 (351913-414302) | 25372.9 (23661.37-27554.3) | -0.6 (-0.64--0.56) |
| Viet Nam | 18052163 (17111361-19182987) | 25308.51 (24071.05-26811.76) | 13965822 (13046010-14899790) | 14403.07 (13479.69-15355.35) | -2.26 (-2.4--2.12) |
| Micronesia (Federated States of) | 65129 (61713-68604) | 61853.55 (58966.8-64872.92) | 49754 (46656-52997) | 48830.14 (46110.2-51716.97) | -0.32 (-0.52--0.13) |
| Turkmenistan | 1008814 (945444-1086538) | 25646.75 (24236.22-27360.93) | 1037650 (965540-1126831) | 19869.58 (18496.7-21491.31) | -0.88 (-0.94--0.82) |
| Chile | 1838097 (1656697-2029543) | 13699.4 (12397.79-15075.33) | 1336242 (1210620-1461165) | 7219.43 (6542.83-7878.94) | -0.81 (-0.86--0.77) |
| Switzerland | 409555 (312366-581340) | 6101.53 (4751.12-8410.67) | 491590 (386662-689282) | 5212.14 (4077.98-7704.41) | -0.94 (-0.98--0.9) |
| Cambodia | 5523052 (5229493-5848796) | 49060.05 (46653.19-51753.31) | 4554162 (4203268-5038214) | 26723.66 (24691.79-29529.86) | -0.9 (-0.98--0.83) |
| Indonesia | 77497299 (72677788-82473629) | 41014.77 (38766.43-43327.72) | 55662466 (52186551-59414863) | 20728.02 (19497.27-22037.06) | -1.37 (-1.46--1.28) |
| Malaysia | 4650893 (4444605-4871816) | 26018.16 (24923.95-27228.85) | 6879762 (5840570-7999940) | 21332.46 (18490.74-24524.52) | -0.81 (-0.87--0.74) |
| Myanmar | 19663298 (18484461-20980392) | 45886.75 (43343.81-48743.27) | 16329734 (15008406-17883190) | 29213.71 (26911.28-32009.79) | -1.78 (-1.87--1.69) |
| Samoa | 66275 (61552-71576) | 36872.12 (34336.29-39533.25) | 59998 (55300-65571) | 27098.25 (25099.77-29454.74) | -0.64 (-0.72--0.57) |
| Philippines | 21425049 (19991567-22898596) | 32436.66 (30594.28-34489.92) | 23864396 (22392430-25282769) | 21197.35 (19968.71-22396.79) | -0.47 (-0.59--0.35) |
| Thailand | 15311974 (14310314-16393772) | 27449.8 (25882.45-29102.56) | 8977003 (8071167-10138017) | 13278.14 (12224.87-14613.47) | -1.04 (-1.11--0.98) |
| Kiribati | 41589 (39202-43899) | 52869.92 (49856.17-56022.55) | 50466 (46888-54944) | 40743.7 (37942.09-44016.84) | -1.97 (-2--1.93) |
| Georgia | 1033723 (964697-1127273) | 19060.41 (17744.41-20956.05) | 569974 (529359-618183) | 16049.92 (14857.92-17528.32) | -1.7 (-1.76--1.64) |
| Albania | 1403031 (1295905-1531993) | 40885.1 (38011.53-44393.66) | 601342 (562239-647290) | 23533.72 (21999.8-25534.1) | -2.1 (-2.21--1.99) |
| Slovakia | 1347235 (1251588-1451319) | 26033.16 (24212.81-28137.43) | 816650 (758615-894736) | 15518.42 (14365.14-17165) | -1.77 (-1.84--1.7) |
| Solomon Islands | 199136 (188323-210873) | 54739.69 (51780.12-57567.9) | 294131 (270027-317458) | 40959.94 (37896.21-44039.69) | -1.6 (-1.68--1.52) |
| Kyrgyzstan | 1241932 (1153168-1356519) | 26842.96 (25066.05-28996.1) | 1527520 (1413894-1691060) | 21791 (20263.62-23857.79) | -1.69 (-1.78--1.6) |
| Kazakhstan | 6333455 (5927485-6797299) | 37935.95 (35592.18-40483.54) | 4888684 (4466924-5394127) | 25554.56 (23381.18-28322.82) | -2.52 (-2.73--2.32) |
| Vanuatu | 83279 (77711-88871) | 51027.24 (47820.79-54190.13) | 136040 (125511-147200) | 41888.14 (38903.22-45350.84) | -2.01 (-2.11--1.91) |
| Tajikistan | 1819929 (1719370-1924163) | 31686.9 (30081.74-33420.03) | 2899130 (2731115-3081082) | 27878.4 (26338.76-29531.31) | -0.78 (-0.83--0.72) |
| Tonga | 37191 (34044-40314) | 36092.55 (33309.06-39075.35) | 28467 (26232-31152) | 26290.1 (24204.43-28683.37) | -2.16 (-2.35--1.98) |
| Antigua and Barbuda | 14942 (13506-16880) | 24712.23 (22426.02-27631.24) | 15770 (14144-17695) | 18810.92 (16646.55-21726.72) | -0.67 (-0.79--0.54) |
| Marshall Islands | 25701 (24093-27266) | 55194.28 (52263.59-58094.69) | 20968 (19447-22736) | 37397.17 (34995.02-40399.65) | -1.48 (-1.64--1.32) |
| Mongolia | 620273 (589171-652618) | 27285.49 (26065.04-28515.43) | 614434 (577915-653911) | 17967.19 (16929.39-19138.07) | -1.47 (-1.69--1.25) |
| Sri Lanka | 6570531 (6111893-7074531) | 37928.96 (35463.82-40619.32) | 4360400 (4003307-4768814) | 19782.04 (18245.98-21667.72) | -0.57 (-0.64--0.49) |
| Bosnia and Herzegovina | 1635321 (1528821-1753913) | 36452.67 (34137.39-39106.16) | 672789 (623680-735106) | 21228.39 (19615.16-23222.59) | -1.02 (-1.06--0.98) |
| Bulgaria | 2382970 (2211768-2557142) | 28699.68 (26689.84-30856.45) | 1372629 (1281586-1493213) | 21329.52 (19845.32-23174.98) | -0.86 (-0.98--0.74) |
| Estonia | 224652 (209194-242112) | 14440.39 (13394.65-15556.63) | 122245 (112026-132688) | 9303.11 (8605.97-10074.47) | -2.31 (-2.47--2.15) |
| Serbia | 3967302 (3711321-4244742) | 41873.53 (39174.53-44771.42) | 1929464 (1786126-2090314) | 22360.14 (20618.73-24324.59) | -0.7 (-0.78--0.62) |
| Azerbaijan | 1901320 (1793264-2033943) | 25145.76 (23781.82-26770.23) | 2035044 (1907125-2196311) | 19557.6 (18310.26-21043.37) | -1.6 (-1.68--1.52) |
| Guyana | 300922 (275390-328491) | 37422.36 (34490.39-40675.17) | 185890 (171459-209124) | 24347.87 (22472.5-27370.29) | -1.07 (-1.23--0.92) |
| Haiti | 3376601 (3195512-3559661) | 50987 (48598.8-53463.75) | 5533231 (5162392-5982007) | 41988.14 (39321.3-45044.29) | -1.24 (-1.34--1.14) |
| Grenada | 26933 (25326-28639) | 30228.59 (28572.73-31995.94) | 19150 (17480-21194) | 19326.3 (17539.46-21611.39) | -0.72 (-0.79--0.65) |
| Jamaica | 603745 (553262-662886) | 24895.92 (23003.07-27142.5) | 516952 (473950-578572) | 19394.64 (17636.55-21912.96) | -2.12 (-2.31--1.93) |
| Uzbekistan | 7763297 (7192399-8489801) | 34838.68 (32568.59-37483.45) | 9819397 (8977459-10977189) | 28106.35 (25713.22-31433.66) | -1.04 (-1.12--0.97) |
| Poland | 11152678 (10352886-12021048) | 29791.84 (27637.93-32202.95) | 5931335 (5439446-6542449) | 16179.9 (14733.64-18132.66) | -0.96 (-1--0.92) |
| Saint Vincent and the Grenadines | 32731 (29876-36259) | 29391.02 (27145.02-32018.95) | 23685 (21339-26741) | 21648.24 (19562.73-24609.24) | -1.34 (-1.49--1.19) |
| Czechia | 2299720 (2129869-2490297) | 23187.82 (21494.48-25152.72) | 1443954 (1345355-1560926) | 13819.61 (12769.71-15093.34) | -0.93 (-1--0.86) |
| Brunei Darussalam | 29244 (26121-33768) | 11261.93 (10250.22-12620.04) | 35195 (31693-41480) | 7975.23 (7201.99-9245.26) | -1.11 (-1.19--1.04) |
| Hungary | 2546189 (2355394-2754020) | 25613.05 (23627.28-27782.7) | 1429908 (1320166-1568322) | 15546.75 (14317.12-16907.73) | -1.65 (-1.79--1.52) |
| Croatia | 1115532 (1036097-1201588) | 23640.33 (21994.13-25471.46) | 606016 (563643-656979) | 14939.76 (13927.37-16316.09) | -0.47 (-0.73--0.22) |
| Ukraine | 7516437 (7068183-8025042) | 14673.39 (13832.87-15706.78) | 5151748 (4774317-5539074) | 12467.42 (11657.47-13344.38) | -1.4 (-1.49--1.32) |
| Latvia | 382626 (353419-414370) | 14515.09 (13413.01-15678.53) | 197063 (180170-217809) | 10588.87 (9675.24-11680.95) | -1.71 (-1.86--1.55) |
| Montenegro | 141296 (131065-153033) | 22771.12 (21192.27-24682.19) | 98209 (90955-106406) | 16349.69 (15167.88-17863.34) | -1.5 (-1.59--1.41) |
| Romania | 7009981 (6553081-7491797) | 30730.72 (28712.23-32819.36) | 3333896 (3092929-3628451) | 18363.13 (16885.62-20058.78) | -1 (-1.03--0.98) |
| Singapore | 448237 (314281-580363) | 13727.98 (10298.6-17274.58) | 397585 (278717-591232) | 6816.79 (4773.12-10402.84) | -1.19 (-1.26--1.12) |
| Republic of Moldova | 838644 (772539-931514) | 18834.35 (17343.32-20877.34) | 475256 (440345-519739) | 12889.31 (11966.52-14067.12) | -0.57 (-0.61--0.53) |
| Russian Federation | 21620345 (19779916-23482220) | 14352.25 (13233.49-15541.52) | 16080905 (14596187-17916166) | 10851.81 (9938.84-11933.66) | -2.04 (-2.15--1.93) |
| Iceland | 14803 (13552-16225) | 5932.63 (5419.8-6544.53) | 15605 (14069-17945) | 4314.48 (3900.36-4956.91) | -1.47 (-1.53--1.41) |
| Finland | 351777 (324158-384699) | 7213.11 (6673.17-7945.32) | 275519 (250429-299854) | 4907.18 (4434.73-5391.85) | -0.74 (-0.76--0.72) |
| France | 4067607 (3746411-4442689) | 6950.47 (6377.85-7707.07) | 4292685 (3849981-5096010) | 5709.74 (5154.33-6623.07) | -0.64 (-0.78--0.5) |
| Belgium | 703395 (653938-769256) | 7294.58 (6720.9-8122.31) | 719144 (649897-836281) | 5749 (5187.49-6522.86) | -1.01 (-1.1--0.93) |
| Netherlands | 1020115 (924337-1149734) | 6918.6 (6273.92-7769.59) | 1008368 (883269-1136055) | 5457.55 (4855.85-6058.88) | -0.63 (-0.69--0.58) |
| Austria | 528535 (474789-604668) | 7167.42 (6413.55-8381.43) | 458174 (416156-506715) | 5000.16 (4511.27-5601.29) | -0.7 (-0.84--0.56) |
| Italy | 7091237 (6390399-7888575) | 12155.12 (10952.41-13499.73) | 5238282 (4723754-5840810) | 7895.63 (7109.97-9015.37) | -0.71 (-0.77--0.66) |
| Denmark | 345599 (314648-390412) | 6980.66 (6222.34-8083.66) | 310666 (281169-363297) | 5122.73 (4615.71-6179.42) | -0.08 (-0.28-0.12) |
| Cyprus | 66642 (58958-77939) | 8854.09 (7821.48-10404.75) | 71365 (65171-78347) | 5141.56 (4664.01-5716.72) | -0.74 (-0.8--0.68) |
| Peru | 6603264 (6015203-7315396) | 28875.27 (26504.94-31872.66) | 5709077 (5229681-6514241) | 15846.3 (14524.4-18280.11) | -0.91 (-0.95--0.87) |
| Republic of Korea | 4204478 (3646862-5011235) | 9924.44 (8736.72-11562.95) | 2452409 (2234021-2682378) | 4533.18 (4128.41-4996.75) | -1.1 (-1.21--0.99) |
| Germany | 5738260 (4416612-7892066) | 7319.81 (5681.19-9865.68) | 4990151 (4024021-6658683) | 5434.19 (4385-7563.19) | -0.03 (-0.15-0.08) |
| Greece | 760781 (690758-858005) | 7768.87 (6934.74-8797.14) | 631073 (567277-710774) | 6044.32 (5425.87-6861.48) | -1.34 (-1.46--1.22) |
| Algeria | 7656382 (7000215-8478489) | 29442.27 (27066.82-32125.11) | 8322545 (7645635-9357720) | 18801.23 (17311.83-21040.09) | -2.31 (-2.34--2.28) |
| Ireland | 265143 (239782-303889) | 7570.09 (6817.3-8742.93) | 236779 (212091-267186) | 4698.85 (4241.32-5294.82) | -1.46 (-1.55--1.37) |
| Luxembourg | 26466 (23830-30649) | 7111.13 (6392.42-8392.73) | 34327 (31451-37629) | 4998.92 (4572.15-5507.97) | -0.7 (-0.79--0.61) |
| Uruguay | 501254 (409240-618120) | 16625.96 (13494.9-20578.21) | 334651 (283764-421204) | 10631.83 (8802.8-13671.98) | -0.86 (-0.9--0.83) |
| Spain | 3090475 (2801264-3459828) | 8265.55 (7459.58-9669.47) | 2718079 (2496671-2986836) | 5576.01 (5075.52-6193.14) | -0.68 (-0.74--0.61) |
| Portugal | 1106769 (1011074-1264051) | 11509.41 (10389.52-13192.33) | 786211 (721750-856974) | 6991.03 (6376.31-7708.2) | -1.07 (-1.17--0.97) |
| Suriname | 117173 (106312-129328) | 29655.84 (26990.85-32422.21) | 131215 (119534-147008) | 23176.3 (21118.47-26073.82) | -0.99 (-1.07--0.91) |
| Venezuela (Bolivarian Republic of) | 3870817 (3340084-4372221) | 19791.92 (17495.24-22016.69) | 3892986 (3515957-4466593) | 14899.09 (13410.64-17122.43) | -1.46 (-1.57--1.35) |
| Belize | 59522 (54333-65542) | 30290.89 (27982.84-33209.84) | 97854 (88306-111492) | 22748.19 (20609.05-25827.8) | -0.68 (-0.77--0.59) |
| Sweden | 580503 (523428-653965) | 6837.87 (6141.48-7875.52) | 567893 (508071-652813) | 5205.74 (4622.27-6095.44) | -1.21 (-1.28--1.14) |
| Bahamas | 55010 (49609-64169) | 21080.14 (19147.11-24267.2) | 64310 (56765-73550) | 17373.9 (15306.68-19928.72) | -0.86 (-0.91--0.8) |
| Barbados | 51024 (47138-56881) | 20766.55 (19131.78-23358.64) | 45519 (41567-50585) | 16605.96 (15044.02-19024.58) | -1.25 (-1.32--1.17) |
| Dominica | 17655 (16382-19239) | 24169.72 (22506.32-26070.42) | 11754 (10736-12941) | 18152.54 (16712.32-19715.32) | -0.81 (-0.89--0.72) |
| Tunisia | 1866769 (1751587-1983742) | 22113.24 (20877.06-23449.74) | 1613109 (1486935-1745311) | 13738.45 (12725.04-14848.2) | -0.92 (-0.97--0.87) |
| Saudi Arabia | 3136917 (2956287-3332173) | 19256.19 (18080.56-20496.51) | 4571788 (4122436-5233589) | 12526.8 (11462.94-13972.98) | -0.65 (-0.69--0.62) |
| United States of America | 14541231 (13533716-15579743) | 5593.81 (5211.51-6010.07) | 18314001 (16747991-20055378) | 5145.91 (4697.27-5629.11) | -1.42 (-1.45--1.39) |
| Bolivia (Plurinational State of) | 2278064 (2037102-2517408) | 32907.45 (29101.84-36733.66) | 2588907 (2110117-3154442) | 21604.16 (17918.98-25895.84) | -1.6 (-1.72--1.48) |
| Dominican Republic | 2278179 (2127146-2421451) | 31104.66 (29180.42-33029.15) | 2121289 (1982000-2295679) | 19238.44 (17984.89-20773.35) | -0.89 (-0.99--0.8) |
| Colombia | 6083089 (5571505-6686220) | 18036.24 (16644.04-19571.41) | 4294935 (4029325-4621112) | 8999.39 (8437.06-9655.75) | -2.24 (-2.37--2.12) |
| Trkiye | 18835807 (17603088-20182195) | 32370.45 (30438.03-34570.06) | 13297503 (12319472-14561531) | 16103.93 (14926.31-17566.84) | -2.36 (-2.58--2.15) |
| Saint Lucia | 39844 (36440-43871) | 28483.47 (26266.96-31406.13) | 34185 (31132-38199) | 20159.25 (18264.68-22964.82) | -1.56 (-1.64--1.49) |
| Cuba | 2067888 (1875467-2288126) | 19171.46 (17407.28-21147.87) | 1567229 (1435169-1746131) | 14826.61 (13508.09-16876.92) | -1.69 (-1.95--1.43) |
| Afghanistan | 5774398 (5469737-6070045) | 56252.73 (53538.84-58973.48) | 11206690 (10446496-12072159) | 34292.42 (32292.92-36605.91) | -1.01 (-1.17--0.85) |
| Bangladesh | 54653854 (51582218-58332127) | 49519.32 (47120.36-52277.14) | 46439098 (43112388-50854034) | 28727.7 (26753.55-31303.43) | -1.87 (-1.93--1.81) |
| Brazil | 57504906 (54032461-61185283) | 38245.31 (36098.01-40706.12) | 50594193 (46730072-54391641) | 23180.86 (21425.54-25010.78) | -2.14 (-2.31--1.96) |
| El Salvador | 1466432 (1351554-1605837) | 26358.89 (24508.9-28612.86) | 812404 (753287-891087) | 12742.32 (11834.08-13959.11) | -1.39 (-1.48--1.31) |
| Trinidad and Tobago | 306120 (278554-345292) | 25038.98 (22884.95-27801.8) | 237999 (211435-269187) | 18209.05 (16278.68-20673.17) | -1.09 (-1.18--1.01) |
| Ecuador | 1602788 (1405272-1944167) | 14439.51 (12826.35-16964.47) | 1462605 (1361599-1581255) | 8199.5 (7638.72-8872.17) | -1.59 (-1.67--1.51) |
| Costa Rica | 510268 (456530-567590) | 16192.11 (14447-18180.24) | 505555 (443719-593624) | 10879.49 (9646.75-12542.5) | -2.17 (-2.35--1.97) |
| Guatemala | 2703885 (2532256-2901531) | 31614.39 (29830.9-33577.27) | 2988146 (2782023-3260122) | 18931.44 (17688.97-20484.33) | -1.58 (-1.6--1.56) |
| India | 512507031 (496200362-528690232) | 59921.16 (58147.88-61678.27) | 586083606 (569077238-602972817) | 42472.92 (41333.3-43606.53) | -2.23 (-2.3--2.15) |
| Bahrain | 118262 (106168-132528) | 22791.35 (20799.44-24893.55) | 208011 (186130-234467) | 14098.38 (12734.82-15921.24) | -1.53 (-1.56--1.5) |
| Mexico | 20396599 (18961327-21886905) | 21988.73 (20583.86-23425.06) | 16109138 (15059571-17283599) | 12818.47 (11989.89-13725.33) | -1.44 (-1.49--1.4) |
| Jordan | 1141701 (1059276-1240094) | 29509.67 (27662.15-31858.87) | 2414281 (2272617-2568130) | 19689.53 (18610.66-20834.27) | -1.69 (-1.83--1.55) |
| Palestine | 700671 (646701-759936) | 33075.6 (30919.26-35662.59) | 807423 (742617-898200) | 15819.14 (14749.54-17156.57) | -1.67 (-1.7--1.64) |
| Qatar | 81622 (73638-91273) | 18192.1 (16693.65-20004.73) | 321770 (287762-368159) | 11434.85 (10441.84-12554.26) | -1.64 (-1.69--1.6) |
| Syrian Arab Republic | 4186920 (3881680-4499691) | 31729.01 (29553.68-33719.35) | 2819487 (2587693-3086660) | 21218.3 (19609.38-23252.52) | -1.17 (-1.26--1.08) |
| Nicaragua | 838499 (777231-910891) | 20548.49 (19203.23-21887.88) | 719235 (666480-776872) | 10951.61 (10200.55-11780.18) | -1.7 (-1.87--1.52) |
| Iran (Islamic Republic of) | 14998111 (13980519-16153123) | 25392.31 (23887.22-26990.17) | 11639326 (10748989-12572016) | 13906.44 (12909.91-14953.84) | -1.88 (-2.01--1.75) |
| Honduras | 1222661 (1142914-1308894) | 24750.98 (23390.12-26326.02) | 1570100 (1452834-1710599) | 15603.27 (14524.89-16924.19) | -1.16 (-1.34--0.98) |
| Congo | 1551020 (1467975-1636203) | 62235.96 (59168.66-65295.65) | 2860303 (2675174-3050325) | 52061.82 (48981.89-55206.25) | -1.64 (-1.73--1.55) |
| Paraguay | 1221491 (1121348-1323055) | 28611.46 (26539.98-30784.22) | 1252201 (1157257-1381071) | 17413.14 (16092.68-19135.8) | -1.77 (-1.81--1.74) |
| Egypt | 15297420 (14244897-16664156) | 26868.66 (25185.84-28853.56) | 17844360 (16674194-19262249) | 17118.03 (16029.1-18565.58) | -0.99 (-1.15--0.82) |
| Panama | 452283 (400734-515730) | 18527.12 (16572.82-20701.4) | 491373 (429694-580577) | 11645.51 (10205.69-13703.1) | -1.3 (-1.37--1.22) |
| Comoros | 271741 (237107-294866) | 57822.44 (51376.55-61984.84) | 259037 (215530-290709) | 34886.53 (29444.27-38877.79) | -1.16 (-1.22--1.1) |
| Kuwait | 307909 (283437-335157) | 16886 (15697.74-18178.74) | 540996 (487976-616946) | 11635.63 (10617.22-13137.58) | -1.28 (-1.42--1.14) |
| Iraq | 5788074 (5401231-6260840) | 29800.83 (27922.64-31975.09) | 7077956 (6504218-7723804) | 17163.78 (15836.57-18599.99) | -1.46 (-1.56--1.36) |
| United Arab Emirates | 481036 (440672-530244) | 24819.45 (22825.6-27284.63) | 1603361 (1398260-1847123) | 18742.29 (16773.4-21086.23) | -1.13 (-1.16--1.09) |
| Angola | 6186523 (5838808-6499622) | 58026.3 (54899.55-60626.7) | 11704376 (10805454-12722499) | 35336.58 (32902.64-38487.54) | -1.49 (-1.54--1.43) |
| Lebanon | 732734 (687930-798208) | 23891.37 (22494.32-25726.96) | 799379 (744323-861337) | 14620.66 (13650.57-15817.42) | -1.71 (-1.78--1.64) |
| Morocco | 9707219 (9034825-10460699) | 37068.86 (34554.43-39889.36) | 8030109 (7429378-8715831) | 21786.25 (20145.98-23637.67) | -0.67 (-0.76--0.58) |
| Nepal | 10342769 (9886378-10861753) | 52414.89 (50242.74-54764.26) | 10644947 (9704958-11861653) | 34482.2 (31544.86-37984.47) | -0.59 (-0.63--0.56) |
| Libya | 978796 (891830-1090169) | 22149.45 (20385.36-24356.55) | 1070784 (983164-1180465) | 16062.15 (14846.35-17529.5) | -0.96 (-1--0.92) |
| Oman | 674044 (608346-742653) | 31364.67 (28701.55-34089.97) | 690951 (609795-807294) | 15116.03 (13304.87-17903.98) | -1.52 (-1.65--1.39) |
| Yemen | 8266360 (7883829-8656417) | 57452.18 (55031.26-59943.73) | 14082183 (13481816-14738114) | 40290.46 (38748.39-42169.69) | -1.17 (-1.25--1.09) |
| Burundi | 3061276 (2866328-3267126) | 54981.35 (51756.22-58269.9) | 4991926 (4554230-5465443) | 38429.55 (35682.31-41524.52) | -2.34 (-2.39--2.29) |
| Malawi | 6913620 (6608786-7195308) | 69802.35 (67183.84-72332.49) | 8656674 (7848761-9516021) | 44105.69 (40640.14-47377.53) | -1.69 (-1.73--1.64) |
| Bhutan | 318338 (281419-353467) | 48891.67 (44259.17-53556.86) | 277583 (252613-302064) | 37768.14 (34657.94-40737.8) | -0.65 (-0.68--0.61) |
| Pakistan | 56282807 (53636124-59087165) | 49202.74 (47034.88-51486.89) | 95606526 (89857461-102618553) | 40626.48 (38401.52-43292.45) | -1.69 (-1.77--1.61) |
| Democratic Republic of the Congo | 24262323 (23164877-25455102) | 62916.74 (60572.06-65476.4) | 43605067 (40956206-46426209) | 48162.23 (45383.59-51007.84) | -1.17 (-1.33--1) |
| Central African Republic | 1673037 (1590180-1750113) | 58296.84 (55706.86-60764.26) | 2742190 (2563691-2950267) | 47914.65 (45068.42-50943.92) | -1.67 (-1.77--1.57) |
| Equatorial Guinea | 302134 (288611-314695) | 70364.81 (67337.72-73474.9) | 402994 (344119-465629) | 26158.65 (22742.18-29657.99) | -1.51 (-1.6--1.42) |
| Gabon | 449712 (410482-480971) | 44310.33 (41077.79-47056.2) | 598336 (526751-675306) | 32224.71 (28570.79-35869.92) | -1 (-1.1--0.91) |
| Rwanda | 3628004 (3393168-3860265) | 50111.15 (47124.27-52901.5) | 3832422 (3554499-4183178) | 29601.17 (27609.36-32060.04) | -1.98 (-2.15--1.81) |
| Uganda | 8603437 (7985578-9212640) | 49308.88 (46060-52300.47) | 10753437 (9703234-12105962) | 24726.09 (22656.9-27196.07) | -0.73 (-0.99--0.46) |
| Djibouti | 241318 (226879-258702) | 58904.85 (55704.39-62690.85) | 525637 (476470-574340) | 41958.52 (38064.75-45627.92) | -3.77 (-4.04--3.49) |
| Eritrea | 2324529 (2191587-2442296) | 66900.42 (63877.1-69797.07) | 2786453 (2570787-3060430) | 41555.57 (38467.83-45099.15) | -1.41 (-1.46--1.36) |
| Kenya | 14330096 (13575438-15118290) | 59720.1 (56827.94-62658.57) | 16460223 (15035999-17963779) | 33321.5 (30946.65-35843.52) | -2.02 (-2.22--1.83) |
| Madagascar | 6498307 (6105539-6889650) | 54087.19 (51131.85-56769.07) | 11254650 (10521368-12200681) | 39969.65 (37807.08-42539.97) | -1.14 (-1.24--1.04) |
| Ethiopia | 35494038 (34100133-36882822) | 70658.23 (68429.88-73038.09) | 42839897 (40079302-45821856) | 39962.41 (37439.21-42465.46) | -1.17 (-1.28--1.06) |
| Zambia | 4424120 (4146151-4671321) | 54053.64 (51253.16-56916.46) | 8413147 (7600159-9254158) | 41987.51 (37904.51-46014.03) | -1.22 (-1.31--1.13) |
| Mauritius | 288463 (262322-322296) | 26647.21 (24304.95-29561.82) | 215967 (193604-246240) | 18115.01 (16036.76-21137.13) | -1.57 (-1.62--1.51) |
| Namibia | 584680 (484681-648367) | 38303.33 (31820.52-42170.51) | 529722 (481390-601055) | 20941.33 (19164.5-23376.74) | -0.46 (-0.65--0.28) |
| Cameroon | 7558956 (7337152-7800606) | 70497.23 (68535.98-72574.44) | 11364920 (10373465-12238866) | 34759.75 (32308.99-37056.86) | -0.19 (-0.2--0.18) |
| Guinea-Bissau | 711982 (677740-743084) | 67511.27 (64669.44-70347.25) | 1025659 (956517-1106210) | 47349.41 (44238.08-50568.73) | -0.86 (-0.97--0.75) |
| Senegal | 5389467 (5231690-5561479) | 67612.5 (65538.05-69827.24) | 6891116 (6365214-7340280) | 41722.15 (38871.84-44252.45) | -1.77 (-1.93--1.6) |
| Togo | 2237181 (2101794-2360817) | 57068.9 (54007.44-60078.78) | 3871723 (3631330-4117836) | 44081.02 (41573.53-46672.53) | -2.38 (-2.48--2.28) |
| Lesotho | 828914 (789492-871079) | 51417.27 (49262.82-53603.67) | 559658 (521104-607406) | 28888.85 (27062.34-31204.15) | -2.19 (-2.27--2.12) |
| Mozambique | 9560282 (9181896-9930821) | 71001.9 (68744.34-73387.02) | 14692934 (13756572-15758624) | 47193.93 (44807.65-49824.86) | -1.72 (-1.83--1.62) |
| Botswana | 568207 (527761-612173) | 39527.35 (37121.53-42221.14) | 563873 (509014-627106) | 23280.96 (21099.36-25834.52) | -1.52 (-1.57--1.46) |
| Zimbabwe | 4679272 (4401763-5013119) | 42622.55 (40317.47-45197.36) | 5812997 (5409344-6272143) | 35564.11 (33325.65-37957.05) | -1 (-1.1--0.89) |
| Seychelles | 17414 (15924-19482) | 23556.54 (21598.1-26243.46) | 16317 (14725-18447) | 15861.68 (14332.27-17888.94) | -1.46 (-1.5--1.42) |
| Eswatini | 325499 (300601-355019) | 35624.91 (33212.84-38285.2) | 270764 (245624-306366) | 22218.72 (20350.49-24843.78) | -0.61 (-0.65--0.56) |
| Cook Islands | 5658 (5121-6255) | 29211.29 (26642.84-32143.44) | 2933 (2645-3302) | 16945.98 (15255.82-18899.01) | -0.25 (-0.39--0.1) |
| United Republic of Tanzania | 14486870 (13448743-15430139) | 54617.39 (51513.69-57749.94) | 21063837 (18578636-23840394) | 35040.92 (31448.6-38916.98) | -1.11 (-1.12--1.09) |
| Bermuda | 9401 (8481-10641) | 16252.12 (14563.11-18556.65) | 5981 (5390-6737) | 10365.73 (9349.45-11674.76) | -2.14 (-2.32--1.96) |
| Benin | 3196103 (3064429-3336111) | 61926.97 (59488.11-64379.34) | 6207006 (5775018-6679962) | 43754.88 (41009.58-46583.59) | -1.52 (-1.54--1.5) |
| Somalia | 7507799 (7417150-7592404) | 94819.35 (93963.3-95649.06) | 19236525 (18810279-19622002) | 89650.83 (88127.52-90983.15) | -1.04 (-1.08--1) |
| Saint Kitts and Nevis | 11612 (10460-13067) | 27977.59 (25463.31-31016.11) | 10370 (9324-11808) | 18559.76 (16698.41-21297.8) | -1.17 (-1.19--1.15) |
| South Africa | 12139106 (11401218-12899654) | 30320.67 (28615.66-32148.81) | 11998663 (11365261-12681815) | 21347.09 (20225.75-22531.68) | -0.99 (-1.01--0.97) |
| Mali | 6831736 (6634057-7028879) | 75926.6 (74091.74-77902.56) | 13980887 (13263576-14701922) | 53999.93 (51376.11-56797.73) | -0.66 (-0.72--0.59) |
| Sudan | 9618841 (9018597-10235563) | 45883.73 (43127.15-48612.73) | 11898022 (10913412-13024181) | 26829.56 (24752.86-29345.83) | -1.16 (-1.21--1.11) |
| Cabo Verde | 177637 (165076-191000) | 47640.53 (44258.39-50721) | 142374 (128032-159516) | 25793.24 (23300.24-28907.95) | -1.13 (-1.17--1.09) |
| Chad | 4873788 (4729902-5012234) | 77456.44 (75268.18-79752.52) | 10585291 (9945449-11249233) | 55414.77 (52181.47-58737.34) | -2.06 (-2.18--1.95) |
| Burkina Faso | 7198490 (6941710-7421188) | 71659.02 (69229.43-73830.53) | 11061247 (10353316-11746385) | 45369.73 (42467.33-48071.45) | -1.2 (-1.23--1.17) |
| Gambia | 682936 (656028-708668) | 66261.66 (63789.91-68728.04) | 1166715 (1084710-1241274) | 46468.45 (43519.81-49143.49) | -1.32 (-1.37--1.27) |
| Cte d'Ivoire | 6423396 (6065990-6797697) | 47955.14 (45468.19-50496.11) | 10020328 (9098774-10851280) | 33812.89 (30810.91-36539.16) | -1.32 (-1.36--1.28) |
| Guinea | 4138491 (3973569-4311661) | 66329.53 (63769.22-68909.97) | 5921638 (5503703-6339763) | 41778.69 (39181.34-44463.35) | -1.27 (-1.33--1.21) |
| Sao Tome and Principe | 69788 (62600-74946) | 53661.88 (48609.91-57546.36) | 72858 (66325-80427) | 32482.35 (29841.59-35460.59) | -1.64 (-1.73--1.54) |
| American Samoa | 13655 (12514-14963) | 27202.01 (25168.75-29694.8) | 10553 (9596-11710) | 22057.39 (20166.1-24375.86) | -1.36 (-1.41--1.31) |
| Liberia | 1392407 (1301970-1486483) | 53900.2 (50608.67-56907.32) | 2142582 (1958455-2347270) | 38161.36 (35380.92-41269.81) | -1.35 (-1.56--1.15) |
| San Marino | 1324 (1203-1491) | 5784.54 (5220.01-6398.3) | 1774 (1580-1994) | 4979.61 (4490.38-5593.38) | -1.47 (-1.59--1.36) |
| Tokelau | 597 (549-647) | 36797.94 (34191.83-39744.54) | 323 (295-355) | 24251.57 (22182.73-26657.58) | -0.77 (-0.85--0.69) |
| Tuvalu | 4811 (4497-5140) | 48456.64 (45295.37-51720.53) | 3924 (3582-4291) | 31619.47 (28941.86-34569.62) | -0.72 (-0.79--0.65) |
| United States Virgin Islands | 21315 (19208-24427) | 19898.29 (17944.7-22695.56) | 11542 (10209-13231) | 15082 (13295.79-17772.57) | -1.49 (-1.58--1.39) |
| Ghana | 9449274 (8963314-9900318) | 60568.94 (57562.11-63236.89) | 14563058 (13401717-15754292) | 41284.71 (38445.11-44283.16) | -1.72 (-1.78--1.66) |
| Mauritania | 1191464 (1135752-1246127) | 54374.74 (52056.58-56926.58) | 1622255 (1479608-1795265) | 35218.84 (32632.46-38329.96) | -1.04 (-1.06--1.01) |
| Niger | 7023695 (6864762-7147066) | 84719.38 (82952.16-86178.61) | 16801641 (15911153-17695744) | 62976.18 (59996.06-65782.07) | -1.38 (-1.44--1.31) |
| Sierra Leone | 2637057 (2503019-2745817) | 60348.9 (57691.43-62775.05) | 3637007 (3329965-3945104) | 39143.46 (36066.24-42076.07) | -0.55 (-0.78--0.32) |
| Greenland | 4366 (3715-5275) | 7830.69 (6815.6-9464.15) | 3075 (2783-3447) | 5395.06 (4888.3-5983.53) | -0.82 (-0.95--0.68) |
| Nigeria | 36652682 (34600147-39093230) | 38202.42 (36094.66-40536.58) | 76921206 (71726075-82817227) | 31651.73 (29818.58-33650.41) | -1.33 (-1.45--1.21) |
| Nauru | 3816 (3513-4162) | 35750.17 (33052.75-38711.44) | 3303 (2994-3678) | 29686.74 (27083.35-32685.48) | -0.23 (-0.33--0.13) |
| Puerto Rico | 635547 (572063-722649) | 17867.54 (16108.93-20266.11) | 370145 (332292-422066) | 12047.77 (10782.01-13993.26) | -0.42 (-0.46--0.37) |
| Guam | 30139 (27006-34306) | 21569.65 (19539.4-24308.19) | 27455 (24841-31180) | 17167.05 (15568.5-19462.72) | -0.96 (-1.02--0.9) |
| Monaco | 1487 (1325-1705) | 4832.68 (4297.82-5556.84) | 1719 (1520-1969) | 4114.87 (3647.46-4721.04) | -0.91 (-1--0.83) |
| Northern Mariana Islands | 9812 (8797-11036) | 21587.99 (19491.97-24168.3) | 8672 (7736-9853) | 18772.56 (16811.44-21004.46) | -1.36 (-1.39--1.34) |
| Palau | 4075 (3689-4550) | 26986.69 (24621.7-29908.48) | 3626 (3230-4135) | 20931.34 (18977.32-23450.81) | -1.25 (-1.31--1.19) |
| South Sudan | 3781586 (3548051-4010254) | 64111.86 (60710.38-67535.63) | 4818122 (4462876-5186506) | 49749.97 (46491.92-53076.87) | -0.34 (-0.43--0.24) |
| Niue | 717 (659-787) | 30863.66 (28399.05-33577.08) | 366 (330-408) | 22341.91 (20265.96-24742) | -1.72 (-1.8--1.64) |

## Table S4：Deaths of Nutritional Deficiencies in cases and age-standardized rates for both sexes combined in 1990 and 2021, with percentage change between 1990 and 2021 by GBD 204 nations.

| Deaths | | | | | |
| --- | --- | --- | --- | --- | --- |
| nations | **1990** | | **2021** | | **1990-2021 EAPC** |
|  | **All-ages cases(both sexs)** | **Age-standardized rates per 100,000 people** | **All-ages cases(both sexs)** | **Age-standardized rates per 100,000 people** |  |
|  | **n (95% UI)** | **n (95% UI)** | **n (95% UI)** | **n (95% UI)** | **n (95% UI)** |
| Slovenia | 2 (2-2) | 0.09 (0.08-0.09) | 10 (8-12) | 0.21 (0.17-0.25) | -5.33 (-5.58--5.08) |
| North Macedonia | 3 (2-4) | 0.17 (0.12-0.22) | 3 (2-4) | 0.13 (0.09-0.18) | -0.16 (-0.44-0.13) |
| Australia | 72 (65-76) | 0.41 (0.37-0.44) | 148 (123-163) | 0.28 (0.23-0.3) | -0.13 (-0.29-0.03) |
| Lithuania | 26 (24-28) | 0.66 (0.61-0.71) | 16 (14-18) | 0.35 (0.31-0.4) | -5.98 (-6.71--5.25) |
| New Zealand | 13 (11-14) | 0.35 (0.31-0.38) | 17 (14-19) | 0.18 (0.15-0.2) | 0.35 (0.08-0.63) |
| Japan | 606 (553-634) | 0.42 (0.38-0.44) | 2040 (1669-2253) | 0.46 (0.41-0.49) | -4.25 (-4.92--3.56) |
| Andorra | 0 (0-0) | 0.02 (0.02-0.03) | 0 (0-0) | 0.01 (0.01-0.02) | -1.94 (-2.2--1.69) |
| Taiwan (Province of China) | 279 (260-296) | 2.91 (2.62-3.14) | 277 (234-310) | 0.63 (0.54-0.69) | -0.59 (-1.12--0.05) |
| Democratic People's Republic of Korea | 635 (418-984) | 4.61 (3.1-6.77) | 420 (295-589) | 1.87 (1.29-2.65) | -3.31 (-3.38--3.25) |
| China | 37247 (32236-42771) | 5.53 (4.85-6.19) | 15756 (13053-18627) | 1.14 (0.94-1.34) | -6.36 (-6.82--5.89) |
| Argentina | 1514 (1438-1591) | 4.89 (4.64-5.15) | 978 (859-1058) | 1.73 (1.53-1.88) | 0.23 (0-0.45) |
| Malta | 0 (0-0) | 0.11 (0.1-0.12) | 1 (1-1) | 0.1 (0.08-0.12) | -1.21 (-1.54--0.87) |
| Israel | 14 (12-15) | 0.37 (0.32-0.4) | 56 (44-64) | 0.37 (0.29-0.42) | -12.08 (-18.22--5.48) |
| Fiji | 14 (11-18) | 4.85 (3.74-6.22) | 15 (11-21) | 3.24 (2.43-4.31) | -3.6 (-3.73--3.46) |
| Norway | 13 (11-14) | 0.17 (0.15-0.18) | 199 (158-221) | 1.52 (1.23-1.68) | 0.41 (0.16-0.66) |
| Canada | 174 (153-189) | 0.57 (0.5-0.62) | 493 (419-545) | 0.6 (0.52-0.65) | -5 (-5.22--4.78) |
| Belarus | 75 (68-84) | 0.7 (0.63-0.78) | 67 (54-81) | 0.5 (0.41-0.6) | -4.48 (-4.78--4.17) |
| Papua New Guinea | 135 (99-180) | 7.23 (5.51-9.37) | 211 (153-288) | 4.6 (3.44-6.09) | -3.45 (-3.79--3.11) |
| United Kingdom | 170 (155-177) | 0.2 (0.18-0.21) | 115 (99-123) | 0.08 (0.07-0.09) | -2.61 (-2.93--2.29) |
| Armenia | 72 (64-80) | 2.19 (1.96-2.44) | 9 (8-10) | 0.25 (0.21-0.28) | -5 (-5.53--4.45) |
| Lao People's Democratic Republic | 1161 (793-1938) | 29.75 (21.9-42.52) | 228 (167-306) | 5.94 (4.41-7.74) | -1.39 (-1.44--1.34) |
| Maldives | 41 (27-65) | 25.72 (17.87-37.58) | 6 (5-7) | 2.26 (1.79-2.82) | -2.65 (-3.4--1.9) |
| Timor-Leste | 361 (204-691) | 45.81 (27.18-83.55) | 91 (65-135) | 11.74 (8.26-17.37) | -3.31 (-4.57--2.04) |
| Viet Nam | 3122 (2089-4650) | 6.3 (4.32-8.99) | 1208 (963-1489) | 1.56 (1.24-1.94) | -4.7 (-5.24--4.15) |
| Micronesia (Federated States of) | 6 (4-8) | 10.21 (6.82-13.4) | 2 (1-3) | 4.89 (3.63-6.24) | -5.42 (-5.56--5.29) |
| Turkmenistan | 45 (38-54) | 1.04 (0.9-1.23) | 13 (10-16) | 0.27 (0.21-0.34) | -3.69 (-3.96--3.42) |
| Chile | 330 (311-348) | 3.24 (3.04-3.43) | 418 (364-455) | 1.66 (1.46-1.8) | -6.69 (-7.3--6.07) |
| Switzerland | 92 (80-100) | 0.83 (0.72-0.9) | 135 (105-155) | 0.51 (0.41-0.58) | -3.77 (-4.29--3.25) |
| Cambodia | 3356 (2108-5515) | 39.07 (27.29-57.61) | 553 (419-703) | 6.66 (5.12-8.31) | -4.46 (-4.72--4.21) |
| Indonesia | 21788 (18105-25764) | 24.56 (20.11-29.32) | 19803 (16200-23043) | 15.06 (12.12-17.48) | -1.47 (-1.72--1.22) |
| Malaysia | 219 (185-267) | 2.18 (1.81-2.63) | 330 (272-391) | 1.58 (1.27-1.89) | -3.86 (-4.03--3.7) |
| Myanmar | 4348 (3117-6042) | 17.85 (13.37-23.33) | 1347 (1037-1708) | 3.64 (2.8-4.65) | -3.73 (-3.98--3.48) |
| Samoa | 5 (4-7) | 5.76 (4.51-7.25) | 4 (3-5) | 3.4 (2.56-4.3) | -0.46 (-0.65--0.26) |
| Philippines | 4486 (3937-5320) | 12.69 (11.39-13.97) | 3674 (3212-4167) | 6 (5.15-6.85) | -2.16 (-2.56--1.76) |
| Thailand | 1755 (1362-2165) | 6.61 (5.07-8.42) | 1516 (1149-1876) | 1.51 (1.16-1.85) | -8.43 (-9.52--7.33) |
| Kiribati | 23 (17-30) | 37.76 (29.44-46.92) | 13 (10-17) | 22.86 (17.66-28.68) | -2.38 (-2.89--1.87) |
| Georgia | 24 (21-27) | 0.45 (0.4-0.51) | 3 (3-4) | 0.07 (0.06-0.08) | 3.29 (2.65-3.93) |
| Albania | 30 (21-43) | 1.01 (0.7-1.44) | 18 (11-28) | 0.54 (0.33-0.86) | -1.47 (-1.78--1.16) |
| Slovakia | 8 (7-10) | 0.15 (0.12-0.19) | 11 (8-14) | 0.13 (0.1-0.17) | -3.07 (-3.62--2.52) |
| Solomon Islands | 19 (13-26) | 9.82 (7.28-13.04) | 15 (10-20) | 4.96 (3.43-6.6) | -3.79 (-3.98--3.61) |
| Kyrgyzstan | 35 (31-40) | 0.79 (0.71-0.88) | 6 (5-7) | 0.1 (0.08-0.12) | -4.95 (-5.19--4.7) |
| Kazakhstan | 143 (129-157) | 0.92 (0.83-1.01) | 95 (85-106) | 0.59 (0.52-0.65) | -4.5 (-4.98--4.02) |
| Vanuatu | 7 (5-10) | 10.23 (7.3-13.86) | 8 (6-12) | 6.55 (4.49-9.12) | -7.35 (-7.82--6.88) |
| Tajikistan | 108 (85-137) | 1.62 (1.31-2.01) | 50 (31-76) | 0.51 (0.33-0.73) | -1.42 (-1.79--1.04) |
| Tonga | 4 (3-6) | 7 (5.44-8.68) | 4 (3-5) | 5.08 (3.78-6.57) | -5.79 (-6.21--5.37) |
| Antigua and Barbuda | 4 (3-4) | 6.13 (5.55-6.64) | 3 (3-3) | 3.72 (3.42-3.99) | -1.63 (-1.81--1.44) |
| Marshall Islands | 2 (1-2) | 9.45 (6.89-12.33) | 1 (1-1) | 4.91 (3.69-6.46) | -1.48 (-1.89--1.07) |
| Mongolia | 14 (8-23) | 0.56 (0.34-0.9) | 4 (3-6) | 0.14 (0.1-0.19) | -1.57 (-1.81--1.34) |
| Sri Lanka | 422 (365-483) | 5.16 (4.42-5.93) | 195 (138-250) | 0.95 (0.68-1.22) | -5.45 (-6.01--4.89) |
| Bosnia and Herzegovina | 2 (1-2) | 0.04 (0.03-0.05) | 7 (5-9) | 0.12 (0.09-0.15) | -5.38 (-5.82--4.94) |
| Bulgaria | 6 (5-6) | 0.07 (0.06-0.08) | 26 (22-30) | 0.22 (0.19-0.25) | 2.89 (2.28-3.5) |
| Estonia | 14 (13-16) | 0.86 (0.8-0.93) | 8 (7-9) | 0.35 (0.3-0.4) | -6.16 (-6.51--5.82) |
| Serbia | 15 (12-18) | 0.18 (0.14-0.22) | 22 (17-28) | 0.14 (0.11-0.18) | 3.31 (2.61-4.01) |
| Azerbaijan | 165 (130-208) | 2.18 (1.75-2.71) | 38 (29-49) | 0.41 (0.31-0.55) | -1.96 (-2.38--1.53) |
| Guyana | 157 (135-182) | 25.39 (22.87-27.9) | 46 (36-59) | 7.99 (6.25-10.13) | -4.88 (-5.21--4.55) |
| Haiti | 1990 (1482-2702) | 21.98 (16.89-29.01) | 860 (614-1233) | 6.86 (5.11-9.41) | 0 (-0.35-0.36) |
| Grenada | 5 (5-6) | 5.91 (5.32-6.59) | 4 (3-4) | 4 (3.5-4.45) | -1.7 (-1.75--1.66) |
| Jamaica | 176 (161-193) | 7.94 (7.23-8.61) | 79 (63-98) | 2.38 (1.88-2.99) | -2.16 (-2.32--2) |
| Uzbekistan | 295 (254-344) | 1.24 (1.1-1.42) | 58 (48-70) | 0.18 (0.15-0.22) | 0.21 (-0.01-0.43) |
| Poland | 40 (38-42) | 0.1 (0.1-0.11) | 234 (210-255) | 0.33 (0.3-0.36) | -7.37 (-7.93--6.8) |
| Saint Vincent and the Grenadines | 13 (11-14) | 15.68 (14.28-17.03) | 11 (9-12) | 9.36 (8.18-10.61) | -1.5 (-1.95--1.04) |
| Czechia | 31 (28-34) | 0.26 (0.24-0.29) | 205 (173-238) | 0.96 (0.81-1.13) | -7.05 (-7.47--6.64) |
| Brunei Darussalam | 1 (1-1) | 1 (0.82-1.21) | 1 (1-1) | 0.52 (0.42-0.63) | -3.08 (-3.3--2.86) |
| Hungary | 15 (14-16) | 0.12 (0.12-0.13) | 60 (52-68) | 0.33 (0.28-0.38) | -4.31 (-4.95--3.68) |
| Croatia | 2 (2-2) | 0.04 (0.03-0.04) | 8 (7-9) | 0.1 (0.09-0.11) | -1.91 (-2.84--0.96) |
| Ukraine | 472 (440-507) | 0.92 (0.85-1) | 69 (52-89) | 0.13 (0.1-0.17) | -1.15 (-1.4--0.9) |
| Latvia | 26 (25-29) | 0.9 (0.84-0.98) | 16 (14-18) | 0.51 (0.44-0.57) | -4.77 (-5.06--4.48) |
| Montenegro | 1 (1-1) | 0.12 (0.1-0.16) | 1 (1-1) | 0.12 (0.09-0.15) | -0.51 (-0.81--0.21) |
| Romania | 38 (35-41) | 0.18 (0.17-0.2) | 132 (114-149) | 0.4 (0.35-0.46) | -3.65 (-3.86--3.44) |
| Singapore | 4 (4-5) | 0.24 (0.22-0.26) | 6 (5-7) | 0.08 (0.07-0.09) | -5.29 (-6.66--3.89) |
| Republic of Moldova | 19 (18-21) | 0.46 (0.42-0.5) | 11 (10-13) | 0.25 (0.21-0.29) | -4.79 (-5.04--4.53) |
| Russian Federation | 473 (464-480) | 0.29 (0.29-0.3) | 690 (637-742) | 0.32 (0.3-0.35) | 1.31 (1-1.62) |
| Iceland | 1 (1-1) | 0.23 (0.2-0.25) | 3 (2-3) | 0.36 (0.29-0.42) | -1.76 (-1.99--1.54) |
| Finland | 12 (10-13) | 0.18 (0.16-0.19) | 33 (27-37) | 0.19 (0.16-0.21) | -2.75 (-3.09--2.41) |
| France | 2402 (2104-2633) | 2.87 (2.51-3.15) | 5206 (4077-6008) | 2.29 (1.84-2.6) | -4.94 (-5.57--4.3) |
| Belgium | 73 (65-79) | 0.51 (0.45-0.55) | 248 (194-283) | 0.76 (0.61-0.86) | -2 (-2.95--1.04) |
| Netherlands | 89 (78-96) | 0.46 (0.4-0.5) | 216 (176-241) | 0.53 (0.43-0.59) | -4.31 (-4.48--4.15) |
| Austria | 10 (9-11) | 0.09 (0.08-0.1) | 29 (24-33) | 0.12 (0.1-0.13) | -2.47 (-2.65--2.28) |
| Italy | 73 (66-77) | 0.09 (0.08-0.1) | 683 (546-759) | 0.34 (0.28-0.37) | -1.22 (-1.58--0.86) |
| Denmark | 96 (85-103) | 1.13 (1-1.22) | 104 (88-115) | 0.73 (0.63-0.81) | -4.49 (-5.46--3.51) |
| Cyprus | 3 (2-3) | 0.75 (0.59-0.93) | 5 (4-6) | 0.37 (0.29-0.47) | -2.53 (-3.25--1.81) |
| Peru | 3270 (2717-3906) | 17.54 (15.02-20.46) | 1596 (1205-2035) | 4.71 (3.57-6.01) | -6.46 (-6.78--6.14) |
| Republic of Korea | 463 (412-520) | 2.76 (2.34-3.2) | 396 (321-479) | 0.47 (0.38-0.57) | -2.17 (-2.67--1.66) |
| Germany | 193 (173-208) | 0.15 (0.14-0.16) | 744 (601-840) | 0.3 (0.25-0.33) | -0.48 (-1.6-0.67) |
| Greece | 6 (5-6) | 0.05 (0.04-0.05) | 20 (17-22) | 0.06 (0.05-0.06) | -5.36 (-5.5--5.23) |
| Algeria | 352 (229-563) | 1.7 (1.22-2.42) | 124 (96-154) | 0.51 (0.39-0.64) | -2.25 (-2.39--2.11) |
| Ireland | 12 (11-13) | 0.35 (0.31-0.38) | 18 (15-21) | 0.21 (0.17-0.24) | 0.14 (-0.07-0.35) |
| Luxembourg | 4 (3-4) | 0.82 (0.75-0.87) | 11 (9-12) | 0.82 (0.69-0.91) | -0.58 (-1.03--0.13) |
| Uruguay | 99 (93-105) | 2.99 (2.79-3.16) | 95 (84-103) | 1.5 (1.35-1.63) | 2.23 (1.57-2.89) |
| Spain | 192 (168-208) | 0.4 (0.35-0.43) | 466 (369-530) | 0.33 (0.27-0.37) | -1.41 (-2.5--0.3) |
| Portugal | 75 (69-80) | 0.72 (0.64-0.77) | 185 (150-206) | 0.56 (0.47-0.62) | 4.24 (3.75-4.75) |
| Suriname | 25 (20-30) | 7.63 (6.33-9.12) | 11 (8-14) | 2.06 (1.49-2.67) | -1.65 (-1.95--1.36) |
| Venezuela (Bolivarian Republic of) | 1164 (1099-1230) | 8.3 (7.76-8.78) | 755 (585-955) | 2.88 (2.25-3.65) | -3.23 (-3.4--3.06) |
| Belize | 23 (20-25) | 12.08 (11.09-13.1) | 15 (13-17) | 5.2 (4.56-5.88) | 2.12 (1.27-2.98) |
| Sweden | 50 (43-54) | 0.31 (0.26-0.33) | 136 (109-153) | 0.46 (0.38-0.52) | -4.41 (-4.62--4.2) |
| Bahamas | 8 (7-8) | 4.32 (3.92-4.68) | 6 (5-8) | 1.92 (1.59-2.36) | -5.06 (-5.27--4.85) |
| Barbados | 12 (11-13) | 4.41 (4.05-4.79) | 6 (5-7) | 1.22 (0.98-1.49) | -6.67 (-7.03--6.31) |
| Dominica | 3 (3-4) | 5.58 (4.91-6.42) | 2 (1-2) | 2.76 (2.13-3.63) | -4.38 (-4.54--4.22) |
| Tunisia | 58 (41-77) | 0.82 (0.61-1.03) | 32 (23-43) | 0.31 (0.22-0.41) | -3.28 (-3.74--2.83) |
| Saudi Arabia | 335 (227-510) | 3.13 (2.27-4.23) | 93 (72-120) | 0.78 (0.61-0.99) | -1.16 (-1.31--1.02) |
| United States of America | 2194 (1928-2332) | 0.66 (0.58-0.7) | 13008 (10879-14175) | 1.99 (1.69-2.15) | -3.52 (-3.68--3.37) |
| Bolivia (Plurinational State of) | 2312 (1616-2980) | 40.35 (30.52-49.98) | 797 (602-1022) | 10.27 (7.78-12.96) | -5.73 (-6--5.46) |
| Dominican Republic | 1212 (1006-1418) | 17.74 (15.21-20.45) | 537 (409-698) | 5.47 (4.16-7.11) | -5.64 (-5.82--5.46) |
| Colombia | 2257 (2057-2454) | 9.56 (8.79-10.15) | 1510 (1227-1853) | 3.02 (2.42-3.77) | -7.01 (-7.64--6.38) |
| Trkiye | 1633 (1263-2155) | 3.06 (2.47-3.87) | 1211 (984-1489) | 1.64 (1.33-2.01) | -4.15 (-5.38--2.9) |
| Saint Lucia | 10 (9-11) | 10.78 (10.04-11.55) | 7 (6-9) | 3.7 (3.08-4.44) | -4.11 (-4.24--3.98) |
| Cuba | 79 (74-84) | 0.87 (0.81-0.92) | 94 (81-106) | 0.49 (0.42-0.56) | -2.64 (-2.96--2.32) |
| Afghanistan | 1162 (790-1675) | 7.93 (5.82-10.67) | 848 (598-1154) | 2.29 (1.71-2.97) | -2.17 (-3.11--1.23) |
| Bangladesh | 63105 (51596-74352) | 52.66 (43.26-62.52) | 3435 (2609-4265) | 2.88 (2.23-3.56) | -0.84 (-0.94--0.74) |
| Brazil | 12614 (11669-13629) | 11.25 (10.46-11.99) | 6022 (5261-6480) | 2.6 (2.27-2.81) | 2.74 (1.85-3.63) |
| El Salvador | 496 (436-562) | 11.27 (9.94-12.53) | 314 (248-392) | 4.47 (3.55-5.63) | -1.19 (-1.6--0.79) |
| Trinidad and Tobago | 51 (47-55) | 6.69 (6.19-7.2) | 21 (16-26) | 1.27 (1-1.61) | -3.85 (-4.88--2.82) |
| Ecuador | 1317 (1239-1398) | 21.06 (19.65-22.19) | 592 (501-690) | 4.21 (3.6-4.85) | -2.32 (-2.69--1.95) |
| Costa Rica | 40 (37-43) | 2.15 (1.95-2.3) | 29 (24-32) | 0.51 (0.43-0.57) | -6.1 (-6.59--5.61) |
| Guatemala | 5209 (4974-5479) | 159.65 (151.32-166.78) | 1147 (989-1336) | 12.08 (10.59-13.8) | -2.64 (-3.67--1.61) |
| India | 132756 (103099-168324) | 15.41 (12.19-18.95) | 24072 (19936-29354) | 2.28 (1.89-2.77) | -5.92 (-6.34--5.5) |
| Bahrain | 4 (3-5) | 2.68 (2.29-3.12) | 4 (3-5) | 1.16 (0.94-1.42) | -4.27 (-4.61--3.92) |
| Mexico | 15513 (14664-16418) | 33.74 (32.11-35.02) | 7028 (6329-7790) | 6.51 (5.83-7.21) | -4.38 (-4.72--4.03) |
| Jordan | 17 (14-20) | 0.71 (0.57-0.86) | 16 (13-19) | 0.29 (0.23-0.35) | -2.2 (-2.42--1.97) |
| Palestine | 16 (11-21) | 1.03 (0.79-1.34) | 10 (8-12) | 0.48 (0.39-0.57) | -2.3 (-2.41--2.18) |
| Qatar | 1 (1-1) | 0.82 (0.68-1) | 3 (2-3) | 0.7 (0.54-0.87) | 0.58 (-0.13-1.29) |
| Syrian Arab Republic | 387 (294-493) | 3.42 (2.75-4.14) | 110 (85-137) | 1.42 (1.11-1.74) | -3.44 (-3.9--2.98) |
| Nicaragua | 377 (316-466) | 11.08 (9.61-12.88) | 136 (108-170) | 3.06 (2.43-3.81) | 1.45 (0.96-1.95) |
| Iran (Islamic Republic of) | 1036 (856-1349) | 1.78 (1.51-2.15) | 201 (180-223) | 0.31 (0.28-0.35) | -3.01 (-3.27--2.75) |
| Honduras | 341 (286-409) | 10.93 (8.97-13.29) | 332 (260-423) | 6.65 (5.33-8.31) | -3.86 (-4.05--3.66) |
| Congo | 296 (218-379) | 15.2 (11.33-18.37) | 240 (188-306) | 8.44 (6.71-10.63) | 4.55 (3.93-5.18) |
| Paraguay | 163 (135-198) | 4.61 (3.94-5.42) | 202 (155-259) | 3.64 (2.83-4.68) | 1.43 (0.93-1.94) |
| Egypt | 1452 (1169-1795) | 5.15 (4.17-6.09) | 367 (278-476) | 0.63 (0.49-0.78) | 0.19 (-0.12-0.5) |
| Panama | 171 (156-185) | 10 (9.05-10.74) | 130 (104-155) | 2.93 (2.35-3.52) | -7.85 (-8.51--7.19) |
| Comoros | 185 (128-242) | 42.75 (32.94-53.28) | 54 (41-72) | 10.44 (7.97-13.61) | 0.87 (0.32-1.43) |
| Kuwait | 2 (2-3) | 0.28 (0.25-0.31) | 2 (2-2) | 0.08 (0.07-0.1) | -4.48 (-4.76--4.19) |
| Iraq | 452 (337-630) | 2.29 (1.82-2.97) | 172 (135-215) | 0.85 (0.66-1.05) | -1.39 (-1.5--1.28) |
| United Arab Emirates | 7 (5-9) | 1.04 (0.8-1.3) | 8 (6-10) | 0.62 (0.49-0.79) | -4.8 (-5.1--4.51) |
| Angola | 9969 (7139-14556) | 76.94 (56.85-104.89) | 2801 (1947-3679) | 12.38 (9.5-15.53) | -2.84 (-4.06--1.59) |
| Lebanon | 13 (10-16) | 0.6 (0.47-0.75) | 18 (15-22) | 0.27 (0.22-0.33) | -2.82 (-3.58--2.05) |
| Morocco | 558 (401-777) | 2.2 (1.67-2.88) | 195 (144-262) | 0.75 (0.55-0.99) | 10.64 (7.83-13.53) |
| Nepal | 8351 (5949-11712) | 43.12 (31.48-57.94) | 1338 (1073-1646) | 6.67 (5.41-8.37) | -5.09 (-5.55--4.62) |
| Libya | 23 (17-32) | 0.67 (0.48-0.88) | 22 (17-28) | 0.51 (0.39-0.66) | -1.03 (-1.22--0.84) |
| Oman | 91 (66-133) | 7.15 (5.37-9.35) | 30 (24-38) | 2.16 (1.69-2.77) | -4.43 (-4.62--4.24) |
| Yemen | 2466 (1581-3696) | 13.05 (8.7-18.27) | 1126 (721-1605) | 4.88 (3.22-6.94) | -0.19 (-0.38--0.01) |
| Burundi | 5167 (3066-8905) | 86.76 (57.34-139.7) | 1479 (915-2230) | 18.15 (13.34-24.09) | -6.65 (-7.12--6.18) |
| Malawi | 6006 (4282-8366) | 48.53 (37.72-62.28) | 1727 (1244-2249) | 14.13 (11.03-17.56) | -1.18 (-1.4--0.96) |
| Bhutan | 42 (20-71) | 6.37 (2.9-10) | 4 (2-6) | 0.69 (0.35-1.08) | 2.39 (1.71-3.09) |
| Pakistan | 10715 (8355-13108) | 9.38 (7.52-11.12) | 6380 (4678-8543) | 3.66 (2.71-4.75) | -1.68 (-2.71--0.64) |
| Democratic Republic of the Congo | 12686 (8369-19002) | 26.72 (20.57-36.25) | 5002 (3022-7647) | 8.78 (5.5-12.71) | 0.88 (0.31-1.45) |
| Central African Republic | 853 (553-1326) | 28.78 (19.93-40.52) | 1070 (762-1469) | 26.06 (20.64-33.02) | -4.8 (-5.25--4.33) |
| Equatorial Guinea | 96 (66-134) | 21.14 (15.38-28.48) | 40 (24-60) | 5.38 (3.72-7.43) | -6.35 (-6.54--6.16) |
| Gabon | 87 (65-117) | 10.91 (7.98-14.66) | 60 (42-85) | 5.86 (4.19-8.04) | -0.87 (-1.76-0.04) |
| Rwanda | 3727 (2617-5001) | 48.18 (38.22-60.77) | 830 (635-1050) | 10.24 (8.07-12.87) | -5.77 (-6.05--5.48) |
| Uganda | 6802 (4659-9494) | 30.49 (23.71-38.33) | 3249 (2072-4683) | 10.3 (7.76-13.06) | -3.05 (-3.75--2.34) |
| Djibouti | 123 (85-174) | 38.62 (27.84-55.26) | 93 (61-132) | 13.87 (9.77-19.46) | -0.91 (-1.24--0.59) |
| Eritrea | 3810 (2646-5172) | 132.72 (96.94-179.06) | 877 (629-1206) | 22.37 (17.47-28.64) | -2.73 (-3.08--2.37) |
| Kenya | 5909 (4588-7538) | 22.94 (19.3-27.27) | 3326 (2753-3972) | 10.51 (8.42-12.92) | -3.87 (-4.08--3.66) |
| Madagascar | 11577 (9532-13587) | 91.61 (79.9-103.46) | 4766 (3478-6194) | 27.49 (21.23-35.01) | -0.13 (-0.48-0.21) |
| Ethiopia | 38443 (28617-52500) | 80.67 (62.31-104.08) | 9627 (7601-11847) | 12.85 (10.67-15.44) | -4.91 (-5.05--4.76) |
| Zambia | 3937 (2787-5876) | 38.88 (29.79-52.45) | 1295 (851-1807) | 10.42 (7.46-13.83) | -9.67 (-10.06--9.27) |
| Mauritius | 38 (36-39) | 6.14 (5.74-6.5) | 23 (21-25) | 1.56 (1.4-1.68) | -4.69 (-5.03--4.35) |
| Namibia | 243 (184-312) | 17.45 (14.01-21.6) | 137 (96-193) | 6.97 (5.09-9.42) | -5.71 (-6--5.42) |
| Cameroon | 1182 (820-1609) | 10.24 (7.83-13.05) | 858 (585-1181) | 4.23 (3.05-5.62) | -0.54 (-0.77--0.31) |
| Guinea-Bissau | 372 (240-576) | 32.32 (23.54-45.21) | 71 (49-103) | 7.08 (5.03-10.06) | -5.06 (-5.24--4.88) |
| Senegal | 1550 (1050-2183) | 14.81 (10.68-19.6) | 308 (186-445) | 2.78 (1.68-3.97) | -2.82 (-3.74--1.88) |
| Togo | 602 (405-836) | 15.7 (12.04-20.09) | 207 (145-288) | 4.66 (3.45-6.31) | -2.92 (-4.25--1.58) |
| Lesotho | 225 (169-294) | 13.64 (10.24-17.48) | 155 (111-213) | 10.26 (7.61-13.62) | -5.37 (-5.74--5) |
| Mozambique | 8741 (5924-12692) | 53.24 (39.29-71.85) | 2882 (2060-3920) | 14.41 (11-18.52) | -2.98 (-3.2--2.76) |
| Botswana | 139 (98-184) | 10.87 (7.98-14.01) | 116 (80-163) | 5.9 (4.28-7.97) | -2.22 (-2.33--2.12) |
| Zimbabwe | 1651 (1266-2090) | 19.19 (16.01-23.02) | 2923 (2102-3809) | 23.8 (18.16-29.68) | 2.84 (2.23-3.46) |
| Seychelles | 3 (3-3) | 5.22 (4.54-5.98) | 3 (2-3) | 2.86 (2.44-3.31) | -0.72 (-1.45-0.01) |
| Eswatini | 110 (78-147) | 11.6 (8.36-14.91) | 64 (43-92) | 6.59 (4.56-9.2) | -3.3 (-3.39--3.21) |
| Cook Islands | 0 (0-0) | 0.52 (0.43-0.64) | 0 (0-0) | 0.25 (0.19-0.31) | -6.29 (-7.11--5.47) |
| United Republic of Tanzania | 13155 (9650-17265) | 41.52 (33.15-50.91) | 4457 (3289-5827) | 9.8 (7.66-12.14) | -1.98 (-2.3--1.67) |
| Bermuda | 1 (1-1) | 1.26 (1.15-1.35) | 1 (1-1) | 0.45 (0.38-0.54) | -2.7 (-3.07--2.33) |
| Benin | 1719 (1067-2640) | 25.73 (17.86-35.88) | 587 (384-844) | 5.49 (4.17-6.99) | -2.87 (-4.03--1.69) |
| Somalia | 5941 (3979-8398) | 78.8 (55.8-105.48) | 4521 (2954-6574) | 32.07 (22.72-45.69) | -1.08 (-1.47--0.69) |
| Saint Kitts and Nevis | 8 (7-9) | 22.13 (20.59-23.7) | 4 (4-5) | 9.38 (8.09-10.86) | -2.49 (-2.71--2.27) |
| South Africa | 5081 (4242-6247) | 12.25 (10.49-14.86) | 2348 (1919-2748) | 5.11 (4.21-5.96) | -5.02 (-5.21--4.83) |
| Mali | 6809 (4622-9655) | 54.8 (41.2-71.66) | 7456 (4979-10354) | 31.04 (24.56-38.58) | -2.76 (-2.89--2.64) |
| Sudan | 4094 (2393-7349) | 14.74 (9.16-24.68) | 680 (418-1072) | 2.1 (1.36-3.21) | 1.12 (0.71-1.52) |
| Cabo Verde | 69 (51-89) | 15.63 (12.25-19.5) | 10 (8-13) | 2.46 (1.96-3.03) | -3.15 (-3.38--2.92) |
| Chad | 2991 (2003-4386) | 36.89 (26.88-51.08) | 1889 (1315-2665) | 12.15 (9.12-15.87) | -3.57 (-3.95--3.19) |
| Burkina Faso | 4531 (3190-6143) | 36.21 (27.53-45.49) | 2872 (1938-4149) | 12.32 (9.36-16.27) | 0.77 (0.54-0.99) |
| Gambia | 264 (187-350) | 23.6 (18.54-28.82) | 118 (88-156) | 7.76 (5.92-9.78) | -8.41 (-8.8--8.02) |
| Cte d'Ivoire | 1843 (1311-2483) | 14.55 (11.02-18.54) | 784 (555-1068) | 4.49 (3.52-5.77) | 4.46 (3.57-5.36) |
| Guinea | 2903 (1979-4095) | 30.38 (21.88-40.76) | 1323 (869-1874) | 9.71 (7.23-12.71) | 0.39 (0.11-0.67) |
| Sao Tome and Principe | 44 (33-56) | 28.34 (22.69-34.12) | 7 (6-10) | 6.92 (5.63-8.6) | -1.34 (-2.28--0.39) |
| American Samoa | 2 (2-3) | 10.37 (8.46-12.58) | 2 (2-3) | 7.29 (5.73-9.32) | -0.74 (-0.89--0.6) |
| Liberia | 1589 (1117-2256) | 45.12 (34.01-59.76) | 316 (207-453) | 9.27 (6.97-12.27) | -5.05 (-5.26--4.84) |
| San Marino | 0 (0-0) | 0.27 (0.21-0.34) | 0 (0-0) | 0.13 (0.08-0.19) | -7.18 (-7.7--6.65) |
| Tokelau | 0 (0-0) | 7.85 (5.97-9.89) | 0 (0-0) | 5.21 (4.04-6.53) | -1.95 (-2.97--0.92) |
| Tuvalu | 1 (1-1) | 12.51 (9.8-15.9) | 0 (0-0) | 4.45 (3.46-5.6) | -1.51 (-1.72--1.3) |
| United States Virgin Islands | 3 (2-4) | 4.48 (3.65-5.35) | 2 (2-3) | 1.67 (1.34-2.13) | -1.75 (-2.12--1.38) |
| Ghana | 4378 (2919-6288) | 30.53 (23.15-39.85) | 1911 (1415-2467) | 10.32 (8.17-12.75) | -0.14 (-0.51-0.23) |
| Mauritania | 483 (309-766) | 26.01 (17.69-40.2) | 98 (64-158) | 3.99 (2.63-6.39) | -1.47 (-4.55-1.72) |
| Niger | 5718 (3559-9374) | 40 (27.21-61.1) | 1614 (1038-2329) | 5.54 (4.04-7.38) | -3.2 (-3.46--2.94) |
| Sierra Leone | 2773 (1908-3772) | 43.72 (32.3-56.71) | 3430 (2280-4952) | 39.82 (29.4-52.88) | -3.51 (-3.77--3.26) |
| Greenland | 0 (0-0) | 1.72 (1.41-2.04) | 1 (0-1) | 1.21 (0.97-1.54) | -3.35 (-3.92--2.77) |
| Nigeria | 10369 (7713-15186) | 7.77 (6.03-10.87) | 4242 (2765-5830) | 1.68 (1.23-2.15) | -6.09 (-6.33--5.85) |
| Nauru | 0 (0-1) | 7.53 (5.86-9.82) | 0 (0-0) | 5.41 (4.05-7.23) | -1.05 (-1.17--0.92) |
| Puerto Rico | 87 (81-92) | 2.75 (2.57-2.92) | 109 (89-129) | 1.34 (1.13-1.57) | -4.39 (-4.93--3.85) |
| Guam | 2 (2-3) | 5.4 (4.57-6.39) | 2 (2-3) | 1 (0.8-1.24) | -3.52 (-3.8--3.24) |
| Monaco | 0 (0-0) | 0.07 (0.06-0.09) | 0 (0-0) | 0.09 (0.07-0.12) | -5.69 (-5.93--5.45) |
| Northern Mariana Islands | 1 (0-1) | 5.77 (4.61-7.2) | 1 (1-1) | 4.51 (3.65-5.38) | -2.59 (-3.07--2.11) |
| Palau | 0 (0-0) | 3.55 (2.7-4.62) | 0 (0-0) | 2.37 (1.8-2.97) | -2.52 (-4.31--0.7) |
| South Sudan | 5690 (4021-8146) | 86.85 (66.06-114.55) | 3139 (2092-4657) | 33.9 (24.36-45.78) | -2.94 (-3.89--1.99) |
| Niue | 0 (0-0) | 5.98 (4.73-7.59) | 0 (0-0) | 5.18 (4.14-6.56) | -6 (-6.25--5.75) |

## Table S5：DALYs of Nutritional Deficiencies in cases and age-standardized rates for both sexes combined in 1990 and 2021, with percentage change between 1990 and 2021 by GBD 204 nations.

| DALYs | | | | | |
| --- | --- | --- | --- | --- | --- |
| nations | **1990** | | **2021** | | **1990-2021 EAPC** |
|  | **All-ages cases(both sexs)** | **Age-standardized rates per 100,000 people** | **All-ages cases(both sexs)** | **Age-standardized rates per 100,000 people** |  |
|  | **n (95% UI)** | **n (95% UI)** | **n (95% UI)** | **n (95% UI)** | **n (95% UI)** |
| Slovenia | 4527 (2986-6608) | 242.49 (158.42-354.54) | 2840 (1883-4341) | 139.67 (92.27-212.14) | -1.32 (-1.38--1.26) |
| North Macedonia | 8078 (5383-11470) | 413.73 (275.95-586.63) | 4949 (3200-7217) | 241.12 (156.36-354.84) | -0.48 (-0.75--0.21) |
| Australia | 11232 (7358-16966) | 66.46 (43.01-101.53) | 14543 (9611-23043) | 51.48 (32.34-86.49) | 0.47 (0.29-0.66) |
| Lithuania | 11201 (7583-16303) | 312.89 (214.35-453.07) | 5435 (3531-7917) | 205.41 (133.09-304.53) | -3.65 (-3.8--3.5) |
| New Zealand | 2811 (1749-4732) | 83.82 (51.11-149.01) | 3220 (1954-5323) | 62.81 (35.48-111.76) | -0.31 (-0.42--0.19) |
| Japan | 96873 (60284-151529) | 76.4 (46.82-121.1) | 121780 (84583-175771) | 63.92 (41.21-98.2) | -0.78 (-0.98--0.59) |
| Andorra | 33 (19-53) | 70.25 (41.28-111.09) | 64 (27-120) | 73.34 (32.73-131.81) | -1.48 (-1.58--1.39) |
| Taiwan (Province of China) | 44367 (31077-63831) | 246.12 (178.42-341.52) | 30434 (21002-44114) | 121.53 (83.54-179.62) | -2.99 (-3.09--2.9) |
| Democratic People's Republic of Korea | 129428 (92971-178166) | 617.99 (448.4-857.82) | 68478 (46306-97549) | 275.55 (186.72-390.29) | -4.76 (-5.2--4.32) |
| China | 6198310 (4969390-7962003) | 575.41 (467.01-729.98) | 2299911 (1564018-3318673) | 159.31 (109.39-226.47) | 0.21 (0.01-0.4) |
| Argentina | 122538 (101382-155223) | 371.27 (307.81-465.09) | 51942 (35279-79507) | 117.73 (77.41-183.77) | -1.33 (-1.48--1.19) |
| Malta | 379 (236-593) | 107.16 (67.2-168.62) | 296 (182-507) | 68.06 (41.44-111.73) | -6.21 (-6.75--5.67) |
| Israel | 5726 (3555-9080) | 115.27 (72.2-182.62) | 9449 (4804-17647) | 94.71 (47.39-176.08) | -0.68 (-0.73--0.63) |
| Fiji | 4929 (3413-6719) | 668.94 (473.24-908.82) | 4594 (3101-6710) | 524.68 (358.51-755.59) | -0.71 (-0.93--0.5) |
| Norway | 3130 (1937-4817) | 78.81 (48.24-119.83) | 6060 (4053-9472) | 86.38 (52.37-139.35) | -1.05 (-1.14--0.95) |
| Canada | 13532 (7944-23775) | 47.2 (27.57-82.8) | 55104 (17873-100255) | 114.93 (36.45-210.42) | -2.05 (-2.15--1.95) |
| Belarus | 33199 (22625-47466) | 327.76 (226-463.44) | 19531 (13319-28295) | 210.61 (143.16-309.95) | -1.98 (-2.2--1.77) |
| Papua New Guinea | 33197 (23327-45019) | 771.91 (552.65-1056.31) | 67619 (46250-102906) | 618.3 (433.88-923.71) | -3.27 (-3.66--2.88) |
| United Kingdom | 55819 (36686-83537) | 108.98 (70.64-162.59) | 54451 (34160-83689) | 90.14 (57.1-141.79) | -1.65 (-1.81--1.48) |
| Armenia | 21397 (15821-29123) | 608.1 (447.59-827.09) | 9200 (6013-13252) | 339.98 (224.22-494.33) | -2.48 (-2.63--2.33) |
| Lao People's Democratic Republic | 125049 (91334-192990) | 2212.57 (1684.05-3261.94) | 40975 (29901-56115) | 581.86 (433.17-785.93) | -2.89 (-3.08--2.7) |
| Maldives | 5970 (4344-8188) | 2072.54 (1543.92-2780.92) | 2352 (1543-3390) | 490.28 (335.54-708.29) | -1.25 (-1.39--1.12) |
| Timor-Leste | 35596 (22262-62783) | 3021.26 (1958.99-5155.62) | 10526 (7894-14349) | 736.34 (556.36-986.1) | -1.42 (-1.69--1.14) |
| Viet Nam | 587711 (425929-789944) | 819.18 (596.16-1092.87) | 349134 (240028-490848) | 360.27 (252.32-504.31) | -1.12 (-1.27--0.97) |
| Micronesia (Federated States of) | 1003 (732-1332) | 915.3 (682.76-1201.5) | 547 (379-780) | 586.42 (412.75-821.2) | -2.53 (-2.69--2.38) |
| Turkmenistan | 34602 (23782-47497) | 817.02 (567.38-1119.19) | 26580 (17751-39228) | 503.65 (335.2-741.91) | -2.1 (-2.19--2) |
| Chile | 28316 (23141-35141) | 220.96 (180.91-274.07) | 22937 (14079-37426) | 114.64 (72.66-181.56) | -2.75 (-3.07--2.44) |
| Switzerland | 4997 (3378-7613) | 73.61 (46.57-111.82) | 7561 (3640-17226) | 70.22 (31.59-153.7) | 0.75 (0.28-1.22) |
| Cambodia | 371385 (257572-534977) | 2733.1 (1988.45-3761.7) | 110361 (77085-154568) | 686.74 (488.4-940.46) | -2.03 (-2.23--1.82) |
| Indonesia | 2162579 (1745340-2706760) | 1265.32 (1049.02-1541.74) | 1337764 (1039538-1717162) | 594.45 (478.27-735.54) | -2.81 (-3.21--2.41) |
| Malaysia | 121400 (84445-171539) | 673.72 (474.22-950.54) | 119661 (77338-174171) | 393.83 (259.92-563.94) | -2.01 (-2.05--1.96) |
| Myanmar | 622406 (467048-818173) | 1512.38 (1163.37-1974.39) | 352265 (237279-503189) | 647.77 (441.73-919.19) | -10.58 (-15.44--5.43) |
| Samoa | 1239 (900-1709) | 693.11 (511.49-945.28) | 1063 (729-1518) | 491.68 (342.57-692.56) | -1.29 (-1.48--1.1) |
| Philippines | 674591 (541534-851043) | 976.9 (791.72-1240.82) | 624938 (445396-844296) | 589.77 (427.89-785.24) | -3.21 (-3.32--3.09) |
| Thailand | 289482 (210105-404900) | 565.84 (422.84-769.15) | 203284 (142789-281324) | 305.3 (214.99-422.46) | -4.53 (-4.82--4.24) |
| Kiribati | 2400 (1856-3048) | 2515.99 (2014.44-3127.16) | 1512 (1162-1983) | 1318.83 (1038.34-1700.74) | -1.71 (-2--1.43) |
| Georgia | 25023 (16329-36286) | 469.15 (309.47-680.68) | 12750 (8402-18804) | 370.87 (240.37-547.95) | -1.84 (-1.94--1.73) |
| Albania | 19801 (13355-27946) | 567.69 (382.02-803.76) | 5917 (4029-8668) | 237.81 (161.76-348.57) | -1.59 (-1.77--1.41) |
| Slovakia | 15618 (10077-23000) | 305.9 (194.58-449.12) | 9524 (5953-14276) | 182.02 (114.59-269.21) | -2.26 (-2.42--2.1) |
| Solomon Islands | 3552 (2671-4749) | 920.04 (699.8-1208.61) | 4698 (3299-6646) | 676.73 (486.67-941.57) | -4.5 (-4.72--4.28) |
| Kyrgyzstan | 33676 (23213-48254) | 703.44 (485.15-1000.27) | 33895 (21754-50440) | 475.76 (307.06-701.29) | -1.03 (-1.2--0.87) |
| Kazakhstan | 133841 (90183-189396) | 793.26 (535.8-1129.98) | 86074 (57571-130858) | 446.18 (296.77-680.08) | -0.57 (-0.75--0.38) |
| Vanuatu | 1318 (989-1735) | 778.91 (590.92-1029.72) | 2169 (1493-2984) | 700.14 (489.14-951.15) | -1.49 (-1.8--1.18) |
| Tajikistan | 56848 (40380-77054) | 892.98 (630.41-1220.28) | 72826 (49428-103600) | 674.13 (453.72-950) | -1.35 (-1.49--1.21) |
| Tonga | 718 (528-980) | 664.96 (489.26-907.5) | 573 (411-796) | 520.15 (375.22-715.2) | -0.85 (-0.92--0.77) |
| Antigua and Barbuda | 394 (293-520) | 658.53 (494.87-865.81) | 330 (235-474) | 407.46 (291.34-579.7) | -2.42 (-2.94--1.9) |
| Marshall Islands | 395 (278-537) | 831.44 (606.28-1112.39) | 354 (241-496) | 677.64 (473.4-931.72) | -3.19 (-3.34--3.05) |
| Mongolia | 22836 (15460-31961) | 950.84 (649.54-1330.68) | 20239 (13126-29666) | 575 (374.58-844.07) | -0.44 (-0.56--0.32) |
| Sri Lanka | 132327 (91880-184632) | 814 (575.64-1120.78) | 95377 (65130-135831) | 437.72 (299.11-621.38) | 0.65 (0.45-0.85) |
| Bosnia and Herzegovina | 17232 (10801-24962) | 396.21 (249.6-575.58) | 7100 (4537-10749) | 228 (143.04-342.63) | -1.9 (-2.08--1.72) |
| Bulgaria | 27883 (18057-40879) | 345.74 (222.49-510.81) | 15687 (10053-22153) | 244.65 (154.36-356.88) | -1.69 (-1.93--1.44) |
| Estonia | 5040 (3535-7124) | 330.88 (234.2-466.29) | 2291 (1530-3327) | 178.91 (117.89-257.24) | -1.27 (-1.34--1.21) |
| Serbia | 32512 (21133-47568) | 355.47 (231.2-519.04) | 17475 (11333-25757) | 203.97 (130.37-301.27) | -4.19 (-4.58--3.8) |
| Azerbaijan | 66921 (48031-91933) | 853.27 (605.23-1173.32) | 47420 (30426-67548) | 471.72 (305.12-677.06) | 3.38 (2.72-4.04) |
| Guyana | 16035 (13366-19385) | 1808.32 (1518.84-2177.38) | 5445 (4145-7261) | 738.68 (568.25-977.27) | -1.68 (-1.75--1.61) |
| Haiti | 251390 (193700-322512) | 2808.16 (2177.84-3575.27) | 186626 (135350-251086) | 1325.29 (961.19-1783.84) | -4.93 (-5.26--4.6) |
| Grenada | 743 (564-995) | 791.15 (599.28-1058.22) | 484 (344-656) | 506.68 (360.56-681.99) | -2.05 (-2.17--1.93) |
| Jamaica | 20064 (16127-25992) | 800.98 (641.61-1035.23) | 10896 (7540-15394) | 409.49 (282.48-578.41) | -1.72 (-1.97--1.47) |
| Uzbekistan | 242408 (161165-340399) | 1026.58 (698.66-1441.49) | 262592 (174594-378452) | 743.39 (494.74-1070.96) | -2.07 (-2.42--1.71) |
| Poland | 134637 (86662-199156) | 364.54 (234.59-544.99) | 72435 (47618-106985) | 195.29 (128.04-289.99) | -0.65 (-0.89--0.41) |
| Saint Vincent and the Grenadines | 1232 (975-1529) | 1113.43 (897.27-1368.67) | 699 (540-901) | 664.32 (513.98-853.27) | -5.71 (-5.94--5.48) |
| Czechia | 27428 (17626-40501) | 280.56 (180.29-421.09) | 19431 (13652-28056) | 175.55 (117.76-263.01) | -1.17 (-1.33--1) |
| Brunei Darussalam | 296 (188-468) | 130.07 (87.67-195.76) | 400 (250-619) | 92.67 (58.53-141.05) | -1.6 (-1.78--1.41) |
| Hungary | 31317 (20291-46116) | 320.66 (207.62-469.78) | 18086 (11858-25517) | 190.25 (123.46-277.12) | 2.11 (1.88-2.35) |
| Croatia | 11955 (7708-17840) | 260.39 (167.71-394.8) | 6584 (4311-9774) | 164 (104.62-247.6) | -2.72 (-2.81--2.63) |
| Ukraine | 207590 (147590-284243) | 419.93 (299.78-572.56) | 123899 (83861-177033) | 310.2 (207.35-446.23) | -1.3 (-1.41--1.19) |
| Latvia | 8649 (5970-12297) | 335.54 (231.95-471.95) | 3901 (2658-5562) | 212.59 (142.88-308.31) | -3.15 (-3.38--2.93) |
| Montenegro | 1571 (1021-2289) | 257.4 (168.29-373.59) | 1164 (758-1748) | 194.88 (125.57-286.52) | -0.46 (-0.56--0.36) |
| Romania | 89267 (58674-131058) | 403.51 (262.53-588.18) | 39950 (26671-59350) | 220.26 (144.61-332.77) | -2.09 (-2.4--1.77) |
| Singapore | 3665 (1875-6616) | 115.51 (62.71-198.27) | 2699 (1365-5167) | 44.22 (21.99-85.45) | -2.76 (-2.96--2.56) |
| Republic of Moldova | 16563 (11047-23511) | 372.98 (249.66-532.31) | 8694 (5651-12626) | 236.69 (158.25-344.43) | -2.4 (-2.63--2.17) |
| Russian Federation | 492095 (321506-727705) | 333.39 (218.63-499.42) | 317652 (217511-467573) | 220.21 (149.53-323.13) | -3.34 (-3.42--3.26) |
| Iceland | 178 (115-279) | 72.6 (46.55-114.64) | 224 (131-529) | 60.06 (34.05-131.49) | -2.63 (-2.69--2.58) |
| Finland | 4272 (2732-6687) | 90.68 (56.83-143.16) | 3466 (2228-5546) | 61.34 (37.43-94.32) | -1.59 (-1.76--1.43) |
| France | 85934 (66183-115349) | 140.81 (104.05-195.64) | 114864 (78116-237949) | 115.12 (70.51-252.24) | 0.17 (-0.03-0.38) |
| Belgium | 9428 (6308-13706) | 99.43 (64.38-150.17) | 13881 (7226-31187) | 99.51 (49.57-213.29) | -1.75 (-1.82--1.68) |
| Netherlands | 15638 (8440-30875) | 108.07 (60.01-204.38) | 24639 (9575-44121) | 120.02 (49.26-207.75) | -1.12 (-1.32--0.91) |
| Austria | 5875 (3602-9434) | 84.29 (50.36-135.29) | 5918 (3163-12266) | 65.36 (34.09-133.57) | -5.14 (-5.56--4.73) |
| Italy | 64606 (36461-106784) | 116.21 (67.23-188.39) | 71028 (40350-109317) | 101.39 (57.23-159.59) | -5.81 (-5.99--5.63) |
| Denmark | 5154 (3588-7448) | 100.16 (64.07-149.96) | 4609 (3174-7976) | 69.27 (43.24-118.7) | -3.19 (-3.3--3.07) |
| Cyprus | 717 (441-1120) | 100.6 (63.06-153.08) | 776 (488-1229) | 59.66 (37.31-93.83) | -2.03 (-2.18--1.88) |
| Peru | 318541 (251370-394003) | 1271.39 (1024.71-1579.61) | 125005 (93811-166594) | 358.24 (269.23-477.23) | -2.45 (-2.66--2.24) |
| Republic of Korea | 45370 (30570-65818) | 126.78 (90.6-177.91) | 29751 (20753-43563) | 51.91 (35.33-78.11) | -0.8 (-0.85--0.75) |
| Germany | 60667 (36245-98001) | 83.32 (49.46-132.96) | 108901 (36241-173176) | 103.37 (37.99-165.28) | -1.34 (-1.52--1.16) |
| Greece | 8216 (4998-12688) | 86.47 (52.6-132.75) | 7441 (4335-14553) | 73.48 (41.96-141.62) | -1.78 (-2.08--1.48) |
| Algeria | 189257 (131744-262718) | 671.87 (466.14-916.48) | 161841 (103295-239354) | 363.97 (234.18-537.08) | -2.42 (-2.59--2.25) |
| Ireland | 3269 (2028-4911) | 94.41 (58.11-141.39) | 3655 (1807-6906) | 71.42 (35.95-131.67) | -1.97 (-2.04--1.91) |
| Luxembourg | 339 (227-490) | 94.32 (61.71-136.08) | 447 (312-673) | 61.77 (40.74-96.56) | -1.7 (-1.84--1.56) |
| Uruguay | 7361 (5908-9876) | 245.9 (197.25-330.03) | 3732 (2785-5140) | 100.05 (70.06-148.69) | -0.05 (-0.16-0.05) |
| Spain | 32654 (19840-50172) | 92.93 (56.22-143.91) | 30300 (19792-45715) | 62.24 (37.08-97.06) | -2.62 (-2.91--2.33) |
| Portugal | 12610 (7852-18894) | 134.15 (84.35-203.3) | 9988 (6755-14611) | 82.26 (50.75-126.51) | -1.79 (-1.83--1.75) |
| Suriname | 3878 (2961-5175) | 964.35 (735.57-1278.71) | 2877 (1964-3968) | 524.78 (361.36-728.94) | -1.29 (-1.39--1.19) |
| Venezuela (Bolivarian Republic of) | 122854 (104597-150010) | 589.14 (501.14-716.25) | 72532 (54309-96709) | 288.6 (217.93-383.15) | -0.96 (-1.14--0.78) |
| Belize | 2725 (2301-3319) | 1167.47 (967.12-1440.3) | 2509 (1818-3405) | 606.41 (447.06-810.27) | -2.54 (-2.65--2.43) |
| Sweden | 6951 (4466-10424) | 82.37 (51.75-127.03) | 8829 (5049-17245) | 74.66 (40.21-142.96) | -1.68 (-1.75--1.62) |
| Bahamas | 1487 (1071-2073) | 582.75 (426.67-799.38) | 1391 (953-1987) | 388.77 (268.39-549.8) | -1.35 (-1.44--1.27) |
| Barbados | 1362 (1006-1827) | 562.16 (414.8-759.59) | 947 (646-1353) | 353.57 (236.51-517.1) | -2.11 (-2.2--2.01) |
| Dominica | 499 (361-667) | 667.52 (487.25-890.51) | 302 (210-407) | 514.94 (363.18-693.07) | -2.75 (-2.85--2.64) |
| Tunisia | 44511 (30799-61824) | 508.82 (353.19-704.78) | 36808 (25160-52533) | 315.31 (216.32-452.03) | -2.14 (-2.19--2.1) |
| Saudi Arabia | 116926 (83719-157238) | 649.13 (466.37-872.69) | 94702 (64072-135172) | 270.58 (186.85-378) | -2.21 (-2.45--1.97) |
| United States of America | 210192 (135249-321282) | 77.5 (49.49-119.34) | 584407 (439249-773722) | 139.56 (100.99-189.24) | -3.32 (-3.55--3.08) |
| Bolivia (Plurinational State of) | 220207 (163904-276784) | 2617.63 (1976.89-3252.73) | 80075 (60321-106096) | 713.11 (546.57-925.31) | -1.76 (-2.16--1.36) |
| Dominican Republic | 135880 (114675-160938) | 1550.04 (1307.35-1854.7) | 60590 (44743-80534) | 563.17 (416.71-746.06) | 1.34 (1.02-1.67) |
| Colombia | 208801 (177285-247603) | 598.96 (513.22-708.11) | 112986 (88362-146772) | 260.4 (201.66-341.81) | -4.54 (-4.84--4.25) |
| Trkiye | 433703 (317958-587458) | 712.54 (521.14-952.6) | 253654 (170481-354923) | 314.23 (212.86-441.62) | -2.2 (-2.36--2.05) |
| Saint Lucia | 1246 (931-1609) | 889.75 (674.51-1124.73) | 775 (553-1077) | 478.43 (340.98-661.76) | -1.35 (-1.5--1.2) |
| Cuba | 38367 (25221-55015) | 361.65 (240.22-523.47) | 27708 (18234-40602) | 273.58 (179.76-395.06) | -2.87 (-3.09--2.66) |
| Afghanistan | 169244 (129924-225093) | 1247.78 (963.31-1634.06) | 208906 (154413-278692) | 579.49 (434.63-769.03) | -1.55 (-4.11-1.07) |
| Bangladesh | 6327390 (5255758-7380709) | 4239.09 (3558.73-5004.43) | 1150503 (813181-1577845) | 733.97 (524.58-997.01) | -2.2 (-2.33--2.07) |
| Brazil | 1733097 (1423838-2126883) | 1150.47 (957.34-1408.55) | 940663 (664827-1294591) | 448.4 (316.22-615.99) | -2.31 (-2.62--1.99) |
| El Salvador | 41824 (34626-50486) | 679.36 (570.77-816.71) | 15834 (12189-20657) | 248.42 (191.18-325.57) | -3.77 (-3.95--3.58) |
| Trinidad and Tobago | 8703 (6279-11826) | 730.27 (537.85-970.31) | 4900 (3305-7040) | 386.76 (260.81-551.28) | -1.48 (-1.71--1.26) |
| Ecuador | 86470 (77588-97093) | 823.09 (744.74-917.37) | 31985 (25622-40360) | 191.82 (155.15-239.74) | -2.16 (-2.22--2.09) |
| Costa Rica | 6586 (4626-9254) | 210.48 (151.33-295.65) | 8782 (5267-12265) | 188.56 (116.5-267.38) | -0.91 (-1.1--0.73) |
| Guatemala | 290738 (264379-318244) | 4115.33 (3861.06-4396.31) | 103061 (81770-135899) | 706.68 (570.7-914.66) | -2.26 (-2.39--2.13) |
| India | 25217912 (20127721-32576500) | 2666.35 (2101.49-3470.81) | 15961180 (11216069-22180419) | 1207.13 (852.26-1670.62) | -2.1 (-2.23--1.98) |
| Bahrain | 2335 (1586-3353) | 470.77 (326.31-652.16) | 2918 (1867-4440) | 217.32 (145.22-322.88) | -1.27 (-1.38--1.16) |
| Mexico | 996182 (895196-1117465) | 1169.34 (1074.13-1289.65) | 337971 (277567-412162) | 285.13 (235.85-346.99) | -1.79 (-1.95--1.64) |
| Jordan | 22728 (14898-33382) | 563.86 (372.03-820.7) | 46644 (30083-69937) | 384.79 (252.31-572.87) | -0.63 (-0.82--0.43) |
| Palestine | 10276 (7153-15111) | 431.93 (299.2-624.94) | 12127 (7640-18161) | 238.25 (149.8-352.41) | -2.22 (-2.3--2.14) |
| Qatar | 1406 (911-2078) | 321.75 (210.36-463.26) | 4435 (2709-6791) | 172.78 (113.52-251.88) | -5.05 (-5.31--4.79) |
| Syrian Arab Republic | 122363 (89950-166022) | 813.29 (596.75-1121.53) | 62253 (41338-87316) | 482.67 (331.02-663.22) | -0.87 (-1.26--0.48) |
| Nicaragua | 39498 (32045-48313) | 774.21 (636.96-940.8) | 18623 (13780-25201) | 295.16 (221.13-391.61) | -2.87 (-3.23--2.51) |
| Iran (Islamic Republic of) | 401112 (299016-539970) | 617.12 (456.69-829.42) | 250695 (167470-372443) | 300.83 (203.86-441.9) | -2.96 (-3.39--2.52) |
| Honduras | 38989 (30675-49988) | 703.17 (566.07-878.9) | 32695 (24140-43725) | 365.08 (276.04-471.78) | -5.08 (-5.26--4.9) |
| Congo | 42392 (32555-55546) | 1534.63 (1186.88-1987.36) | 50908 (36401-71451) | 968.8 (712.41-1339.2) | -2.32 (-2.47--2.17) |
| Paraguay | 27853 (20946-37229) | 591.93 (443.37-810.88) | 27770 (20059-38283) | 403.48 (292.87-549.09) | -1.53 (-1.67--1.4) |
| Egypt | 382705 (276915-527220) | 640.37 (468.99-868.54) | 316513 (211925-457526) | 297.71 (201.11-430.06) | -0.69 (-0.75--0.64) |
| Panama | 13277 (10978-16147) | 554.23 (465.8-667.74) | 10278 (7855-13637) | 253.78 (195.04-335.37) | -0.16 (-0.32--0.01) |
| Comoros | 18049 (12938-23448) | 2808.33 (2098.99-3596.02) | 6852 (5080-9171) | 937.23 (697.65-1244.28) | -4.29 (-4.65--3.92) |
| Kuwait | 5235 (3389-7693) | 289.81 (189.04-418.92) | 10099 (6289-15515) | 219.39 (139.4-329.95) | 0.44 (0.06-0.83) |
| Iraq | 150364 (110404-203547) | 669 (490.91-910.5) | 144743 (94052-211783) | 352.1 (230.69-510.74) | -2.41 (-2.64--2.17) |
| United Arab Emirates | 9977 (6869-14529) | 512.21 (355.97-734.9) | 23643 (15276-36651) | 302.36 (200.82-447.12) | -4.47 (-4.62--4.32) |
| Angola | 905206 (665345-1312697) | 5487.34 (4117.48-7703.04) | 356600 (266631-473035) | 953.39 (728.7-1264.17) | -3.63 (-3.83--3.43) |
| Lebanon | 16539 (11110-24068) | 527.57 (351.67-760.22) | 17168 (11269-25523) | 314.01 (203.94-464.96) | -4.37 (-4.58--4.17) |
| Morocco | 204222 (146661-284064) | 722.96 (518.27-1008.49) | 148346 (100337-221290) | 409.67 (278.45-604.91) | -2.11 (-2.34--1.87) |
| Nepal | 870382 (657339-1173638) | 3432.08 (2653.06-4457.94) | 260384 (184293-359693) | 880.08 (628.71-1196.33) | -2.35 (-2.53--2.18) |
| Libya | 19671 (13095-28569) | 417.06 (274.7-596.86) | 21980 (14346-32409) | 341.54 (230.5-504.33) | -4.98 (-5.25--4.71) |
| Oman | 17067 (12917-23832) | 716.41 (550.21-957.52) | 11774 (7874-17178) | 276.12 (192.93-395.6) | -3.43 (-3.58--3.29) |
| Yemen | 490672 (366547-653827) | 2522.94 (1923.43-3290.31) | 652607 (477785-871096) | 1749.37 (1285.34-2318.29) | -1.51 (-1.58--1.45) |
| Burundi | 440774 (266187-737786) | 5130.98 (3300.92-8327.87) | 176137 (119893-249123) | 1177.7 (853.03-1573.69) | -0.86 (-0.99--0.72) |
| Malawi | 635542 (470646-857021) | 4101.84 (3178.79-5261.36) | 248178 (180677-332377) | 1177.3 (883.72-1547.33) | -4.87 (-5.17--4.58) |
| Bhutan | 11523 (7483-16775) | 1615.51 (1078.04-2302.13) | 7531 (4733-10789) | 1085.14 (681.3-1556.72) | 1.67 (1.35-2) |
| Pakistan | 2669591 (2055113-3495992) | 2050.29 (1574.16-2692.4) | 3398662 (2414518-4784070) | 1391.52 (989.37-1934.27) | -1.7 (-1.8--1.61) |
| Democratic Republic of the Congo | 1470617 (1037541-2031054) | 2610.77 (1927.06-3427.63) | 922746 (635680-1283724) | 975.53 (687.82-1363.78) | -4.5 (-4.63--4.37) |
| Central African Republic | 89767 (62442-130001) | 2253.73 (1634.73-3102.65) | 119291 (89165-159231) | 1866.94 (1449.29-2453.88) | -2.49 (-2.55--2.43) |
| Equatorial Guinea | 12806 (9694-16717) | 2289.49 (1753.03-2969.69) | 9545 (6469-14292) | 641.68 (441.43-925.97) | -1.46 (-1.65--1.27) |
| Gabon | 16007 (11794-21075) | 1482.21 (1114.82-1970.64) | 16442 (11163-23156) | 914.74 (638-1278.53) | -1.53 (-1.6--1.45) |
| Rwanda | 343787 (251734-466229) | 3206.54 (2478.53-4157.14) | 98041 (75651-126659) | 729.19 (572.18-933.89) | -3.26 (-3.36--3.15) |
| Uganda | 684774 (486007-930228) | 2282.69 (1728.36-2976.65) | 409929 (284385-556193) | 795.72 (580.17-1063.23) | -2.8 (-2.92--2.68) |
| Djibouti | 13900 (10481-17989) | 2777.85 (2127.93-3591.24) | 14343 (10268-19755) | 1180.91 (865.97-1596.02) | -1.53 (-1.63--1.44) |
| Eritrea | 333056 (236785-445560) | 7195.97 (5329.5-9364.95) | 102847 (78727-137034) | 1482.49 (1159.65-1925.82) | -0.51 (-0.63--0.4) |
| Kenya | 586888 (464908-738215) | 1693.44 (1413.61-2079.6) | 378195 (308037-467708) | 784.81 (643.94-960.08) | -1.43 (-1.49--1.38) |
| Madagascar | 1014314 (836977-1188090) | 5723.51 (4849.68-6595.15) | 513075 (383907-672775) | 1693.2 (1308.61-2159.81) | 0.44 (0.24-0.65) |
| Ethiopia | 3522016 (2703856-4652365) | 4928.48 (3880.91-6360.17) | 1379491 (1071116-1749762) | 1133.93 (895.04-1447.41) | -1.74 (-1.78--1.7) |
| Zambia | 418403 (311424-582080) | 3371.43 (2586.86-4447.39) | 253513 (185567-345401) | 1159.34 (855.46-1577.39) | -2.69 (-2.86--2.52) |
| Mauritius | 7169 (5205-9833) | 705.45 (520.21-948.22) | 4325 (2894-6247) | 360.45 (240.78-515.51) | -2.69 (-2.79--2.59) |
| Namibia | 28388 (21758-36809) | 1551.78 (1220.3-1986.92) | 18250 (13108-25059) | 720.65 (529.21-982.99) | -0.79 (-0.89--0.68) |
| Cameroon | 162078 (121399-211568) | 1116.96 (848.12-1451.73) | 231264 (163961-325776) | 656.53 (476.62-904.35) | -2.36 (-2.5--2.21) |
| Guinea-Bissau | 42988 (30783-59784) | 2966.23 (2216.18-4024.83) | 24981 (16759-35079) | 1116.06 (771.58-1554.2) | -2.05 (-2.22--1.88) |
| Senegal | 260654 (199260-333468) | 2403.16 (1822.93-3083.4) | 193600 (127096-275417) | 1100.48 (732.04-1564.24) | -2.01 (-2.14--1.88) |
| Togo | 85023 (62163-112681) | 1647.55 (1230.03-2153.93) | 98763 (66640-145323) | 1060.03 (729.99-1528.34) | -0.21 (-0.35--0.08) |
| Lesotho | 28556 (22438-36199) | 1505.14 (1172.6-1897.62) | 19783 (14950-26087) | 1045.24 (802.16-1358.77) | -3.35 (-3.51--3.2) |
| Mozambique | 881551 (648370-1235579) | 4441 (3329.65-5989.88) | 580853 (442061-758930) | 1765.3 (1370.51-2270.05) | -0.49 (-0.64--0.33) |
| Botswana | 19006 (14652-24579) | 1139.59 (890.64-1460.82) | 17713 (12664-23678) | 759.46 (549.27-1013.2) | -3.77 (-4.11--3.42) |
| Zimbabwe | 186954 (143294-236345) | 1454.83 (1158.54-1819.64) | 306634 (238546-387453) | 1750.18 (1373.15-2156.75) | -4.57 (-4.9--4.24) |
| Seychelles | 381 (274-524) | 534.64 (387.52-724.98) | 343 (240-480) | 341.26 (239.94-471.38) | -0.67 (-0.85--0.48) |
| Eswatini | 13393 (10604-16978) | 1195.7 (955.3-1495.16) | 9386 (6926-12344) | 761.52 (565.38-985.94) | -1.42 (-1.53--1.3) |
| Cook Islands | 71 (45-104) | 370.81 (240.44-536.05) | 41 (27-64) | 245.94 (160.36-377.68) | -1.35 (-1.59--1.12) |
| United Republic of Tanzania | 1435117 (1121651-1816480) | 3680.04 (2966.92-4558.81) | 689655 (517434-945040) | 1032.82 (787.91-1412.66) | -2.1 (-2.2--1.99) |
| Bermuda | 169 (112-245) | 302.23 (205.15-435) | 91 (60-132) | 163.43 (105.96-240.05) | -0.95 (-1.04--0.86) |
| Benin | 191435 (131311-276083) | 2520.92 (1805.16-3408.26) | 141134 (99423-198401) | 922.5 (659.14-1276.77) | -0.47 (-0.6--0.34) |
| Somalia | 583203 (416167-789031) | 5154.5 (3866.81-6633.82) | 563498 (408587-750440) | 2203.79 (1650.34-2858.24) | -2.41 (-2.54--2.27) |
| Saint Kitts and Nevis | 549 (461-665) | 1318.38 (1109.98-1591.08) | 306 (236-391) | 613.85 (476.92-774) | -5.34 (-5.61--5.08) |
| South Africa | 715903 (579995-866672) | 1640.77 (1327.16-1999.49) | 494757 (373647-642720) | 930.34 (708.89-1193.91) | -0.73 (-0.83--0.62) |
| Mali | 688886 (488575-953811) | 4743.32 (3462.6-6384.02) | 947227 (714706-1225293) | 2742.3 (2138.04-3495.28) | -1.93 (-2.05--1.82) |
| Sudan | 571145 (396877-838598) | 1978 (1414.91-2771.47) | 316965 (221074-459438) | 686 (481.43-985.56) | -3.67 (-4.02--3.31) |
| Cabo Verde | 7909 (6054-10259) | 1644.5 (1282.95-2115.76) | 2902 (1928-4193) | 546.9 (371.24-782.46) | -1.66 (-1.79--1.53) |
| Chad | 320831 (230137-443806) | 3259.02 (2457.93-4332.95) | 349660 (257199-465575) | 1454.65 (1091.61-1944.62) | -1.19 (-1.27--1.12) |
| Burkina Faso | 469457 (346360-631081) | 2980.28 (2253.26-3889.42) | 479454 (352165-636375) | 1521.86 (1137.64-2021.71) | -3.64 (-3.85--3.43) |
| Gambia | 35863 (28005-45449) | 2559.94 (2015.3-3247.24) | 34692 (24943-46972) | 1312.28 (955.34-1788.55) | -1.85 (-1.98--1.71) |
| Cte d'Ivoire | 242398 (182192-319094) | 1408.07 (1083.01-1809.31) | 264720 (181559-364255) | 844.01 (585.39-1166.64) | -3.84 (-4.28--3.39) |
| Guinea | 297774 (215984-400342) | 2975.34 (2212.85-3945.7) | 216378 (158468-288729) | 1262.97 (935.64-1651.12) | -0.92 (-1.13--0.71) |
| Sao Tome and Principe | 4642 (3545-5748) | 2577.37 (1989.49-3203.25) | 1700 (1192-2470) | 772.02 (552.85-1099.17) | -4.26 (-4.41--4.11) |
| American Samoa | 325 (242-431) | 643.8 (492.55-845.8) | 225 (163-305) | 516.64 (378.96-684.13) | -2.9 (-3.37--2.43) |
| Liberia | 159090 (118626-220143) | 4011.45 (3073.54-5310.33) | 75261 (54643-99234) | 1316.63 (973-1718.55) | -0.75 (-0.85--0.65) |
| San Marino | 16 (10-27) | 71.99 (43.53-120.04) | 23 (12-52) | 66.41 (34.9-139.09) | -1.49 (-1.64--1.33) |
| Tokelau | 12 (9-17) | 764.96 (575.37-1023.01) | 7 (6-10) | 622.1 (456.42-813.41) | -0.4 (-0.52--0.29) |
| Tuvalu | 132 (104-173) | 1162.47 (922.96-1496.92) | 65 (45-91) | 546.88 (389.06-763.11) | -2.96 (-3.27--2.65) |
| United States Virgin Islands | 499 (364-700) | 474.94 (349.83-661.54) | 218 (153-311) | 292.77 (200.86-422.02) | -1.75 (-1.98--1.51) |
| Ghana | 475132 (349142-641297) | 2337.38 (1774.9-3031.42) | 362745 (261343-497595) | 1028.49 (757.23-1395.68) | -0.89 (-1.12--0.67) |
| Mauritania | 62679 (45174-85895) | 2335.17 (1721.1-3129.75) | 39547 (26236-57191) | 826.52 (551.7-1169.97) | -0.89 (-1.15--0.63) |
| Niger | 604692 (414509-914479) | 4063.96 (2914.06-5880.06) | 384774 (276695-524964) | 1088.38 (784.39-1494.8) | -1.68 (-1.79--1.56) |
| Sierra Leone | 266580 (193460-354672) | 3797.52 (2818.8-4980.46) | 334368 (232649-474303) | 2901.99 (2106.2-3992.21) | -2.96 (-3.25--2.67) |
| Greenland | 54 (34-83) | 112.62 (77.93-165.45) | 48 (31-83) | 84 (54.44-138.94) | -1.26 (-1.42--1.1) |
| Nigeria | 1860323 (1439190-2474553) | 1477.77 (1131.74-1961.3) | 2437443 (1669787-3420437) | 887.21 (607.06-1245.89) | -2.17 (-2.46--1.89) |
| Nauru | 88 (64-119) | 780.6 (582.18-1052.7) | 67 (48-94) | 614.68 (442.01-847.17) | -1.34 (-1.42--1.26) |
| Puerto Rico | 13024 (8970-18382) | 370.14 (256.76-520.84) | 7065 (5016-9902) | 228.49 (155.24-329.23) | -0.96 (-1.12--0.79) |
| Guam | 482 (342-678) | 378.54 (274.23-515.71) | 453 (307-646) | 285.41 (193.32-410.3) | -2.15 (-2.3--2) |
| Monaco | 18 (9-42) | 57.8 (29.8-122.97) | 28 (10-55) | 62.21 (25.01-121.08) | -5.98 (-6.15--5.81) |
| Northern Mariana Islands | 153 (105-219) | 384.28 (284.26-528.87) | 143 (100-210) | 338.72 (244.38-482.43) | -0.94 (-1.02--0.86) |
| Palau | 75 (51-105) | 520.38 (363.2-714.34) | 59 (39-87) | 374.14 (249.98-538.23) | -1.62 (-1.82--1.42) |
| South Sudan | 507442 (365123-721784) | 5776.59 (4284.46-7907.22) | 315676 (226312-449862) | 2426.56 (1814.2-3306.57) | 0.06 (-0.43-0.55) |
| Niue | 14 (10-20) | 610.25 (444.24-824.66) | 8 (6-11) | 573.98 (429.25-771.85) | -1.77 (-2.02--1.51) |

## Table S6: Incidence of Nutritional Deficiencies in cases and age-standardized rates by sex, from 1990 to 2021 by year.

| **Incidence** | | | | |
| --- | --- | --- | --- | --- |
| Year | Counts(M) | Counts(F) | Age-standardized Rate(M)(per 100,000) | Age-standardized Rate(F)(per 100,000) |
| 1990 | 574970672 (546730045,605735858) | 389911024 (371980319,408135288) | 20170.14 (19158.18-21173.1) | 14000.41 (13368.81-14655.15) |
| 1991 | 569357622 (542388981,599132233) | 389098363 (371331162,405587943) | 19712.07 (18765.39-20700.72) | 13808.65 (13197.4-14415.91) |
| 1992 | 563505993 (536779015,592431122) | 388057962 (370889873,404333214) | 19272.46 (18369.05-20224.46) | 13623.36 (13033.71-14201.89) |
| 1993 | 557687211 (531086193,586090660) | 386984943 (370647979,402810713) | 18860.76 (17999.94-19780.18) | 13450.5 (12888.51-14006.08) |
| 1994 | 551743437 (525379947,580884785) | 385799469 (369773207,401693952) | 18472.6 (17623.05-19406.87) | 13287.87 (12730.44-13834.61) |
| 1995 | 546080415 (520027719,575499279) | 384664142 (369121064,400747955) | 18111.51 (17283.9-19074.25) | 13135.62 (12596.81-13692.29) |
| 1996 | 540421527 (515228305,569841833) | 383350191 (367631445,399273033) | 17759.47 (16975.63-18705.54) | 12978.8 (12449.72-13523.32) |
| 1997 | 534297023 (509819750,563507147) | 381504084 (366293592,397675418) | 17400.63 (16604.05-18327.07) | 12805.34 (12295.38-13345.23) |
| 1998 | 527977164 (503626304,556323507) | 379277879 (364361716,394913702) | 17044.8 (16265.1-17953.33) | 12622.6 (12116.37-13153.91) |
| 1999 | 521863388 (497977504,550559782) | 376994909 (360952671,394122697) | 16703.12 (15954.49-17610.68) | 12440.42 (11929.06-13000.55) |
| 2000 | 516391410 (491770272,544742144) | 375035744 (358034354,393107242) | 16385.33 (15622.27-17246.24) | 12269.13 (11725.78-12848.04) |
| 2001 | 510748338 (486000736,538084125) | 372806834 (356286433,390449092) | 16063.1 (15306.1-16886.66) | 12087.11 (11556.94-12645.5) |
| 2002 | 504332086 (479752245,529993585) | 369839763 (354090322,387257385) | 15714.51 (14959.16-16489.93) | 11877.83 (11375.38-12438.21) |
| 2003 | 497520740 (472386970,523807752) | 366521159 (350338942,383741978) | 15353.83 (14596.03-16140.74) | 11655.66 (11149.2-12204.08) |
| 2004 | 490426113 (465023598,517998554) | 363079063 (346310997,380205368) | 14986.47 (14231.03-15789.04) | 11429.66 (10924.81-11981.83) |
| 2005 | 483416316 (457900475,512497882) | 359842549 (343354736,377418413) | 14624.55 (13872.45-15460.12) | 11210.98 (10717-11771.57) |
| 2006 | 475786803 (450154284,504247379) | 356247069 (340050974,374289414) | 14244.14 (13503.69-15071.05) | 10980.5 (10490.13-11523) |
| 2007 | 466770358 (441293967,493431635) | 351655113 (335633885,369592239) | 13821.86 (13087.53-14595.79) | 10718.92 (10227.81-11263.49) |
| 2008 | 456990681 (431542630,483425387) | 346460109 (329714808,364602242) | 13378.73 (12657.15-14137.96) | 10440.15 (9942.51-10973.69) |
| 2009 | 447171700 (422493780,473656951) | 341122080 (324693835,359479250) | 12941.62 (12238.14-13695.64) | 10161.12 (9682.78-10697.29) |
| 2010 | 437851766 (412672138,463639713) | 336024267 (319823867,354802930) | 12538.88 (11839.48-13282.9) | 9901.39 (9437.44-10444.15) |
| 2011 | 429128927 (405247383,455054966) | 331026793 (314591709,348925919) | 12171.14 (11478.36-12896.48) | 9656.22 (9199.31-10180.37) |
| 2012 | 420646993 (396825159,446507752) | 325790997 (309621908,343728483) | 11814.03 (11146.83-12504.46) | 9406.61 (8961.76-9918.37) |
| 2013 | 412179040 (388294981,436895220) | 320347769 (304469942,338297383) | 11459.43 (10804.24-12132.06) | 9153.34 (8721.87-9646.27) |
| 2014 | 403318548 (379566721,427923439) | 314601360 (298798546,332909329) | 11100.17 (10458.41-11765.84) | 8897.2 (8472.81-9398.66) |
| 2015 | 393786478 (369949290,418290407) | 308539483 (292912701,327003982) | 10727.16 (10090.92-11379.93) | 8636.71 (8207.81-9136.86) |
| 2016 | 380782566 (358136431,403678111) | 300082271 (285251589,317573844) | 10266.34 (9663.23-10867.39) | 8313.43 (7909.36-8789.39) |
| 2017 | 364055720 (343154568,385950897) | 288868165 (275290307,305025380) | 9720.57 (9163.54-10299.08) | 7923.6 (7555.28-8363.36) |
| 2018 | 347319609 (328147536,367962119) | 277573343 (265024598,292819554) | 9194.9 (8689.26-9740.69) | 7546.24 (7200.35-7961.33) |
| 2019 | 334404359 (316569711,354526576) | 268953095 (256696497,283377863) | 8789.3 (8319.05-9320.11) | 7257.16 (6921.14-7642.93) |
| 2020 | 328834655 (311459660,348162753) | 266139953 (254314154,279942623) | 8592.39 (8141.14-9095.33) | 7136.39 (6819.5-7506.99) |
| 2021 | 323658105 (306984148,343250270) | 262471282 (250683006,276642939) | 8423.99 (7987.09-8934.09) | 7006.41 (6700.12-7383.65) |
| Percent change between 1990 and 2021 | -0.44 (-0.47,-0.4) | -0.33 (-0.36,-0.29) | -0.58 (-0.61,-0.55) | -0.5 (-0.53,-0.47) |

## Table S7: Prevalence of Nutritional Deficiencies in cases and age-standardized rates by sex, from 1990 to 2021 by year.

| **Prevalence** | | | | |
| --- | --- | --- | --- | --- |
| Year | Counts(M) | Counts(F) | Age-standardized Rate(M)(per 100,000) | Age-standardized Rate(F)(per 100,000) |
| 1990 | 863913113 (842154181,887514594) | 901197651 (887274921,917398451) | 31249.65 (30494.23-32050.1) | 33315.43 (32809.58-33919.87) |
| 1991 | 862491327 (841226573,884829298) | 908367007 (895060241,924058050) | 30771.79 (30041.61-31518.8) | 33118.18 (32632.83-33715.44) |
| 1992 | 860541669 (840177857,882140671) | 915054961 (901985910,930802227) | 30308.51 (29620.41-31033.1) | 32925.54 (32461.65-33505.97) |
| 1993 | 858374186 (837980807,880207911) | 921571541 (908590828,937083660) | 29871.92 (29185.76-30589.89) | 32746.53 (32285.69-33317.76) |
| 1994 | 855805578 (835167771,877523846) | 927870954 (914547413,944150341) | 29458.75 (28805.92-30191.51) | 32583.5 (32111.7-33164.43) |
| 1995 | 853522390 (834642415,875196365) | 934375727 (921051097,950838096) | 29077.46 (28448.34-29810.99) | 32438.63 (31967.89-33009.39) |
| 1996 | 851303888 (831914145,872987541) | 940492702 (927125838,956790171) | 28707.68 (28102.33-29400.11) | 32281.48 (31821.04-32834.74) |
| 1997 | 848354917 (829422868,869418287) | 945574070 (932025744,961387183) | 28320.89 (27706.95-28989.45) | 32088.78 (31629.13-32617.13) |
| 1998 | 845253352 (826732178,866049046) | 950383473 (936809669,965900277) | 27939.1 (27333.25-28605.49) | 31888.72 (31431.41-32407.61) |
| 1999 | 842791966 (823679353,864531234) | 955607615 (941739568,971291647) | 27586.03 (26996.3-28257.1) | 31703.72 (31236.89-32227.29) |
| 2000 | 841262053 (821652202,863650137) | 962258651 (948179126,978517075) | 27266.39 (26663.09-27943.09) | 31561.07 (31097.5-32083.47) |
| 2001 | 840086231 (820555800,861252548) | 969815058 (955825308,986433705) | 26954.45 (26354.47-27601.84) | 31441.46 (30978.6-31973.05) |
| 2002 | 838639808 (819088464,859624080) | 977481504 (963110692,994504169) | 26625.59 (26038.01-27257.74) | 31313.87 (30862.92-31862.17) |
| 2003 | 836965377 (817190071,858312220) | 985210233 (970519866,1002315820) | 26286.73 (25708.86-26929.31) | 31179.7 (30712.41-31728.09) |
| 2004 | 834961344 (814965875,856881980) | 992874009 (977431350,1010268174) | 25936.35 (25325.8-26598.2) | 31037.25 (30557.23-31579.52) |
| 2005 | 832815531 (811965770,855476694) | 1000370295 (984658071,1017852869) | 25582.91 (24981.44-26250.98) | 30884.4 (30399.69-31428.98) |
| 2006 | 829498286 (809354494,852271089) | 1006625036 (990690947,1024861827) | 25190.43 (24597.63-25846.53) | 30687.91 (30212.76-31239.19) |
| 2007 | 824199443 (804504272,846714057) | 1011209835 (995692318,1030074655) | 24734.34 (24166.97-25376.61) | 30435.09 (29971.25-30987.14) |
| 2008 | 818068468 (798877211,840413630) | 1015279055 (1000074158,1034892422) | 24252.6 (23706.73-24891.49) | 30163.67 (29713.35-30730.04) |
| 2009 | 812374536 (792960725,834337065) | 1019948995 (1004228370,1040036432) | 23790.39 (23244.23-24421.33) | 29912.47 (29454.26-30489.67) |
| 2010 | 808056878 (787937319,830610002) | 1026087817 (1009421625,1046994476) | 23394.82 (22838.92-24031.52) | 29725.07 (29247.36-30326.92) |
| 2011 | 805074302 (785598685,827062221) | 1033589856 (1016204545,1054357319) | 23060.54 (22514.11-23670.33) | 29597.53 (29112.59-30188.73) |
| 2012 | 802505439 (783278080,824336326) | 1041521969 (1023494570,1062174666) | 22736.5 (22205.48-23333.89) | 29480.62 (28983.12-30061.48) |
| 2013 | 800008834 (780617372,821950697) | 1049521756 (1030932909,1070774927) | 22412.17 (21888.88-23007.85) | 29364.14 (28860.96-29940.49) |
| 2014 | 797049780 (778179910,818648660) | 1057105414 (1038172226,1078583917) | 22080.21 (21565.12-22663.19) | 29242.48 (28726.86-29819.23) |
| 2015 | 793374424 (773668900,815296640) | 1063938264 (1044018868,1085706297) | 21731.9 (21209.72-22316.83) | 29104.4 (28566.38-29675.26) |
| 2016 | 786891079 (768298386,807010151) | 1068869379 (1048237796,1090088911) | 21313.5 (20821.33-21843.98) | 28919.96 (28373.3-29470.29) |
| 2017 | 777248565 (759245150,795664792) | 1071908937 (1050700235,1092501923) | 20830.29 (20356.01-21314.06) | 28698.58 (28147.27-29223.59) |
| 2018 | 767177696 (749368427,784955217) | 1074676580 (1053183312,1095839276) | 20365.95 (19904.23-20834.93) | 28492.09 (27938.98-29031.24) |
| 2019 | 759723357 (742794258,777740780) | 1078922286 (1057789775,1100521724) | 19999.17 (19556.13-20463.15) | 28346.7 (27801.33-28899.84) |
| 2020 | 756427101 (738413618,775114553) | 1085500742 (1064791372,1106452509) | 19775.11 (19308.85-20258.1) | 28287.38 (27771.08-28812.9) |
| 2021 | 753569459 (735303455,772046911) | 1091677099 (1071437522,1113428733) | 19598.06 (19144.93-20066.79) | 28247.48 (27750.73-28804.58) |
| Percent change between 1990 and 2021 | -0.13 (-0.16,-0.1) | 0.21 (0.19,0.23) | -0.37 (-0.39,-0.35) | -0.15 (-0.17,-0.14) |

## Table S8: deaths of Nutritional Deficiencies in cases and age-standardized rates by sex, from 1990 to 2021 by year.

| **Deaths** | | | | |
| --- | --- | --- | --- | --- |
| Year | Counts(M) | Counts(F) | Age-standardized Rate(M)(per 100,000) | Age-standardized Rate(F)(per 100,000) |
| 1990 | 264756 (220967,320959) | 305363 (251850,370281) | 10.71 (9.28-12.56) | 11.4 (9.51-13.61) |
| 1991 | 262030 (219067,317025) | 299585 (249871,361251) | 10.53 (9.11-12.35) | 11.11 (9.35-13.19) |
| 1992 | 258230 (216911,313129) | 294509 (247614,356567) | 10.32 (8.92-12.12) | 10.87 (9.25-12.97) |
| 1993 | 248557 (209477,301653) | 279848 (236115,335164) | 9.86 (8.57-11.57) | 10.26 (8.71-12.15) |
| 1994 | 292150 (232486,378420) | 324001 (261465,419154) | 11.54 (9.38-14.97) | 11.95 (9.66-15.49) |
| 1995 | 267833 (216522,335874) | 296319 (243460,372611) | 10.46 (8.67-13.01) | 10.78 (8.91-13.48) |
| 1996 | 260217 (210815,327928) | 286975 (237715,364284) | 10.11 (8.46-12.56) | 10.37 (8.61-13.14) |
| 1997 | 251478 (204168,316663) | 277659 (228482,351597) | 9.71 (8.07-12.09) | 9.96 (8.24-12.59) |
| 1998 | 244837 (200370,309549) | 269107 (220083,341300) | 9.36 (7.83-11.69) | 9.57 (7.86-12.13) |
| 1999 | 183158 (159852,211290) | 202352 (177183,235948) | 6.9 (6.14-7.82) | 6.97 (6.12-8.1) |
| 2000 | 236670 (194430,301755) | 259518 (213478,331422) | 8.97 (7.49-11.28) | 9.14 (7.56-11.65) |
| 2001 | 174143 (153326,200368) | 191607 (169249,222326) | 6.49 (5.83-7.32) | 6.51 (5.76-7.54) |
| 2002 | 167366 (147666,190509) | 184195 (164377,212123) | 6.15 (5.52-6.9) | 6.17 (5.5-7.1) |
| 2003 | 161626 (142786,183818) | 178503 (159123,204217) | 5.85 (5.26-6.54) | 5.89 (5.25-6.73) |
| 2004 | 152930 (135123,172824) | 168295 (151311,187830) | 5.39 (4.86-6) | 5.36 (4.81-5.99) |
| 2005 | 157069 (139119,178213) | 172991 (156027,195062) | 5.61 (5.07-6.26) | 5.61 (5.05-6.32) |
| 2006 | 148031 (131240,167127) | 163383 (147594,181703) | 5.14 (4.63-5.71) | 5.11 (4.62-5.7) |
| 2007 | 158867 (142138,176626) | 171897 (156083,190068) | 5.39 (4.89-5.94) | 5.29 (4.8-5.86) |
| 2008 | 245153 (226619,263417) | 245278 (226787,263794) | 7.79 (7.23-8.34) | 7.44 (6.87-8.01) |
| 2009 | 127055 (112153,142712) | 142876 (129408,158692) | 4.06 (3.64-4.52) | 4.02 (3.62-4.49) |
| 2010 | 156223 (140152,171735) | 169333 (153377,184635) | 5.13 (4.65-5.59) | 5.05 (4.57-5.52) |
| 2011 | 132911 (118351,148558) | 148852 (135170,164511) | 4.37 (3.95-4.83) | 4.35 (3.94-4.83) |
| 2012 | 129660 (114763,145659) | 145472 (131686,161418) | 4.21 (3.78-4.69) | 4.18 (3.77-4.65) |
| 2013 | 125277 (111323,140920) | 140386 (126400,155796) | 3.95 (3.55-4.4) | 3.87 (3.46-4.33) |
| 2014 | 122143 (109191,138549) | 137244 (124017,152142) | 3.79 (3.42-4.25) | 3.71 (3.34-4.15) |
| 2015 | 118608 (105487,134420) | 133163 (120882,147836) | 3.64 (3.25-4.1) | 3.54 (3.19-3.97) |
| 2016 | 274038 (220929,345069) | 303976 (249689,381423) | 10.77 (8.93-13.36) | 11.13 (9.19-14.02) |
| 2017 | 105634 (93230,120200) | 116640 (103335,129658) | 3.16 (2.8-3.58) | 2.96 (2.63-3.33) |
| 2018 | 253841 (214731,307396) | 286992 (240383,348167) | 10.11 (8.74-11.82) | 10.56 (8.93-12.65) |
| 2019 | 114864 (102172,130546) | 129007 (115275,143449) | 3.49 (3.12-3.94) | 3.38 (3.01-3.79) |
| 2020 | 228165 (187398,292593) | 250341 (208061,321600) | 8.57 (7.17-10.86) | 8.73 (7.27-11.21) |
| 2021 | 110109 (97412,124573) | 121355 (107699,134615) | 3.31 (2.95-3.74) | 3.12 (2.78-3.53) |
| Percent change between 1990 and 2021 | -0.6 (-0.66,-0.52) | -0.62 (-0.69,-0.54) | -0.71 (-0.74,-0.65) | -0.74 (-0.78,-0.69) |

## Table S9: DALYs of Nutritional Deficiencies in cases and age-standardized rates by sex, from 1990 to 2021 by year.

| **DALYs** | | | | |
| --- | --- | --- | --- | --- |
| Year | Counts(M) | Counts(F) | Age-standardized Rate(M)(per 100,000) | Age-standardized Rate(F)(per 100,000) |
| 1990 | 34427040 (28554310,41851272) | 44247184 (36250406,55715793) | 1171.63 (967.32-1430.87) | 1573.03 (1282.74-1994.48) |
| 1991 | 34125333 (28296519,41455754) | 43874138 (35930009,55316750) | 1153.96 (954.5-1408.17) | 1547.88 (1267.68-1961.87) |
| 1992 | 33758656 (27954114,41222478) | 43528673 (35626277,54914670) | 1136.07 (942.2-1389.82) | 1526.08 (1246.26-1934.2) |
| 1993 | 33326459 (27619889,40675127) | 43023073 (35148896,53974589) | 1117.06 (923.78-1368.38) | 1499.66 (1225.29-1888.73) |
| 1994 | 32841024 (27262932,40285936) | 42532855 (34639396,53729802) | 1097.14 (911.77-1348.36) | 1474.76 (1201.79-1867.9) |
| 1995 | 35825836 (29546119,44826681) | 45266157 (36836703,57328629) | 1192.07 (985.76-1483.26) | 1561.86 (1269.55-1976.9) |
| 1996 | 34417071 (28605185,42406904) | 44013040 (35659508,55607613) | 1141.96 (951.25-1402.67) | 1510.13 (1225.25-1906.03) |
| 1997 | 33840034 (28213616,41729053) | 43507170 (35366509,54994251) | 1119.07 (935.3-1378.26) | 1483.85 (1207.11-1870.94) |
| 1998 | 33139040 (27554346,40982117) | 42859300 (34680163,54593466) | 1092.18 (909.34-1349.18) | 1452.51 (1176.84-1846.26) |
| 1999 | 32411643 (26925560,40092553) | 42261361 (34169383,54074702) | 1063.8 (885.01-1314.31) | 1422.29 (1151.8-1814.65) |
| 2000 | 31855949 (26429376,39506284) | 41781378 (33712471,53781268) | 1040.15 (864.33-1287.81) | 1395.16 (1128.63-1788.78) |
| 2001 | 31154065 (25746208,38886658) | 41176278 (33122116,53450854) | 1011.16 (837.3-1259.13) | 1363.04 (1099.91-1761.01) |
| 2002 | 30416281 (25130392,38170622) | 40571230 (32751700,52851036) | 980.37 (811.94-1227.87) | 1330.08 (1078.59-1726.23) |
| 2003 | 27166451 (22220363,34048030) | 37665919 (30236248,49200321) | 869.37 (713.19-1085.69) | 1219.57 (981.74-1585.97) |
| 2004 | 26425706 (21578778,33117840) | 37044305 (29521365,48563059) | 838.21 (686.48-1046.91) | 1185.91 (948.97-1548.27) |
| 2005 | 25857402 (21072943,32479808) | 36647338 (29190013,47877716) | 812.29 (663.91-1016.57) | 1159.5 (927.44-1510.18) |
| 2006 | 25326767 (20450087,31888743) | 36334481 (28698365,47629750) | 787.01 (637.05-987.31) | 1135.38 (900.35-1483.45) |
| 2007 | 24752577 (19770195,31178437) | 35947181 (28268056,47016186) | 760.45 (609.07-954.53) | 1108.74 (874.33-1445.13) |
| 2008 | 24181867 (19324507,30366190) | 35560371 (27815529,46344743) | 734.26 (588.51-919.87) | 1082.4 (849.05-1405.43) |
| 2009 | 23583261 (18795671,29701657) | 35174035 (27539181,45929005) | 707.99 (565.11-890.22) | 1056.87 (829.63-1374.89) |
| 2010 | 24053621 (19297998,30104941) | 35708890 (27951942,46573314) | 714.99 (575.22-893.47) | 1061.8 (835.03-1378.1) |
| 2011 | 29928712 (25306991,36112599) | 40876366 (32894140,51767135) | 879.76 (744.79-1060.48) | 1210.35 (980.9-1525.85) |
| 2012 | 23583354 (18876365,29755390) | 35588255 (27311194,46511464) | 688.34 (552.55-867.65) | 1036.19 (802.44-1349.1) |
| 2013 | 21863454 (17271403,27895535) | 34223874 (25966166,45137838) | 631.45 (501.08-804.17) | 982.9 (751.94-1293.05) |
| 2014 | 21444196 (16898444,27372555) | 33967203 (25521591,44874289) | 613 (484.58-781.11) | 964.45 (731.41-1270.1) |
| 2015 | 21060789 (16447103,26911882) | 33744811 (25259318,44563274) | 595.68 (467.09-759.06) | 947.27 (715.61-1247.13) |
| 2016 | 20608684 (16026753,26283962) | 33468308 (24858120,44379331) | 576.81 (450.41-733.51) | 928.94 (696.37-1228.08) |
| 2017 | 19953105 (15447669,25541820) | 33031171 (24273704,43998818) | 553.91 (430.93-706.67) | 907.58 (673.01-1205.5) |
| 2018 | 19255684 (14788008,24809432) | 32530092 (23807057,43552413) | 531.92 (411.31-682.53) | 886.37 (653-1183.84) |
| 2019 | 18612152 (14278587,24094047) | 32112307 (23226076,43200585) | 512.71 (395.97-660.57) | 868.66 (631.72-1165.38) |
| 2020 | 18115906 (13898159,23573710) | 31617395 (22764638,42773243) | 498.11 (383.69-644.8) | 848.58 (612.18-1144.53) |
| 2021 | 17624433 (13425595,23103361) | 31294828 (22374975,42368402) | 484.62 (372-631.78) | 834.92 (598.63-1127.32) |
| Percent change between 1990 and 2021 | -0.49 (-0.56,-0.41) | -0.29 (-0.41,-0.19) | -0.59 (-0.64,-0.53) | -0.47 (-0.55,-0.4) |

## Table S10: Incidence of Nutritional Deficiencies in cases and and Age-Standardized Rates in 1990 and 2021 by Sex and location: Trends and EAPC Analysis

| **Incidence** | | | | | | | | | | |
| --- | --- | --- | --- | --- | --- | --- | --- | --- | --- | --- |
|  | **1990** | | | | **2021** | | | | **male** | **female** |
| Location | **All-ages cases (Male)** | **All-ages cases (Female)** | **Age-standardized Rates(M) (per 100,000)** | **Age-standardized Rates(F) (per 100,000)** | **All-ages cases (Male)** | **All-ages cases (Female)** | **Age-standardized Rates(M) (per 100,000)** | **Age-standardized Rates(F) (per 100,000)** | **1990-2021 EAPC** | **1990-2021 EAPC** |
|  | **n (95% UI)** | **n (95% UI)** | **n (95% UI)** | **n (95% UI)** | **n (95% UI)** | **n (95% UI)** | **n (95% UI)** | **n (95% UI)** | **n (95% UI)** | **n (95% UI)** |
| Global | 574970672 (546730045-605735858) | 389911024 (371980319-408135288) | 20170.14 (19158.18-21173.1) | 14000.41 (13368.81-14655.15) | 323658105 (306984148-343250270) | 262471282 (250683006-276642939) | 8423.99 (7987.09-8934.09) | 7006.41 (6700.12-7383.65) | -2.76 (-2.9--2.61) | -2.22 (-2.37--2.06) |
| SDI | | | | | | | | | | |
| High SDI | 10921373 (9757462-12146114) | 13624753 (12511115-14884842) | 2639.24 (2373.24-2940.67) | 3174.6 (2911.02-3469.21) | 9153382 (7963355-10552305) | 10421658 (9224145-11959960) | 1562.81 (1347.15-1802.03) | 1786.26 (1581.15-2017.52) | -1.26 (-1.46--1.07) | -1.55 (-1.68--1.42) |
| High-middle SDI | 49228705 (44424306-54388009) | 38559424 (35576053-41816759) | 9254.26 (8382.44-10205.59) | 7377.83 (6813.37-7988.02) | 20313447 (18409186-22462408) | 21163237 (19378351-23180040) | 3221.87 (2926.02-3556.7) | 3396.33 (3118.19-3685.58) | -3.22 (-3.32--3.12) | -2.47 (-2.58--2.36) |
| Middle SDI | 162063093 (150356694-174869440) | 107664061 (100433554-115044130) | 17131 (15933.72-18379.29) | 11889.89 (11125.75-12658.08) | 60485345 (56134631-65383232) | 56826642 (53147154-61271455) | 5107.07 (4732.07-5505.54) | 4925.51 (4607.65-5319.15) | -3.69 (-3.79--3.59) | -2.76 (-2.86--2.65) |
| Low-middle SDI | 214239387 (199228461-228218718) | 137755787 (129687043-146219294) | 33743.01 (31348.68-35958.34) | 21936.46 (20718.04-23348.06) | 101156248 (92457632-110402953) | 80486431 (75090007-86747829) | 10393.94 (9546.36-11289.67) | 8377.55 (7843.33-8995.61) | -3.76 (-3.93--3.59) | -3.06 (-3.21--2.92) |
| Low SDI | 138103010 (133298629-142933885) | 91898047 (88375339-95047146) | 51793.58 (50032.68-53504.49) | 33293.44 (32137.64-34375.7) | 132321885 (126616208-138381905) | 93329642 (89247323-97553320) | 22574.11 (21748.15-23527.73) | 15558.78 (14943.98-16148.81) | -2.75 (-2.97--2.53) | -2.53 (-2.75--2.31) |
| Region | | | | | | | | | | |
| High-income Asia Pacific | 1641462 (1397003-1921427) | 2053133 (1812418-2330138) | 2088.49 (1781.42-2413.88) | 2578 (2259.87-2918.81) | 1219969 (1053761-1417661) | 1326401 (1154822-1543852) | 1305.21 (1136.1-1517.87) | 1470.36 (1274.28-1708.73) | -1.37 (-1.49--1.26) | -1.53 (-1.67--1.4) |
| High-income North America | 2197641 (1790461-2772850) | 3180449 (2709539-3769733) | 1616.42 (1320.86-2024.45) | 2250.7 (1922.46-2634.69) | 3125408 (2505428-3779921) | 2746285 (2313779-3368355) | 1520.54 (1209.68-1842.61) | 1377.16 (1172.96-1652.58) | -0.01 (-0.38-0.37) | -1.46 (-1.67--1.24) |
| Western Europe | 4514645 (4126651-5032567) | 4260380 (3877369-4665453) | 2530.13 (2300.32-2834.13) | 2252.11 (2050.78-2471.39) | 3785635 (3381489-4257790) | 4494135 (3746639-5524713) | 1603.43 (1427.2-1811.24) | 1756.57 (1477.35-2104.67) | -0.92 (-1.11--0.73) | -0.1 (-0.33-0.13) |
| Australasia | 91351 (76345-110517) | 98138 (83380-115695) | 902.26 (754.27-1087.51) | 929.32 (788-1101.92) | 141363 (120343-167376) | 159661 (140316-188701) | 817.19 (696.81-969.91) | 872.8 (758.23-1031.11) | -0.03 (-0.23-0.18) | 0.08 (-0.13-0.29) |
| Andean Latin America | 2244989 (1966231-2562611) | 2079062 (1859027-2312489) | 10501.95 (9215.4-11890.14) | 10161.45 (9159.13-11217.7) | 1546481 (1349615-1813783) | 1754091 (1563095-1961532) | 4627 (4053.81-5412.11) | 5313.28 (4750.79-5935.9) | -3.07 (-3.36--2.78) | -2.33 (-2.52--2.14) |
| Tropical Latin America | 20311799 (17747806-23225929) | 16167651 (14230005-18484999) | 26495.68 (23136.02-30215.67) | 20574.89 (18080.4-23307.74) | 11967438 (10097022-14069061) | 11019530 (9337658-12953609) | 10770.25 (9085.75-12728.53) | 9567.75 (8113.69-11228.08) | -3.03 (-3.16--2.91) | -2.48 (-2.53--2.43) |
| Central Latin America | 13685851 (12314593-15158557) | 9516193 (8713424-10358695) | 15390.83 (13890.55-17003.16) | 10693.52 (9937.09-11522.49) | 7547456 (6666963-8584117) | 6393504 (5852222-7023264) | 6168.25 (5448.83-7002.88) | 5045.54 (4620.13-5561.59) | -2.82 (-2.9--2.74) | -2.2 (-2.3--2.1) |
| Southern Latin America | 3130466 (2721512-3587326) | 2403968 (2104922-2716807) | 12587.08 (10985.1-14453.15) | 9487.25 (8306.42-10713.72) | 2173264 (1850416-2527285) | 1978430 (1721641-2295273) | 6749.98 (5726.82-7828.35) | 5898.21 (5125.18-6935.75) | -1.89 (-2.17--1.61) | -1.41 (-1.62--1.21) |
| Caribbean | 2321220 (2115062-2501659) | 1938902 (1784580-2092628) | 12777.76 (11720.85-13720.68) | 10519.8 (9727.71-11310.6) | 1535597 (1356354-1763620) | 1498167 (1332313-1667143) | 6713.81 (5912.2-7692.8) | 6464.89 (5737.53-7235.7) | -2.28 (-2.36--2.21) | -1.69 (-1.73--1.66) |
| Central Europe | 8968349 (8356720-9624330) | 12076006 (11355415-12928307) | 15141.03 (14117.36-16213.79) | 19215.22 (18051.28-20571.51) | 3027677 (2815807-3257775) | 5293735 (4940742-5659467) | 5904.28 (5470.05-6364.5) | 9018.89 (8438.31-9606.92) | -3.21 (-3.35--3.06) | -2.5 (-2.57--2.42) |
| Eastern Europe | 1856040 (1575293-2150833) | 2201701 (1943142-2474781) | 1832.56 (1558.96-2130.97) | 1935.71 (1691.83-2199.81) | 985549 (801400-1189218) | 1185546 (1021301-1365351) | 1141.77 (927.01-1371.03) | 1142.4 (966.57-1343.92) | -1.19 (-1.35--1.04) | -1.32 (-1.48--1.15) |
| Central Asia | 3855201 (3466886-4251010) | 2643043 (2399759-2877710) | 10814.34 (9816.97-11830.91) | 7089.62 (6508.97-7710.51) | 2633730 (2404677-2916020) | 2115998 (1948169-2312175) | 5477.36 (5004.31-6075.6) | 4387.11 (4035.61-4780.78) | -2.22 (-2.37--2.06) | -1.5 (-1.64--1.37) |
| North Africa and Middle East | 30034368 (28538855-31732860) | 21605483 (20529573-22767028) | 16155.77 (15385.26-16978.15) | 12345.1 (11719.81-12940.8) | 19360806 (18004145-20737308) | 14731101 (13902602-15721571) | 6001.37 (5596.6-6427.57) | 4923.16 (4645.91-5243.9) | -3.02 (-3.19--2.85) | -2.84 (-2.95--2.72) |
| South Asia | 207633634 (185705540-229630541) | 124758509 (113666806-136973228) | 34474.65 (30819.11-38030.62) | 21570.66 (19665.03-23702.31) | 94881989 (81950536-108911760) | 71300003 (63481860-81387234) | 10279.45 (8890.38-11743.71) | 8028.39 (7150.41-9146.65) | -3.85 (-4.1--3.59) | -3.14 (-3.37--2.92) |
| Southeast Asia | 57256090 (52535804-61998914) | 46613950 (43367806-49952437) | 21666.01 (19888.02-23396.39) | 18094.73 (16928.5-19360.31) | 19058232 (17228595-21171611) | 19854704 (18272772-21724841) | 5758.05 (5204.8-6395.87) | 6029.16 (5543.32-6625.74) | -4.04 (-4.15--3.92) | -3.43 (-3.49--3.36) |
| East Asia | 92313704 (78058575-108498053) | 53414541 (45726898-61946684) | 14054.99 (11984.73-16351.77) | 8803.11 (7591.48-10159.84) | 24412567 (21251031-28108403) | 23541230 (20739437-26857137) | 3386.46 (2921.11-3965.86) | 3528.9 (3063.18-4042.38) | -4.27 (-4.43--4.11) | -2.94 (-3.09--2.79) |
| Oceania | 773620 (703154-848072) | 565758 (511250-620087) | 20442.61 (18754.08-22310.87) | 16302.7 (14912.34-17763.52) | 857851 (754954-974019) | 728491 (640814-823578) | 10750.55 (9503.12-12047.9) | 9879.93 (8768.03-11087.65) | -1.69 (-1.86--1.53) | -1.31 (-1.44--1.18) |
| Central Sub-Saharan Africa | 14155352 (13109599-15356678) | 8605974 (7718104-9502438) | 47664.9 (44197.77-51287.59) | 26465.34 (23926.18-29074.15) | 21624576 (19134570-24057308) | 12751986 (11296600-14570513) | 29818.81 (26612.74-32931.14) | 16539.1 (14703.88-18662.58) | -1.4 (-1.83--0.96) | -1.43 (-1.86--1.01) |
| Eastern Sub-Saharan Africa | 54542776 (52701233-56384045) | 42241589 (40603044-43818015) | 57148.69 (55519.47-58940.23) | 42077.41 (40665.46-43506.5) | 51145036 (48529866-54013456) | 42820395 (40689683-44910868) | 24262.74 (23149.26-25408.13) | 19495.85 (18691.42-20344.5) | -2.95 (-3.16--2.73) | -2.63 (-2.81--2.46) |
| Southern Sub-Saharan Africa | 5424247 (4886301-6063578) | 4394457 (3934311-4899302) | 17282.85 (15605.77-19164.16) | 14031.11 (12709.8-15487.36) | 3009586 (2623750-3453285) | 3186432 (2848033-3563279) | 7261.15 (6373.12-8305.29) | 7637.4 (6849.28-8503.42) | -2.64 (-2.74--2.54) | -1.75 (-1.85--1.65) |
| Western Sub-Saharan Africa | 48017866 (46381108-49752211) | 29092138 (27817027-30480484) | 46268.36 (44732.63-47845.22) | 25795.28 (24706.98-26875.26) | 49617894 (47210358-52048311) | 33591460 (31699300-35790105) | 19071.71 (18231.31-19867.38) | 11964.95 (11320.39-12637.11) | -2.84 (-2.86--2.81) | -2.46 (-2.65--2.26) |

## Table S11: Prevalence of Nutritional Deficiencies in cases and and Age-Standardized Rates in 1990 and 2021 by Sex and location: Trends and EAPC Analysis

| **Prevalence** | | | | | | | | | | |
| --- | --- | --- | --- | --- | --- | --- | --- | --- | --- | --- |
|  | **1990** | | | | **2021** | | | | **male** | **female** |
| Location | **All-ages cases (Male)** | **All-ages cases (Female)** | **Age-standardized Rates(M) (per 100,000)** | **Age-standardized Rates(F) (per 100,000)** | **All-ages cases (Male)** | **All-ages cases (Female)** | **Age-standardized Rates(M) (per 100,000)** | **Age-standardized Rates(F) (per 100,000)** | **1990-2021 EAPC** | **1990-2021 EAPC** |
|  | **n (95% UI)** | **n (95% UI)** | **n (95% UI)** | **n (95% UI)** | **n (95% UI)** | **n (95% UI)** | **n (95% UI)** | **n (95% UI)** | **n (95% UI)** | **n (95% UI)** |
| Global | 863913113 (842154181-887514594) | 901197651 (887274921-917398451) | 31249.65 (30494.23-32050.1) | 33315.43 (32809.58-33919.87) | 753569459 (735303455-772046911) | 1091677099 (1071437522-1113428733) | 19598.06 (19144.93-20066.79) | 28247.48 (27750.73-28804.58) | -1.5 (-1.54--1.46) | -0.55 (-0.56--0.54) |
| SDI | | | | | | | | | | |
| High SDI | 27749647 (26368468-29270700) | 51346582 (48623404-54470956) | 6728.41 (6382.27-7096.08) | 11641.63 (11054.14-12335.44) | 24997106 (23339929-26849850) | 49231231 (46490712-52004970) | 4218.07 (3939.6-4526.47) | 8872.11 (8413.95-9384.91) | -1.26 (-1.36--1.16) | -0.7 (-0.81--0.59) |
| High-middle SDI | 96793887 (91744047-101454558) | 118508705 (114834045-122482312) | 18684.63 (17775.26-19543.16) | 22267.96 (21570.6-23019.47) | 52921588 (50421501-55636964) | 103221276 (99422322-107356341) | 8386.23 (8026.66-8799.5) | 15882.69 (15260.32-16541.39) | -2.64 (-2.7--2.58) | -1.15 (-1.18--1.11) |
| Middle SDI | 261643341 (251296005-273776237) | 273933765 (267238941-280944757) | 29269.54 (28187.37-30495.19) | 31785.36 (31048.5-32541.13) | 165211063 (159438447-171184222) | 293625800 (286737411-300941599) | 14121.3 (13651.57-14618.69) | 24305.08 (23751.31-24881.01) | -2.3 (-2.33--2.27) | -0.87 (-0.89--0.85) |
| Low-middle SDI | 309850505 (299826031-319047651) | 302298073 (296417609-308659083) | 51208.68 (49647.76-52678.43) | 52294.44 (51306.18-53350.56) | 276147173 (267037247-285870742) | 381603146 (373193626-390016197) | 29259.24 (28354.47-30231.37) | 39979.99 (39148.58-40835.25) | -1.81 (-1.84--1.77) | -0.88 (-0.89--0.87) |
| Low SDI | 167196313 (163867734-170615440) | 154262339 (151910748-156772865) | 64798.16 (63619.19-65996.75) | 61015.68 (60087.35-62003.23) | 233773471 (227320031-240585712) | 263177795 (257235534-268835742) | 41096.51 (39993.32-42151.75) | 47235.88 (46165.31-48293.46) | -1.52 (-1.63--1.41) | -0.89 (-0.93--0.85) |
| Region | | | | | | | | | | |
| High-income Asia Pacific | 4756064 (4174375-5553473) | 10417351 (8892295-12436271) | 6244.41 (5477.04-7489.46) | 11782.6 (10166.27-13953.81) | 4241513 (3653080-5146925) | 8274201 (7108981-9837664) | 3780.43 (3190.65-4748.79) | 8305.29 (7096.45-10084.88) | -1.5 (-1.63--1.37) | -0.97 (-1.13--0.81) |
| High-income North America | 5058632 (4582419-5673867) | 11239882 (10250187-12310923) | 3767.03 (3415.99-4219.66) | 7620.77 (6976.16-8327.52) | 7156169 (6245665-8077588) | 13157133 (11916801-14724684) | 3451.53 (3003.31-3958.41) | 6842.38 (6203.73-7695.91) | -0.03 (-0.17-0.12) | -0.14 (-0.26--0.02) |
| Western Europe | 10871001 (10239511-11601132) | 20454134 (18751185-22851958) | 5882.87 (5520.91-6341.69) | 10903.74 (10030.83-12108.55) | 9351150 (8679911-10156692) | 18412949 (17028207-20481896) | 3763.41 (3473.6-4127.67) | 8311.05 (7679.05-9288.05) | -1.21 (-1.33--1.09) | -0.72 (-0.84--0.61) |
| Australasia | 440726 (324088-723830) | 718051 (645007-813028) | 4899.69 (3458.66-8444.53) | 6938.09 (6192.61-7994.83) | 520965 (415510-763389) | 938633 (850250-1040353) | 3345.02 (2428.57-5866.29) | 5857.06 (5241.49-6625.44) | -1.16 (-1.25--1.08) | -0.47 (-0.53--0.41) |
| Andean Latin America | 4630677 (4093776-5220060) | 5853438 (5355976-6417246) | 21716.99 (19285.77-24340.9) | 29592.95 (27201.14-32309.73) | 3690401 (3112766-4437285) | 6070189 (5707043-6533577) | 11289.27 (9622.01-13447.83) | 18309.16 (17248.23-19692.64) | -2.35 (-2.46--2.24) | -1.73 (-1.83--1.63) |
| Tropical Latin America | 26086920 (23399096-29121691) | 32639477 (30415461-35178263) | 34151.02 (30845.28-37615.45) | 41740.54 (38893.37-44791.24) | 17382000 (15161728-20228220) | 34464394 (31585408-37752198) | 16155.05 (14074.92-18600.94) | 29685.89 (27306.68-32296.72) | -2.52 (-2.59--2.46) | -1.11 (-1.12--1.1) |
| Central Latin America | 19368444 (18007612-20726472) | 18176089 (17268609-19157038) | 21994.3 (20545.74-23451.37) | 20770.3 (19860.4-21736.85) | 13221295 (12305554-14188379) | 18162575 (17419997-19002147) | 11132.01 (10393.51-11924.63) | 14210.91 (13627.2-14835.15) | -2.12 (-2.15--2.09) | -1.14 (-1.17--1.11) |
| Southern Latin America | 4879082 (4274560-5762434) | 4508521 (3828806-5579774) | 20030.89 (17656.93-23401.55) | 17933.97 (15216.34-22182.3) | 3649990 (3092982-4428777) | 4340918 (3350587-5753032) | 11742.47 (9729.64-14815.78) | 13184.18 (10097.2-17714.11) | -1.62 (-1.73--1.5) | -0.92 (-0.96--0.87) |
| Caribbean | 4349440 (4130176-4603095) | 6028121 (5793379-6295355) | 24246.79 (23033.72-25577.71) | 32912.49 (31637.32-34205.07) | 4213444 (3911049-4575985) | 7176060 (6848677-7528777) | 18647.72 (17261.94-20270.73) | 30701.09 (29290.55-32247.66) | -0.9 (-1--0.8) | -0.26 (-0.34--0.19) |
| Central Europe | 16181415 (15328647-17109900) | 20543636 (19820478-21398393) | 27286.9 (25908.83-28804.06) | 32823.93 (31631.79-34267.6) | 7053740 (6645801-7566936) | 12170981 (11719499-12732085) | 13675.28 (12823.83-14704.1) | 21169.98 (20341.79-22179.8) | -2.36 (-2.43--2.3) | -1.47 (-1.5--1.44) |
| Eastern Europe | 11448359 (10449217-12669015) | 21219187 (19717997-22963326) | 11317.62 (10350-12384.32) | 17844.28 (16657.64-19201.5) | 7213914 (6412420-8209471) | 16062157 (14817366-17617750) | 7713.06 (6887.33-8752.1) | 14585.66 (13597.69-15771.12) | -1.42 (-1.59--1.24) | -0.76 (-0.85--0.67) |
| Central Asia | 9392318 (8818299-10014411) | 12941922 (12385826-13586964) | 25959.68 (24517.73-27483.63) | 35881.19 (34401.54-37537.93) | 8649839 (8071824-9542646) | 15154358 (14406331-16132250) | 18155.95 (17030.2-19923.04) | 31024.78 (29463.75-32951.36) | -1.27 (-1.37--1.17) | -0.57 (-0.62--0.52) |
| North Africa and Middle East | 52867138 (51328220-54727377) | 57543254 (55769752-59423752) | 28881.21 (28007.59-29852.57) | 34314.88 (33279.25-35351.41) | 48323029 (46599353-50288855) | 72649840 (70364176-75079416) | 15341.46 (14819.68-15939.04) | 24094.49 (23356.04-24920.93) | -1.96 (-2.01--1.92) | -1.1 (-1.11--1.08) |
| South Asia | 320195025 (305870116-333914722) | 313909775 (306268339-321613656) | 55916.65 (53637.42-58266.79) | 59620.08 (58255.77-61059.09) | 304318850 (292718109-317903641) | 434732911 (423427591-446154145) | 33797.46 (32590.68-35211.9) | 48162.69 (46976.7-49365.26) | -1.63 (-1.68--1.59) | -0.7 (-0.71--0.69) |
| Southeast Asia | 79433996 (75187483-83790681) | 92669482 (89696249-96066244) | 32749.4 (31035.81-34299.51) | 38766.09 (37623.85-40115.94) | 48233327 (45300695-51282697) | 89362075 (85871204-92995178) | 14885.31 (14021.15-15774.97) | 25723.61 (24729.14-26723.12) | -2.5 (-2.58--2.41) | -1.35 (-1.41--1.28) |
| East Asia | 147019353 (134007617-162272670) | 134113874 (126525547-142046643) | 23658.14 (21803.81-25852.59) | 22291.52 (21082.74-23548.63) | 55201796 (51529869-59721112) | 97062823 (92150468-102243807) | 7306.23 (6798.61-7920.2) | 12922.54 (12277.94-13613.57) | -3.77 (-3.85--3.69) | -1.84 (-1.9--1.78) |
| Oceania | 1151352 (1066452-1241504) | 1400462 (1293630-1539286) | 32267.25 (30287.04-34435.93) | 43497.43 (40347.5-47168.76) | 1791892 (1613607-2038920) | 2456721 (2167702-2849421) | 24062.04 (21964.43-27052.28) | 35542.72 (31632.08-40521.26) | -0.68 (-0.78--0.58) | -0.61 (-0.69--0.52) |
| Central Sub-Saharan Africa | 17455581 (16571411-18348606) | 16969169 (16193957-17738857) | 62028.43 (59263.84-64891.08) | 60783.44 (58540.21-63024.02) | 30388459 (28157298-32555395) | 31524807 (29603704-33769344) | 43600.87 (40874.23-46150.03) | 46062.09 (43483.46-49331.73) | -1.04 (-1.33--0.76) | -0.83 (-1.01--0.65) |
| Eastern Sub-Saharan Africa | 62882061 (61247059-64445904) | 58331588 (57007659-59708602) | 65821.3 (64360.88-67280.62) | 60076.04 (58849.45-61361.68) | 82035642 (78775248-85575366) | 88697918 (86190992-91422546) | 38468.11 (37264.88-39787.36) | 41319.7 (40227.16-42451.84) | -1.85 (-1.97--1.72) | -1.32 (-1.38--1.26) |
| Southern Sub-Saharan Africa | 8761777 (8170367-9354818) | 10363902 (9771085-10986319) | 30878.28 (28990.69-32894.65) | 36042.28 (34103.53-38166.27) | 8039081 (7511435-8584759) | 11696597 (11087199-12340638) | 20296.43 (19063.98-21526) | 28079.65 (26665.08-29559.79) | -1.27 (-1.32--1.22) | -0.78 (-0.82--0.75) |
| Western Sub-Saharan Africa | 56683752 (55045514-58476565) | 51156336 (49620694-52912649) | 55637.23 (54084.08-57412.93) | 49855.31 (48352.17-51432.57) | 88892965 (84434986-93651400) | 109108858 (105425017-113156376) | 34081.15 (32669.52-35659.81) | 42037.42 (40836.83-43354.55) | -1.56 (-1.58--1.55) | -0.58 (-0.6--0.55) |

## Table S12: Deaths of Nutritional Deficiencies in cases and and Age-Standardized Rates in 1990 and 2021 by Sex and location: Trends and EAPC Analysis

| **Deaths** | | | | | | | | | | |
| --- | --- | --- | --- | --- | --- | --- | --- | --- | --- | --- |
|  | **1990** | | | | **2021** | | | | **male** | **female** |
| Location | **All-ages cases (Male)** | **All-ages cases (Female)** | **Age-standardized Rates(M) (per 100,000)** | **Age-standardized Rates(F) (per 100,000)** | **All-ages cases (Male)** | **All-ages cases (Female)** | **Age-standardized Rates(M) (per 100,000)** | **Age-standardized Rates(F) (per 100,000)** | **1990-2021 EAPC** | **1990-2021 EAPC** |
|  | **n (95% UI)** | **n (95% UI)** | **n (95% UI)** | **n (95% UI)** | **n (95% UI)** | **n (95% UI)** | **n (95% UI)** | **n (95% UI)** | **n (95% UI)** | **n (95% UI)** |
| Global | 264756 (220967-320959) | 305363 (251850-370281) | 10.71 (9.28-12.56) | 11.4 (9.51-13.61) | 105634 (93230-120200) | 116640 (103335-129658) | 3.16 (2.8-3.58) | 2.96 (2.63-3.33) | -4.26 (-4.69--3.83) | -4.57 (-4.98--4.15) |
| SDI | | | | | | | | | | |
| High SDI | 3172 (2979-3326) | 4625 (4015-4979) | 0.88 (0.82-0.93) | 0.72 (0.63-0.78) | 9659 (8575-10254) | 14878 (11699-16601) | 1.01 (0.9-1.07) | 0.9 (0.73-0.99) | 0.03 (-0.43-0.48) | 0.3 (-0.24-0.83) |
| High-middle SDI | 7915 (7176-8790) | 9225 (8173-10359) | 2.23 (2.04-2.46) | 2.05 (1.8-2.31) | 6770 (5927-7681) | 6911 (5779-7831) | 1.05 (0.91-1.18) | 0.66 (0.57-0.75) | -2.63 (-2.9--2.37) | -3.87 (-4.22--3.51) |
| Middle SDI | 52327 (47278-57720) | 57245 (52405-62717) | 10.38 (9.37-11.13) | 9.7 (8.84-10.47) | 26292 (24026-28390) | 27251 (23906-30187) | 2.95 (2.67-3.18) | 2.39 (2.09-2.65) | -4.04 (-4.09--3.99) | -4.45 (-4.53--4.38) |
| Low-middle SDI | 99673 (82371-119503) | 128321 (103232-156396) | 16.7 (14.11-19.35) | 22.56 (18.75-27.02) | 24067 (21202-27487) | 28167 (24671-32317) | 3.61 (3.24-4.02) | 3.86 (3.4-4.39) | -5.7 (-6.35--5.04) | -6.32 (-7--5.64) |
| Low SDI | 101512 (79740-133919) | 105793 (79758-137577) | 33.76 (27.24-41.71) | 36.25 (29.07-44.85) | 38755 (29711-48980) | 39345 (31879-47355) | 8.55 (7.07-10.18) | 8.88 (7.67-10.25) | -4.31 (-4.97--3.65) | -4.4 (-4.99--3.81) |
| Region | | | | | | | | | | |
| High-income Asia Pacific | 503 (474-530) | 572 (502-629) | 0.71 (0.65-0.75) | 0.58 (0.5-0.63) | 1418 (1266-1505) | 1025 (743-1200) | 0.66 (0.6-0.69) | 0.27 (0.22-0.3) | -0.52 (-0.84--0.19) | -2.32 (-2.72--1.92) |
| High-income North America | 1060 (982-1106) | 1309 (1104-1413) | 0.84 (0.76-0.88) | 0.56 (0.48-0.6) | 4987 (4411-5328) | 8515 (6849-9420) | 1.73 (1.53-1.86) | 1.88 (1.55-2.05) | 1.6 (0.68-2.53) | 3.08 (2.23-3.94) |
| Western Europe | 1242 (1153-1319) | 2340 (2010-2558) | 0.7 (0.64-0.75) | 0.63 (0.54-0.69) | 3168 (2731-3461) | 5452 (4225-6232) | 0.71 (0.61-0.77) | 0.62 (0.49-0.7) | 0.08 (-0.13-0.29) | -0.04 (-0.22-0.13) |
| Australasia | 44 (41-47) | 41 (35-45) | 0.56 (0.51-0.61) | 0.31 (0.27-0.34) | 70 (62-77) | 94 (74-107) | 0.27 (0.24-0.3) | 0.25 (0.2-0.28) | -2.27 (-2.53--2.01) | -1.22 (-1.61--0.83) |
| Andean Latin America | 3375 (2776-4055) | 3523 (2998-4105) | 21.13 (18.09-24.57) | 22.45 (19.54-25.36) | 1410 (1134-1764) | 1575 (1281-1961) | 5.31 (4.28-6.63) | 5.15 (4.19-6.42) | -4.75 (-5.01--4.49) | -5.12 (-5.37--4.87) |
| Tropical Latin America | 7058 (6501-7675) | 5719 (5243-6189) | 12.67 (11.91-13.53) | 9.58 (8.82-10.27) | 3415 (3104-3634) | 2809 (2380-3089) | 3.42 (3.08-3.66) | 2.04 (1.74-2.24) | -4.29 (-4.57--4) | -5.06 (-5.36--4.76) |
| Central Latin America | 12958 (12397-13570) | 12611 (12075-13164) | 26.13 (25.03-27) | 25.07 (23.79-25.9) | 5921 (5222-6712) | 5460 (4769-6118) | 5.72 (5.06-6.48) | 4.3 (3.75-4.86) | -5.06 (-5.17--4.96) | -5.77 (-5.88--5.66) |
| Southern Latin America | 1093 (1039-1151) | 851 (800-902) | 5.42 (5.13-5.7) | 3.47 (3.26-3.67) | 681 (617-738) | 810 (687-893) | 1.97 (1.78-2.14) | 1.49 (1.29-1.63) | -3.16 (-3.76--2.56) | -2.74 (-3.41--2.06) |
| Caribbean | 2017 (1604-2468) | 1984 (1585-2529) | 11.3 (9.28-13.48) | 10.97 (9.03-13.64) | 1033 (797-1375) | 848 (682-1072) | 4.73 (3.6-6.38) | 3.6 (2.79-4.68) | -2.72 (-3.1--2.34) | -3.37 (-3.76--2.98) |
| Central Europe | 90 (84-98) | 104 (96-112) | 0.17 (0.16-0.19) | 0.16 (0.15-0.18) | 354 (323-383) | 393 (349-426) | 0.43 (0.39-0.47) | 0.3 (0.27-0.33) | 2.31 (1.73-2.89) | 1.5 (0.88-2.11) |
| Eastern Europe | 533 (512-554) | 573 (551-596) | 0.54 (0.52-0.56) | 0.43 (0.41-0.45) | 369 (335-401) | 509 (464-552) | 0.3 (0.28-0.32) | 0.27 (0.25-0.29) | -3.53 (-4.52--2.53) | -2.72 (-3.74--1.7) |
| Central Asia | 459 (417-507) | #N/A | 1.27 (1.17-1.37) | 1.18 (1.09-1.28) | 129 (110-152) | 146 (128-166) | 0.3 (0.26-0.35) | 0.32 (0.28-0.36) | -5.74 (-6.18--5.3) | -4.74 (-5.16--4.32) |
| North Africa and Middle East | 5943 (4358-8994) | 8223 (6431-10945) | 3.36 (2.7-4.54) | 4.77 (3.91-5.99) | 2243 (1845-2704) | 3033 (2522-3650) | 1.08 (0.91-1.29) | 1.48 (1.26-1.75) | -3.84 (-3.97--3.7) | -3.9 (-4.1--3.7) |
| South Asia | #N/A | 128677 (100602-157671) | 14.56 (11.93-17.26) | 24.76 (19.82-29.85) | 13271 (11125-16076) | 21958 (18068-26203) | 2.01 (1.71-2.42) | 3.1 (2.56-3.69) | -6.18 (-6.34--6.01) | -6.31 (-6.49--6.13) |
| Southeast Asia | 22046 (17990-26475) | 19114 (15214-23579) | 18.42 (15.11-21.08) | 12.8 (10.25-15.11) | 13962 (11897-15867) | 15056 (12597-17646) | 6.77 (5.77-7.64) | 5.41 (4.53-6.3) | -3.06 (-3.17--2.96) | -2.55 (-2.74--2.35) |
| East Asia | 16319 (13963-19068) | 21843 (18772-25305) | 5.27 (4.68-5.94) | 5.82 (5.03-6.65) | 8842 (7364-10688) | 7610 (5813-9483) | 1.62 (1.37-1.94) | 0.86 (0.66-1.06) | -6.5 (-8.42--4.53) | -9.33 (-11.44--7.18) |
| Oceania | 116 (89-148) | 122 (95-156) | 7.16 (5.86-8.75) | 7.85 (6.46-9.52) | 151 (114-202) | 141 (106-186) | 4.49 (3.46-5.69) | 4.23 (3.4-5.26) | -1.48 (-1.52--1.44) | -2.11 (-2.17--2.05) |
| Central Sub-Saharan Africa | 12922 (9449-19084) | 11065 (7470-16443) | 39.06 (30.26-52.65) | 31.62 (23.64-42.55) | 5049 (3324-7232) | 4165 (2812-5861) | 11.75 (8.27-15.78) | 8.88 (6.34-12.46) | -4.12 (-4.46--3.77) | -4.32 (-4.66--3.97) |
| Eastern Sub-Saharan Africa | 61305 (48148-81480) | 57993 (43940-75704) | 61.1 (49.6-74.39) | 55.46 (45.07-67.33) | 21903 (17102-27369) | 20456 (16550-24786) | 15.2 (12.88-17.99) | 13.1 (11.11-15.41) | -4.42 (-5.3--3.52) | -4.58 (-5.47--3.69) |
| Southern Sub-Saharan Africa | 3936 (3268-4994) | 3513 (2938-4113) | 15.01 (13.03-17.86) | 11.58 (9.91-13.35) | 2990 (2382-3707) | 2753 (2188-3340) | 9.4 (7.81-11.33) | 7.57 (6.12-9.12) | -1.12 (-1.33--0.9) | -0.42 (-0.76--0.08) |
| Western Sub-Saharan Africa | 25444 (19173-33961) | 24747 (18140-34955) | 19.02 (15.05-23.99) | 19 (15.08-25.06) | 14269 (9725-19404) | 13832 (10179-17488) | 6.27 (4.86-7.91) | 6.52 (5.31-7.87) | -3.52 (-3.64--3.39) | -3.35 (-3.47--3.23) |

## Table S13: DALYs of Nutritional Deficiencies in cases and and Age-Standardized Rates in 1990 and 2021 by Sex and location: Trends and EAPC Analysis

| **DALYs** | | | | | | | | | | |
| --- | --- | --- | --- | --- | --- | --- | --- | --- | --- | --- |
|  | **1990** | | | | **2021** | | | | **male** | **female** |
| Location | **All-ages cases (Male)** | **All-ages cases (Female)** | **Age-standardized Rates(M) (per 100,000)** | **Age-standardized Rates(F) (per 100,000)** | **All-ages cases (Male)** | **All-ages cases (Female)** | **Age-standardized Rates(M) (per 100,000)** | **Age-standardized Rates(F) (per 100,000)** | **1990-2021 EAPC** | **1990-2021 EAPC** |
|  | **n (95% UI)** | **n (95% UI)** | **n (95% UI)** | **n (95% UI)** | **n (95% UI)** | **n (95% UI)** | **n (95% UI)** | **n (95% UI)** | **n (95% UI)** | **n (95% UI)** |
| Global | 34427040 (28554310-41851272) | 44247184 (36250406-55715793) | 1171.63 (967.32-1430.87) | 1573.03 (1282.74-1994.48) | 17624433 (13425595-23103361) | 31294828 (22374975-42368402) | 484.62 (372-631.78) | 834.92 (598.63-1127.32) | -3.03 (-3.28--2.77) | -2.17 (-2.33--2.02) |
| SDI | | | | | | | | | | |
| High SDI | 365473 (255319-520545) | 743733 (521912-1102824) | 94.05 (66.25-131.85) | 169.95 (118.34-253.4) | 534055 (405813-719467) | 983864 (700923-1377053) | 81.78 (59.94-112.2) | 157.08 (107.67-224.47) | 0.01 (-0.18-0.2) | -0.1 (-0.23-0.03) |
| High-middle SDI | 1609388 (1240415-2115217) | 2707544 (2003121-3703383) | 333.74 (259.91-436.38) | 529.04 (396.97-715.75) | 642014 (460944-899810) | 1949988 (1339631-2770612) | 111.72 (80.16-157.49) | 300.37 (206.35-429.08) | -3.85 (-4--3.7) | -2.01 (-2.08--1.93) |
| Middle SDI | 7235027 (6005796-9060787) | 9591974 (7664189-12316547) | 824.99 (690.75-1024.07) | 1129.39 (905.29-1442.84) | 3165361 (2376600-4187178) | 7347659 (5192045-10162279) | 296.43 (224.66-387.48) | 627.36 (444.03-862.57) | -3.27 (-3.3--3.24) | -1.86 (-1.9--1.83) |
| Low-middle SDI | 14097731 (11618171-17413069) | 18649393 (14951137-23228619) | 1945.67 (1588.73-2429.18) | 2816.93 (2253.74-3557.33) | 6433127 (4744632-8610873) | 11661748 (8308193-15904747) | 698.69 (519.3-928.48) | 1243.57 (890.66-1684.79) | -3.66 (-3.96--3.35) | -2.87 (-3.07--2.67) |
| Low SDI | 11099583 (9006417-14079500) | 12528651 (10076818-15851864) | 2961.89 (2444.33-3683.05) | 3704.36 (3003.38-4627.97) | 6838636 (5346791-8880506) | 9331913 (7021047-12256343) | 1053.12 (827.56-1362.76) | 1579.4 (1182.6-2077.13) | -3.3 (-3.72--2.88) | -2.73 (-3.02--2.44) |
| Region | | | | | | | | | | |
| High-income Asia Pacific | 45694 (31752-68584) | 100510 (62830-156262) | 62.5 (42.62-94.98) | 111.43 (70.16-176.42) | 53492 (40179-73941) | 101138 (66489-151380) | 38.53 (27.26-58.05) | 84.17 (52.57-135.11) | -1.51 (-1.59--1.43) | -0.74 (-0.91--0.57) |
| High-income North America | 80312 (45586-162170) | 143471 (95069-230146) | 59.06 (34.23-115.55) | 91.4 (59.52-147.35) | 316809 (227634-433200) | 322760 (234349-452531) | 138.8 (97.34-193.81) | 136.35 (93.65-198.12) | 3.09 (2.5-3.68) | 1.33 (1.2-1.45) |
| Western Europe | 97094 (65428-146594) | 289852 (191937-432697) | 53.05 (35.64-79.55) | 160.35 (103.06-240.17) | 98927 (70307-142137) | 388400 (246968-597464) | 34.89 (22.75-53.16) | 154.98 (95.17-233.31) | -1.28 (-1.48--1.07) | 0.17 (0.02-0.32) |
| Australasia | 4291 (2673-7460) | 9752 (5954-14569) | 48.6 (29.78-87.34) | 91.88 (55.85-137.67) | 4559 (2829-7767) | 13204 (8546-20701) | 28.11 (15.4-52.9) | 78.71 (49.78-127.02) | -1.77 (-1.93--1.6) | -0.5 (-0.61--0.39) |
| Andean Latin America | 300897 (248781-362379) | 324321 (267594-394500) | 1292.48 (1088.24-1533.7) | 1495.16 (1248.93-1803.82) | 87879 (68831-113711) | 149186 (110849-196703) | 286.46 (225.88-367.92) | 463.78 (346.54-609.65) | -5.1 (-5.3--4.89) | -4.03 (-4.22--3.84) |
| Tropical Latin America | 693278 (589535-830476) | 1067672 (844630-1357358) | 903.97 (784.29-1062.88) | 1353.9 (1085.4-1701.23) | 209108 (160757-272993) | 759325 (516799-1078098) | 210.64 (160.25-279.75) | 681.8 (462.4-973.51) | -4.92 (-5.1--4.75) | -2.32 (-2.41--2.24) |
| Central Latin America | 897065 (811794-1009492) | 861684 (772712-976532) | 1050.54 (965.75-1163.35) | 1022.55 (927.22-1146) | 296748 (248631-361162) | 416015 (321203-538042) | 268.46 (226.32-324.96) | 337.42 (263.25-433.98) | -4.38 (-4.49--4.28) | -3.54 (-3.66--3.42) |
| Southern Latin America | 85259 (70848-110480) | 72964 (58980-94115) | 355.24 (296.56-456.63) | 292.71 (236.19-376.98) | 32158 (23366-49098) | 46457 (30343-71660) | 104.73 (74.21-165.19) | 129.76 (83.45-208.4) | -3.84 (-4.07--3.62) | -2.57 (-2.75--2.39) |
| Caribbean | 217584 (172345-270040) | 298123 (235683-376521) | 1089.69 (867.91-1342.16) | 1549.13 (1226.39-1950.79) | 116408 (86883-152545) | 208752 (147615-283349) | 555.16 (409.65-733.15) | 938.12 (669.31-1274.05) | -2.05 (-2.32--1.78) | -1.5 (-1.71--1.29) |
| Central Europe | 159612 (105048-232583) | 269068 (179389-395328) | 287.85 (189.03-418.19) | 432.07 (288.76-638.56) | 61521 (41721-90386) | 162887 (112826-230276) | 127.9 (85.2-189.11) | 275.75 (185.34-398.07) | -2.78 (-2.91--2.64) | -1.6 (-1.66--1.53) |
| Eastern Europe | 213435 (147479-300031) | 560902 (378102-814162) | 218.73 (151.07-306.5) | 482.51 (328.44-702.16) | 101191 (69179-143310) | 380213 (263128-549506) | 114.88 (78.35-163.81) | 355.35 (247.64-522.67) | -2.57 (-2.88--2.26) | -1.26 (-1.4--1.12) |
| Central Asia | 234507 (166669-332912) | 403046 (281419-568899) | 572.42 (407.52-806.43) | 1101.49 (761.31-1550.24) | 156889 (104805-228927) | 414687 (279722-584669) | 321.24 (214.67-469.56) | 846.07 (570.31-1193.67) | -2.31 (-2.49--2.12) | -1.09 (-1.17--1) |
| North Africa and Middle East | 1455471 (1100692-1931635) | 1927837 (1479829-2545505) | 672.73 (508.94-894.83) | 1026.58 (775.98-1375.78) | 904675 (641670-1240982) | 1896761 (1341315-2625964) | 293.59 (210.25-400.86) | 632.64 (449.71-874.5) | -2.7 (-2.78--2.61) | -1.62 (-1.65--1.58) |
| South Asia | 14452122 (11722235-18256951) | 20644676 (16368833-26220013) | 2167.84 (1743.82-2775.31) | 3476.85 (2754.21-4445.48) | 7029479 (5015795-9699297) | 13748782 (9691159-18970719) | 819.66 (590.62-1123.79) | 1567.5 (1114.54-2149.62) | -3.08 (-3.13--3.03) | -2.49 (-2.52--2.46) |
| Southeast Asia | 2342795 (1869041-2926101) | 2800693 (2193094-3640884) | 1005.33 (819.14-1226.78) | 1205.94 (955.93-1550.75) | 1083056 (840569-1399153) | 2172791 (1587231-2959479) | 369.29 (291.96-469.15) | 644.52 (475.64-869.7) | -3.18 (-3.27--3.09) | -2 (-2.09--1.92) |
| East Asia | 2714376 (2165657-3466619) | 3657729 (2916927-4744148) | 489.52 (395.64-618.6) | 661.47 (535.24-846.31) | 628396 (452856-881626) | 1770426 (1190503-2574605) | 94.55 (69.57-128.27) | 233.2 (155.56-336.94) | -7.67 (-9.21--6.1) | -5.22 (-6.42--4) |
| Oceania | 23295 (17208-30524) | 30221 (21178-42068) | 621.25 (470.03-803.01) | 938.9 (663.91-1304.75) | 37117 (25172-55329) | 51041 (33836-75629) | 483.86 (342.36-693.7) | 740.88 (495.88-1078.94) | -0.54 (-0.66--0.42) | -0.54 (-0.63--0.46) |
| Central Sub-Saharan Africa | 1289871 (970661-1800892) | 1246924 (902675-1759362) | 3016.63 (2349.31-3998.03) | 3109.19 (2299.44-4133.54) | 676590 (488564-917832) | 798942 (573204-1110823) | 890.26 (645.78-1186.19) | 1116.62 (797.85-1569.36) | -4.08 (-4.43--3.73) | -3.47 (-3.71--3.23) |
| Eastern Sub-Saharan Africa | 5685039 (4526991-7390952) | 5741944 (4531483-7357781) | 3890.21 (3180.81-4883.55) | 4150.94 (3350.89-5164.13) | 2594030 (2093499-3294127) | 3141249 (2438553-4003208) | 1053.73 (863.79-1313.74) | 1346.84 (1053.19-1710.6) | -4.15 (-4.93--3.37) | -3.62 (-4.26--2.98) |
| Southern Sub-Saharan Africa | 468446 (384345-578493) | 523753 (421079-652901) | 1454.1 (1204.22-1773.42) | 1649.96 (1322.03-2072.13) | 367431 (291542-456433) | 499094 (390630-633941) | 945.82 (756.55-1172.61) | 1234.96 (971.27-1560.8) | -0.93 (-1.1--0.76) | -0.4 (-0.61--0.18) |
| Western Sub-Saharan Africa | 2966599 (2382667-3809743) | 3272040 (2578211-4265787) | 1917.31 (1549.59-2421.87) | 2383.13 (1904.73-3054.19) | 2767972 (2095642-3651139) | 3852718 (2820648-5119601) | 841 (640.19-1117.6) | 1353.31 (983.95-1819.65) | -2.66 (-2.77--2.56) | -1.84 (-1.91--1.78) |

## 1.2 Supplementary Figures

## Figure S1 A: ASPR in 1990-2021, EAPC of prevalence, percentage change in Nutritional Deficiencies prevalence case.


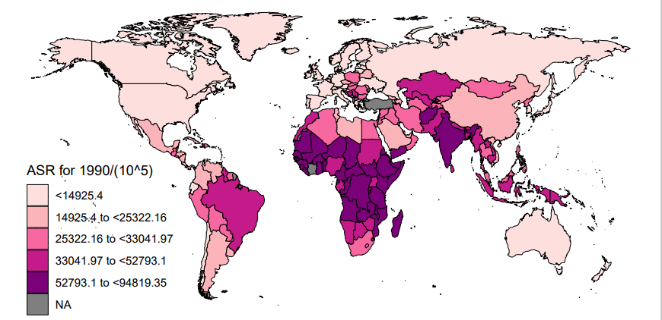

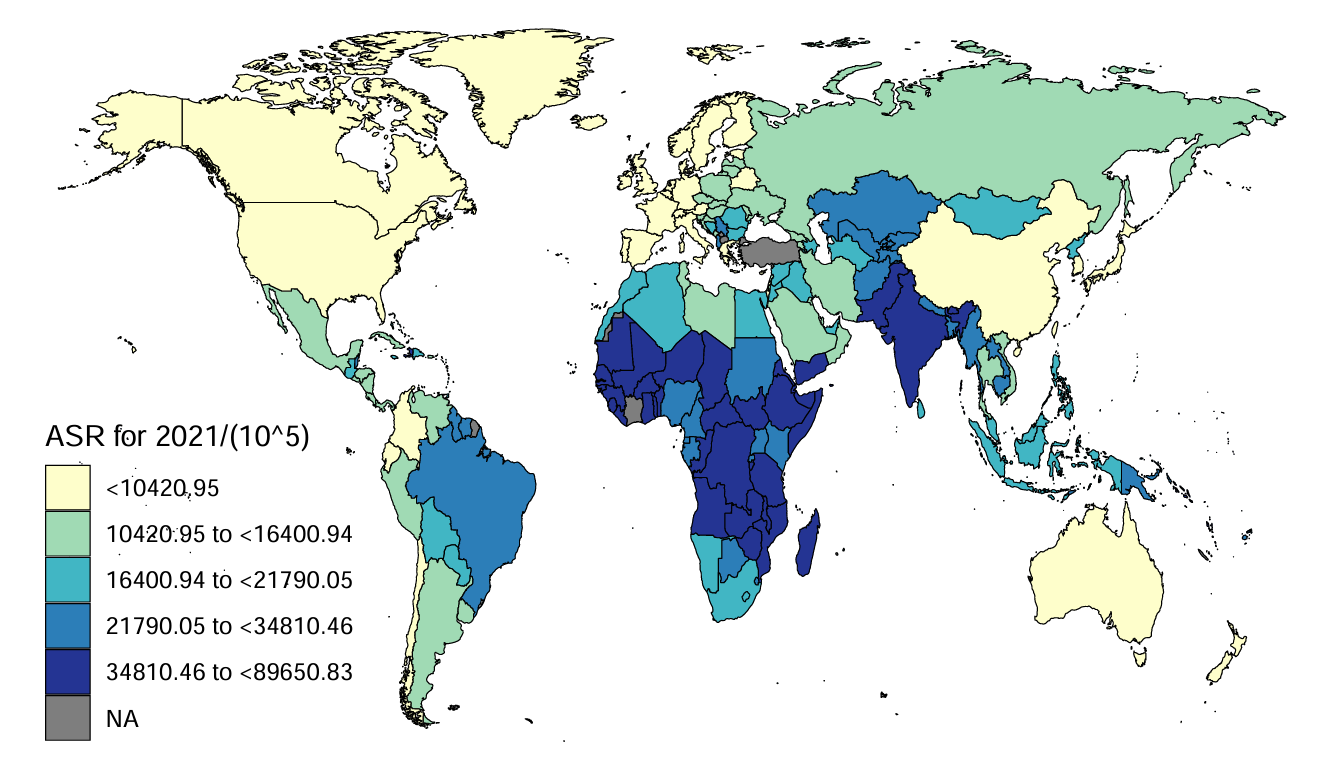


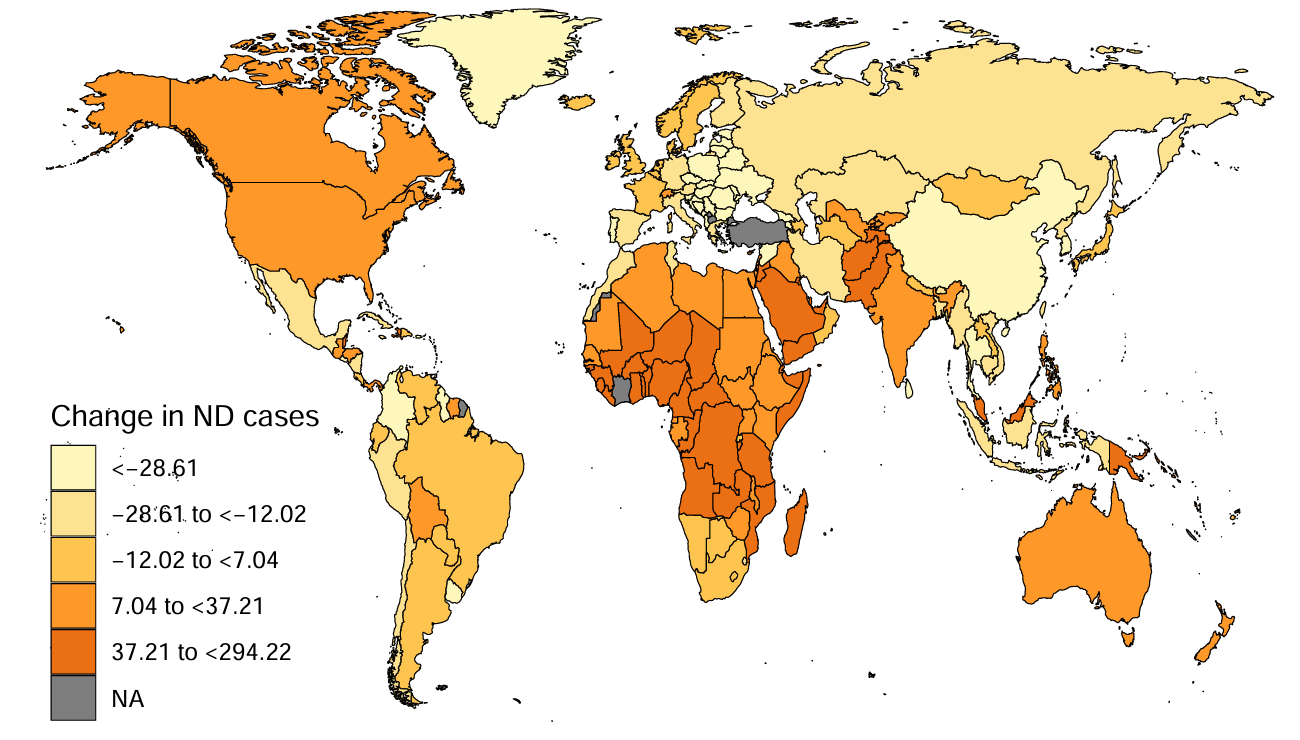

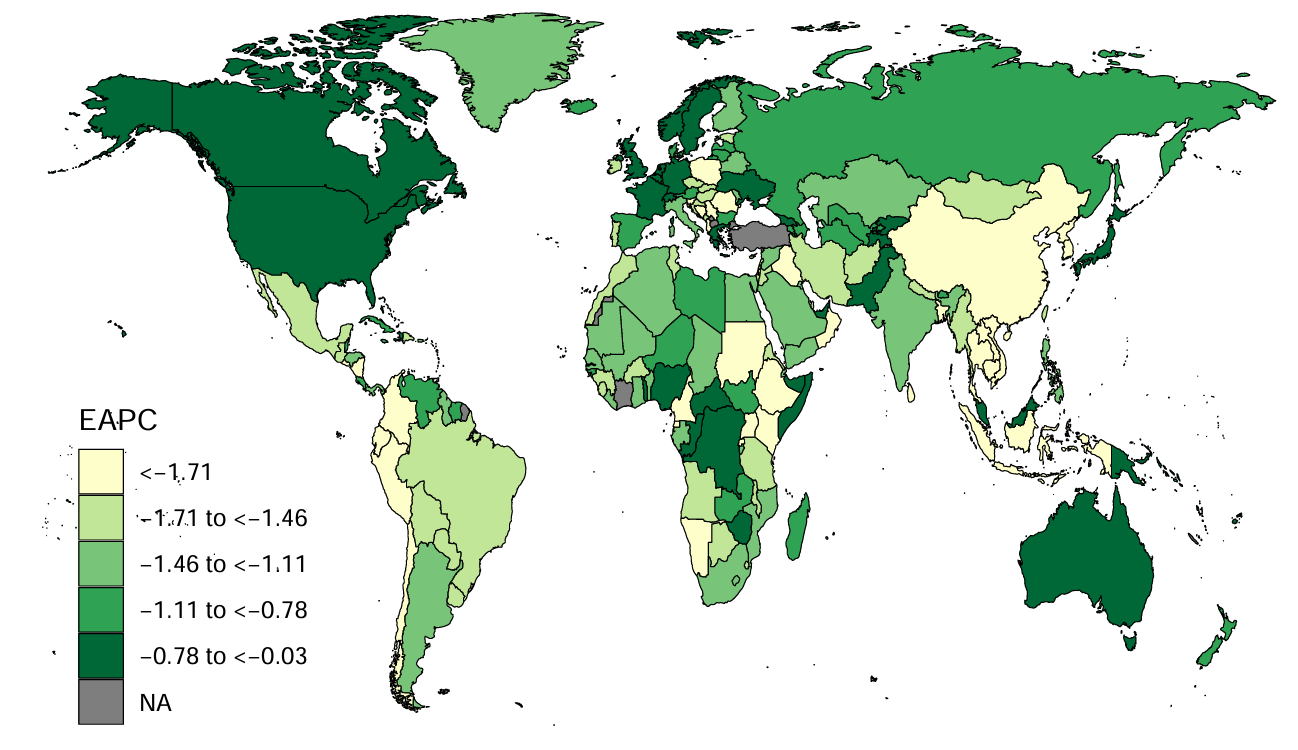


Global GBD and temporal trends of Nutritional Deficiencies prevalence globally. ASR: age standardized rate; EAPC: estimated annual percentage change; GBD: global burden of disease. (a) The ASR per 100,000 people in 1990; (b) The ASR per 100,000 people in 2021; (c) The change in cancer cases; (d) EAPC in different countries or territories.

## Figure S1 B: ASMR in 1990-2021, EAPC of deaths, percentage change in Nutritional Deficiencies deaths case.


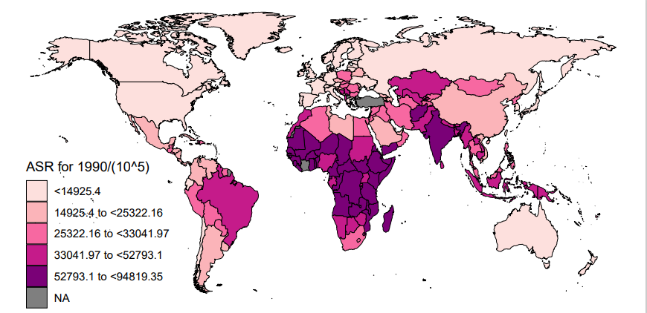

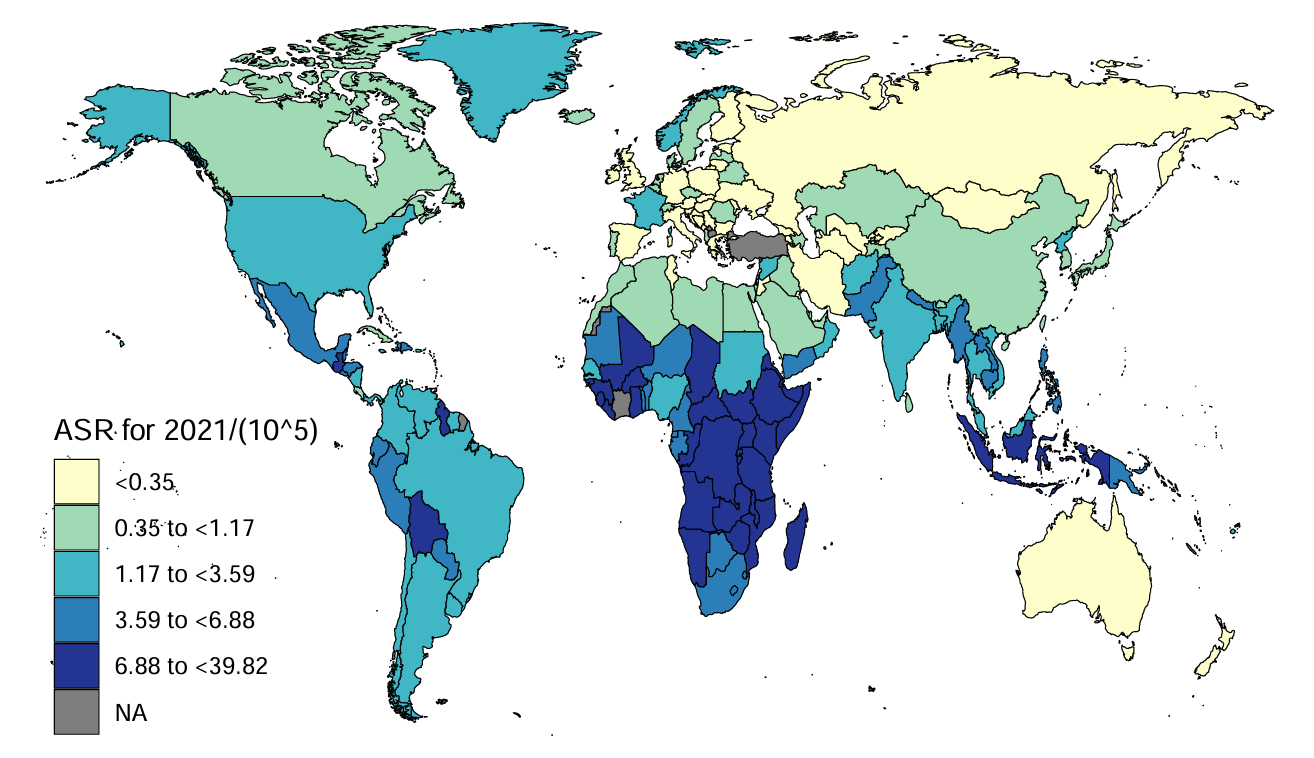


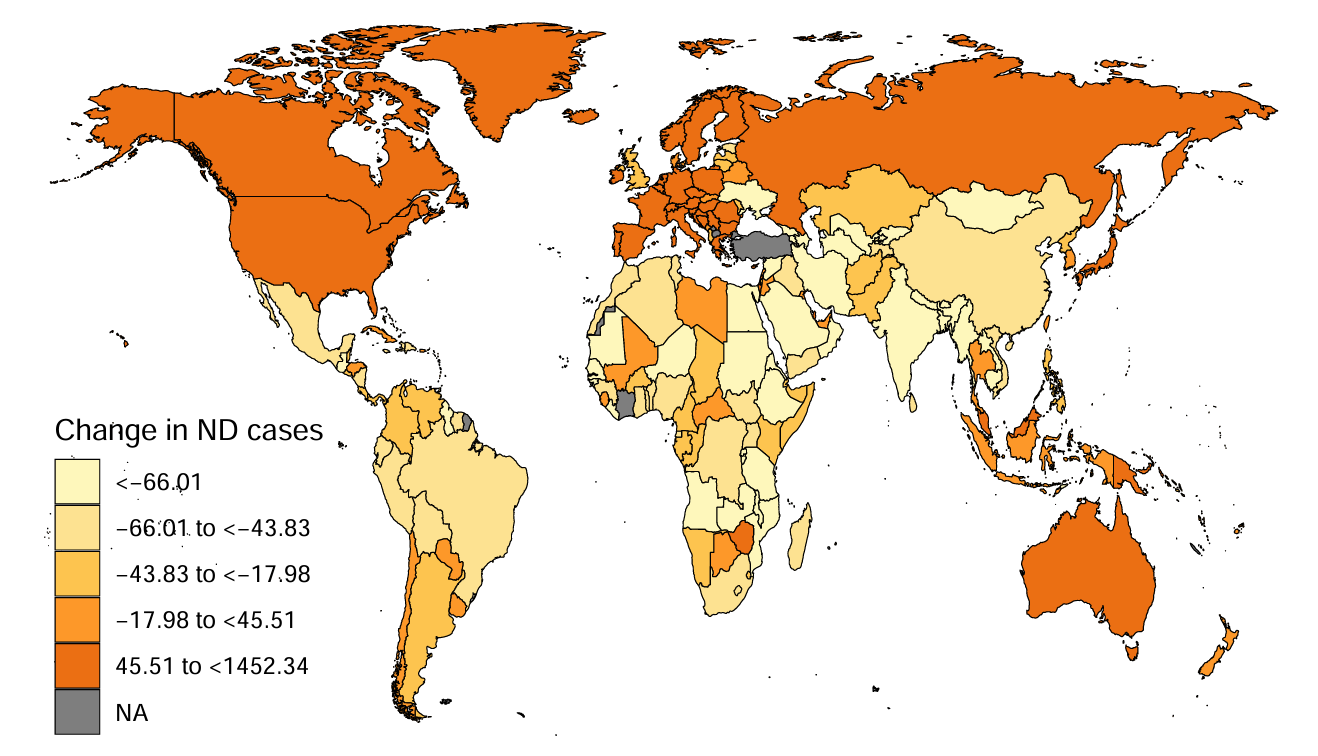

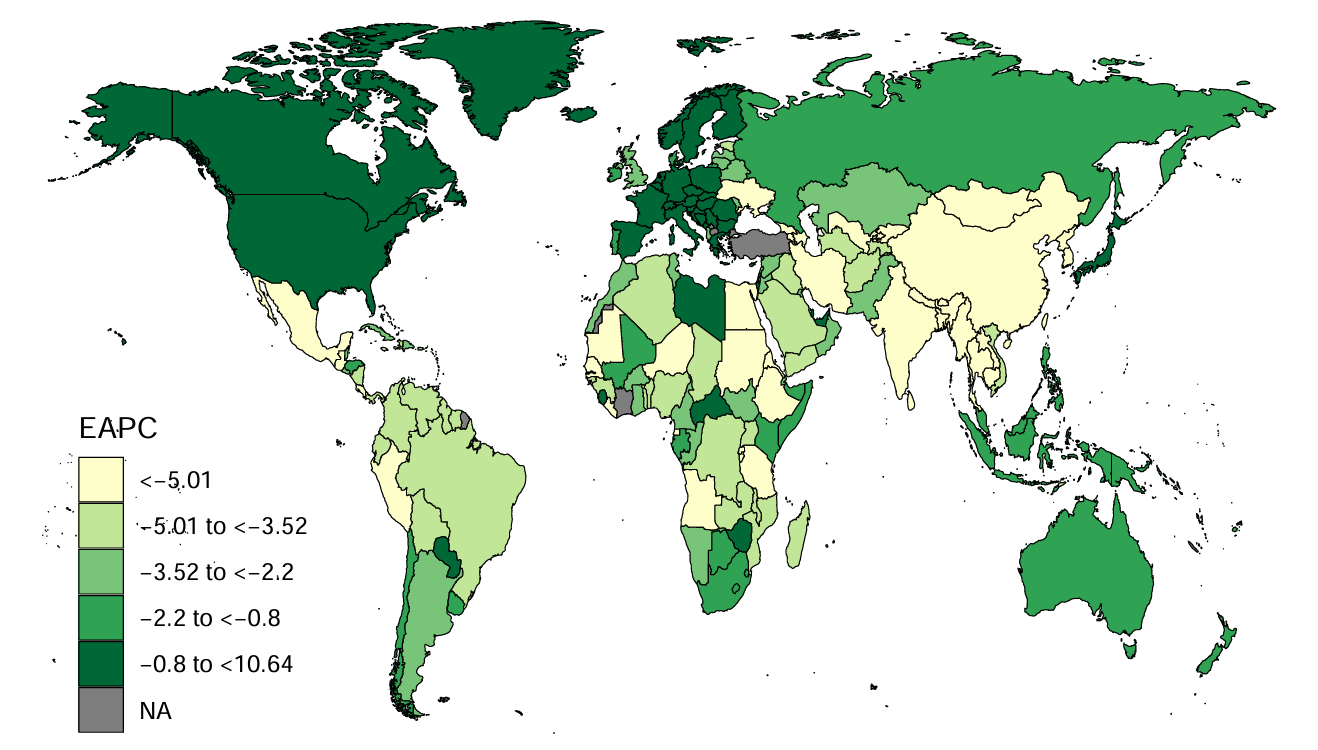


Global GBD and temporal trends of Nutritional Deficiencies deaths globally. ASR: age standardized rate; EAPC: estimated annual percentage change; GBD: global burden of disease. (a) The ASR per 100,000 people in 1990; (b) The ASR per 100,000 people in 2021; (c) The change in cancer cases; (d) EAPC in different countries or territories.

## Figure S1 C: ASR DALYs in 1990-2021, EAPC of DALYs, percentage change in Nutritional Deficiencies DALYs case.


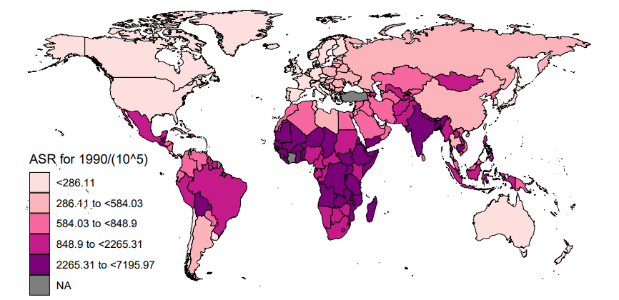

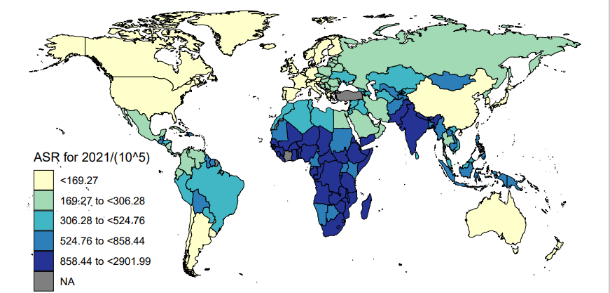


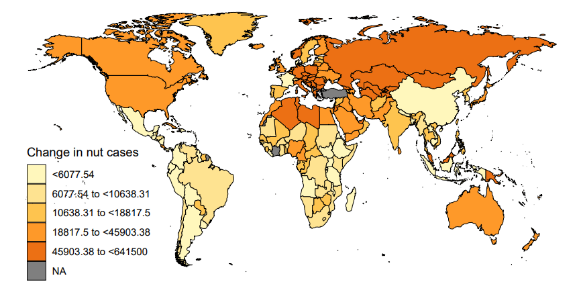

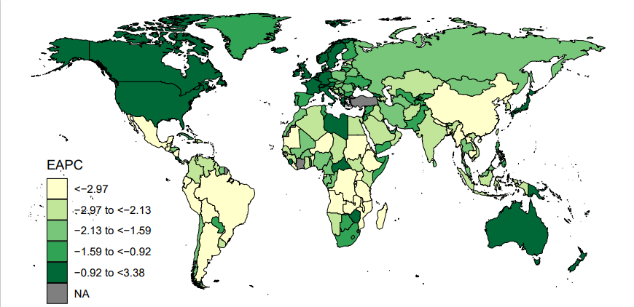


Global GBD and temporal trends of Nutritional Deficiencies DALYs globally. ASR: age standardized rate; EAPC: estimated annual percentage change; GBD: global burden of disease. (a) The ASR per 100,000 people in 1990; (b) The ASR per 100,000 people in 2021; (c) The change in cancer cases; (d) EAPC in different countries or territories.

## Figure S2 A: Prevalence rate per 100,000 populations in 1990 and 2021.


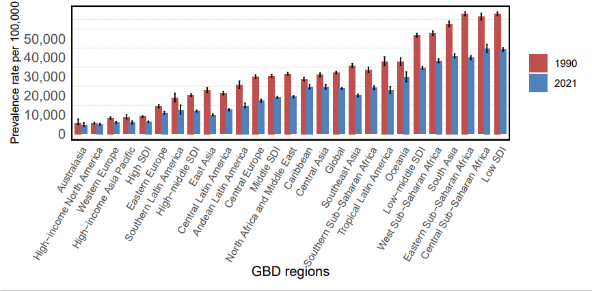


## Figure S2 B: Deaths rate per 100,000 populations in 1990 and 2021.


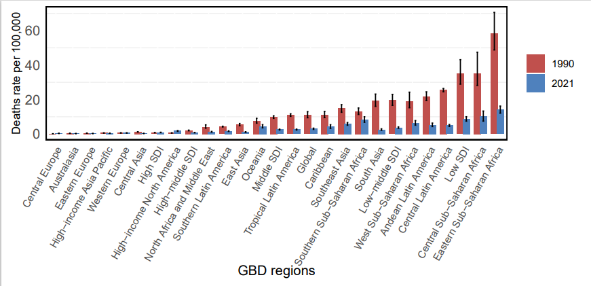


## Figure S2 C: DALYs rate per 100,000 populations in 1990 and 2021


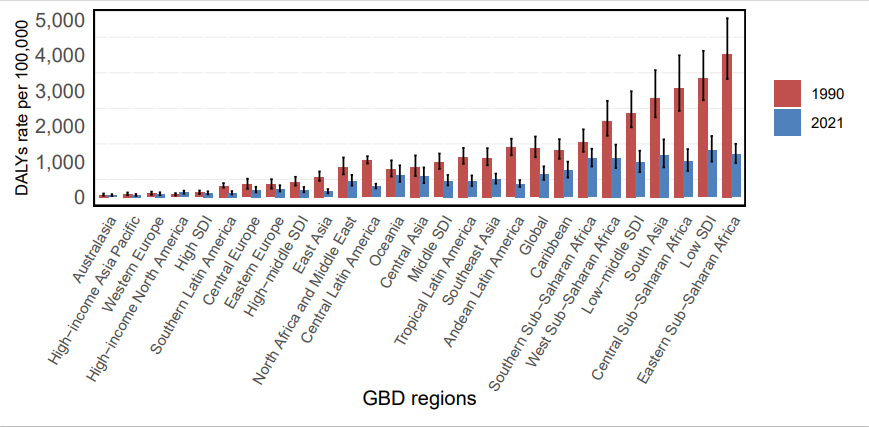


## Figure S3 A：Joinpoint regression analysis of age-standardized Prevalence rate for Nutritional Deficiencies in global and different SDI from 1990 to 2021.


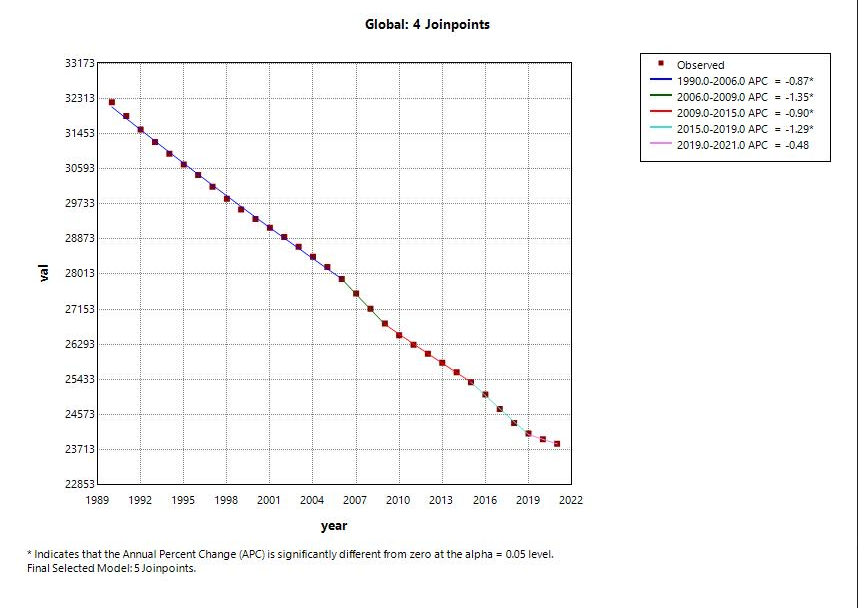

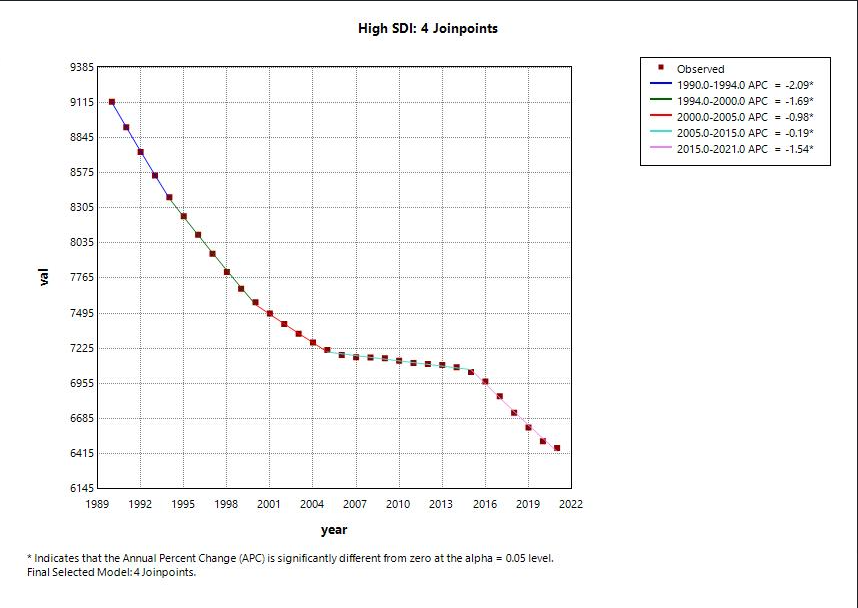

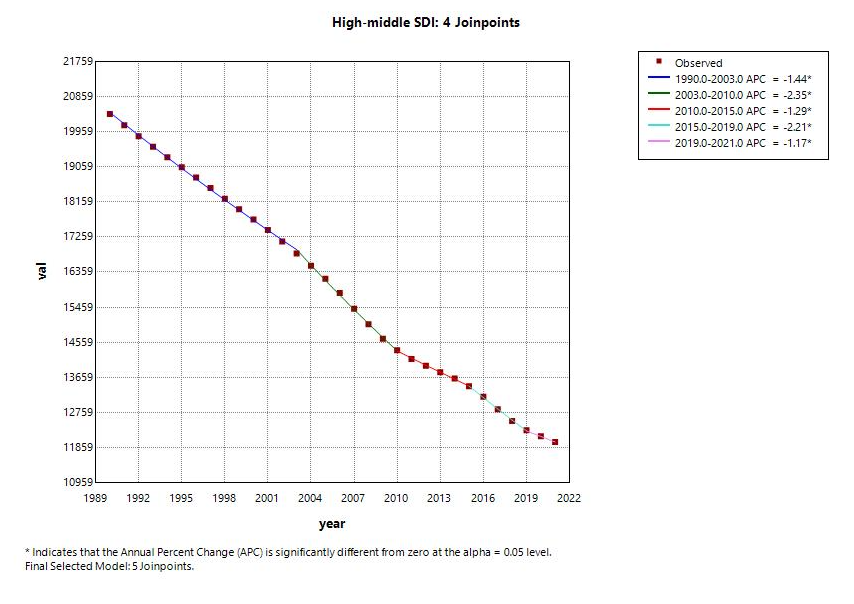

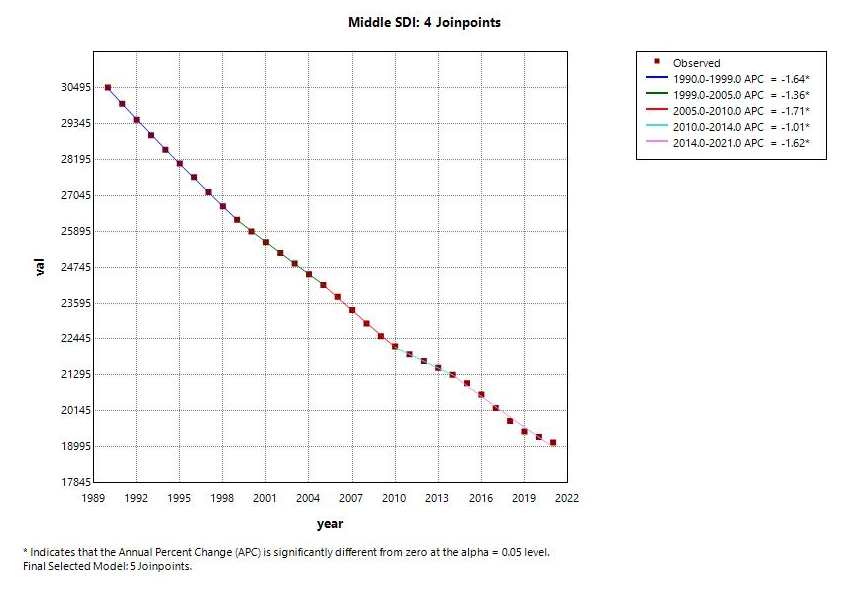

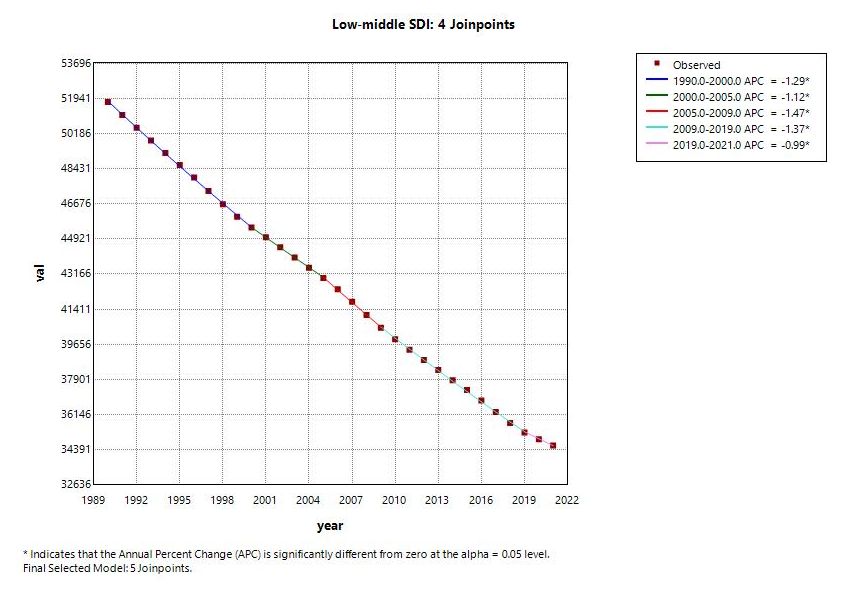

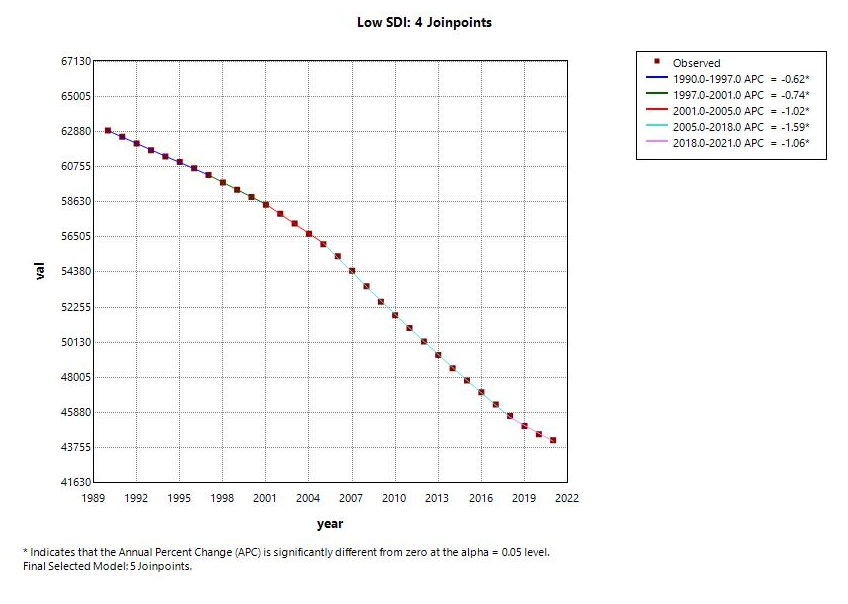


## Figure S3 B: Joinpoint regression analysis of age-standardized Deaths rate for Nutritional Deficiencies in global and different SDI from 1990 to 2021.


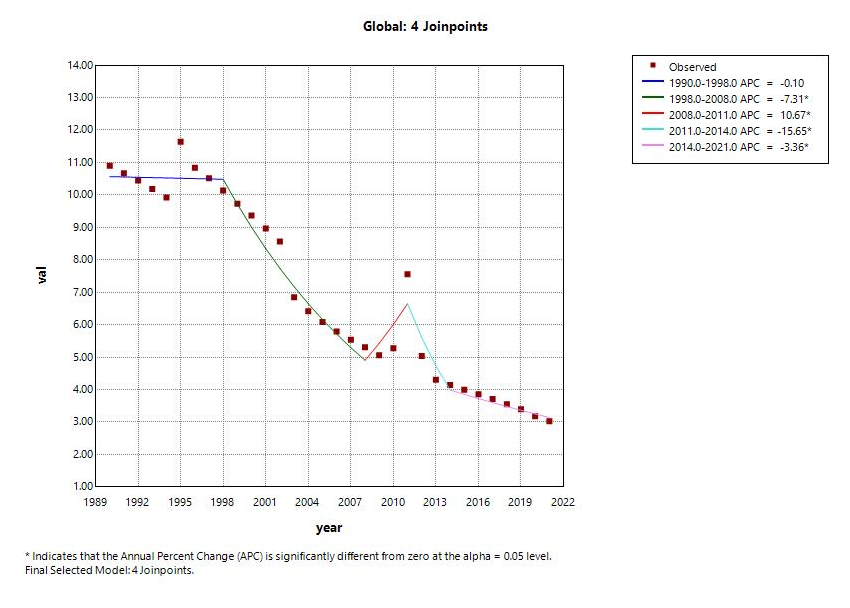

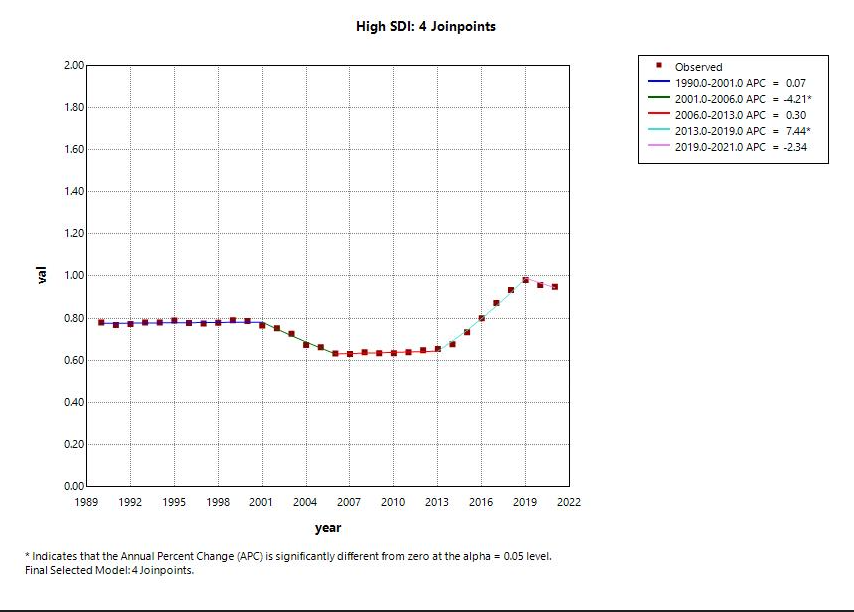

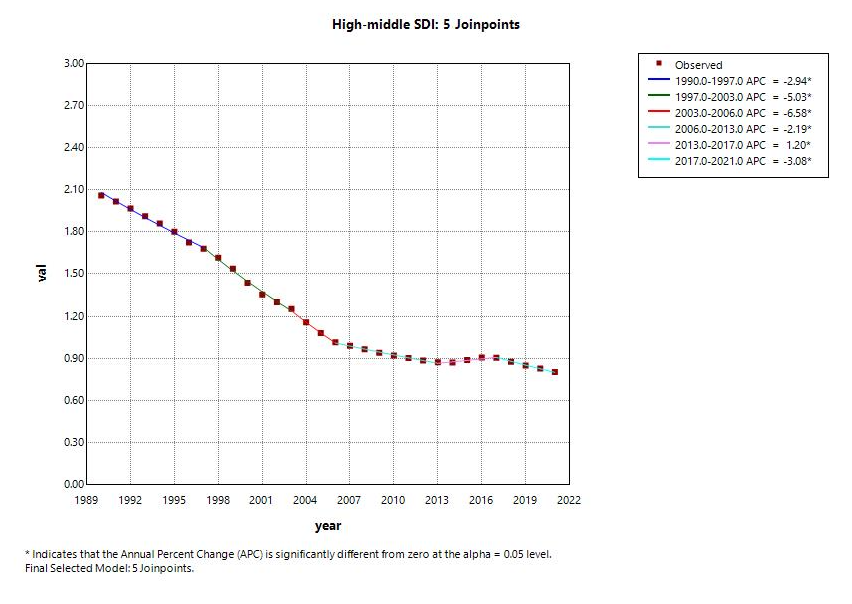

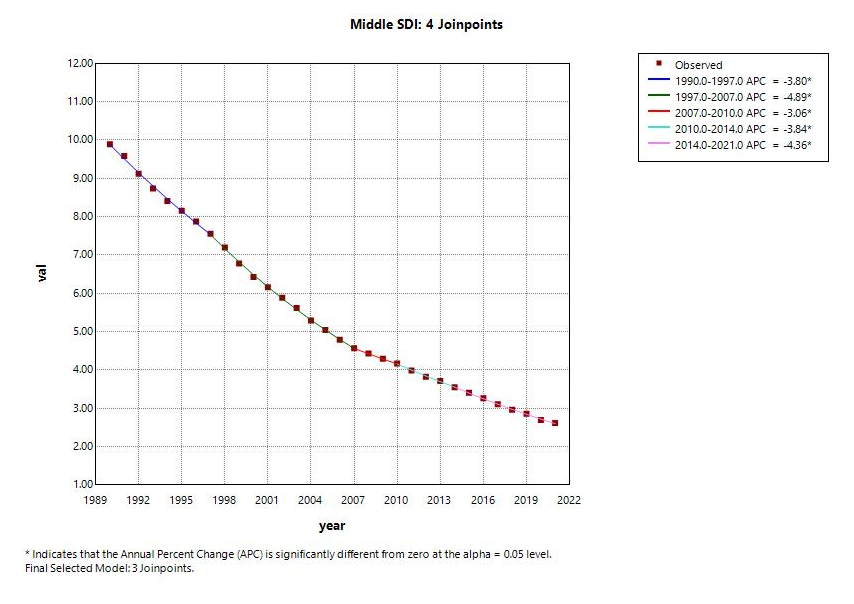

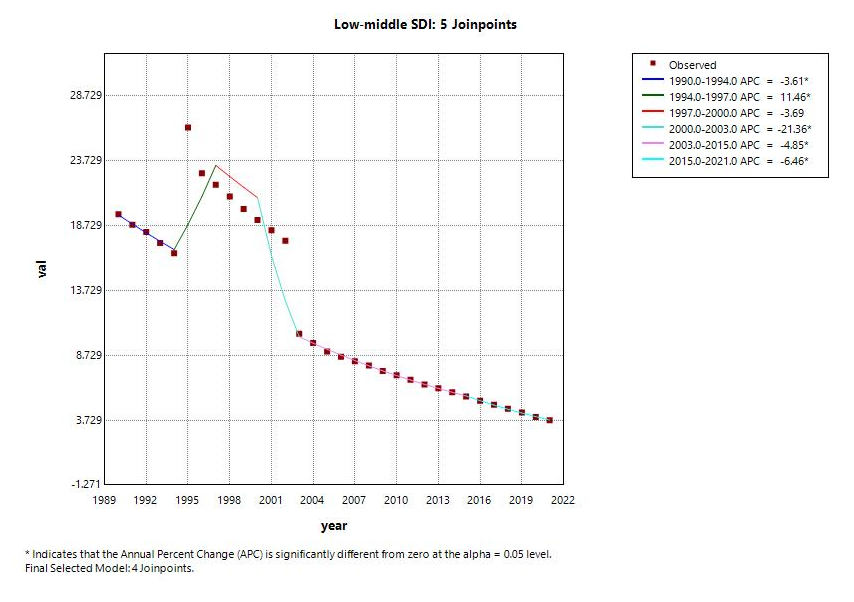

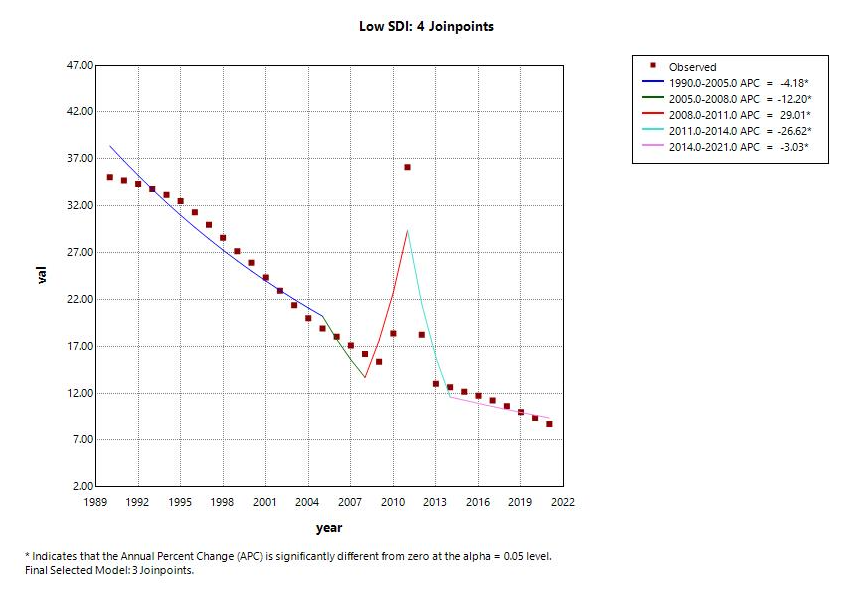


## Figure S3 C: Joinpoint regression analysis of age-standardized DALYS：rate for Nutritional Deficiencies in global and different SDI from 1990 to 2021.


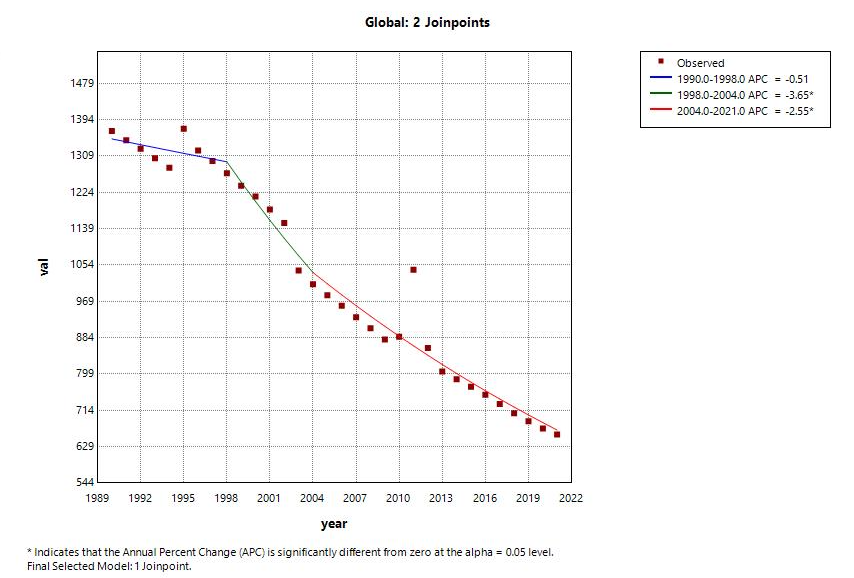

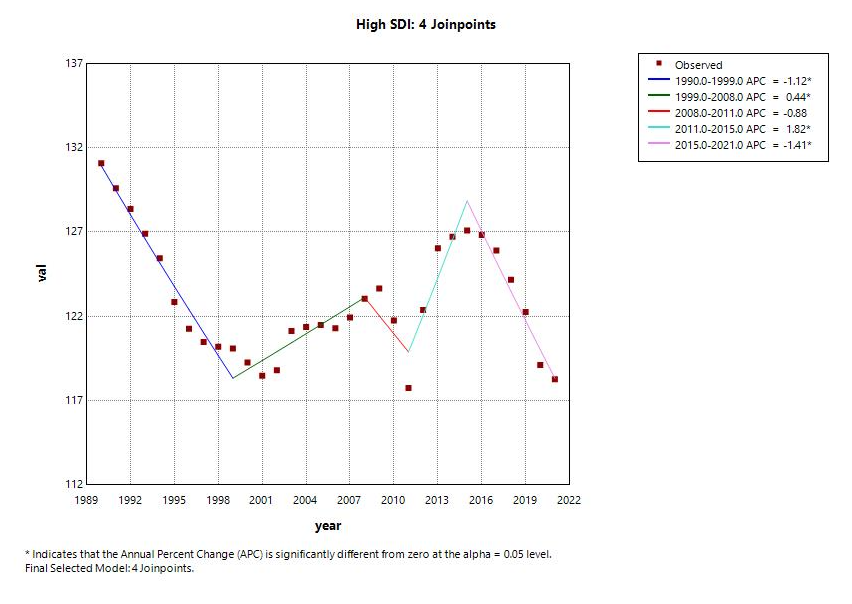

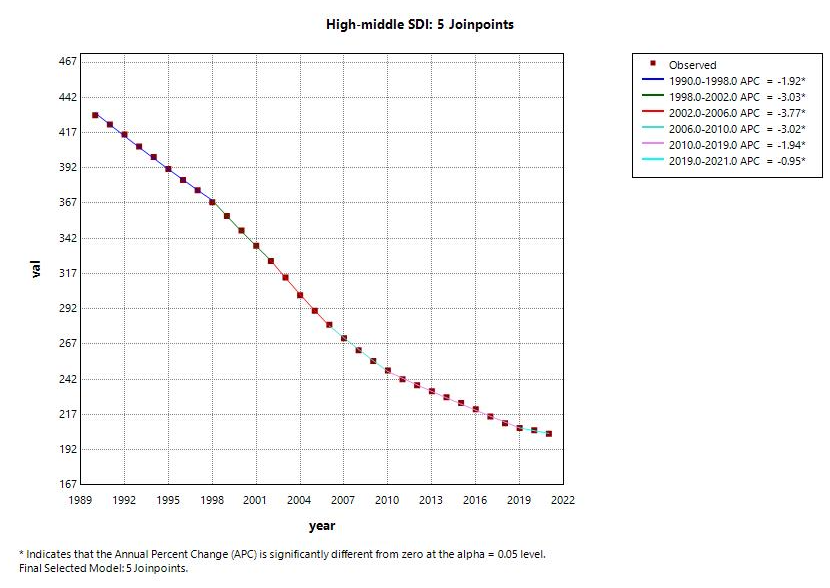

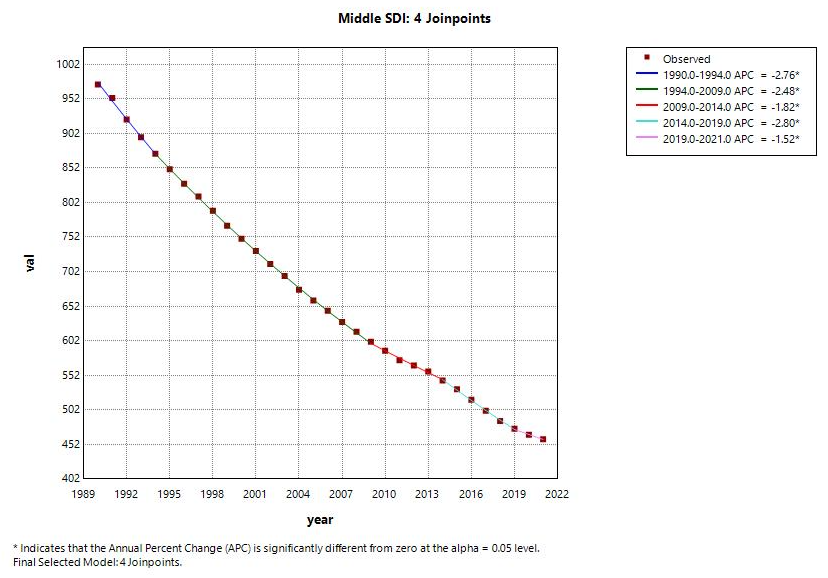

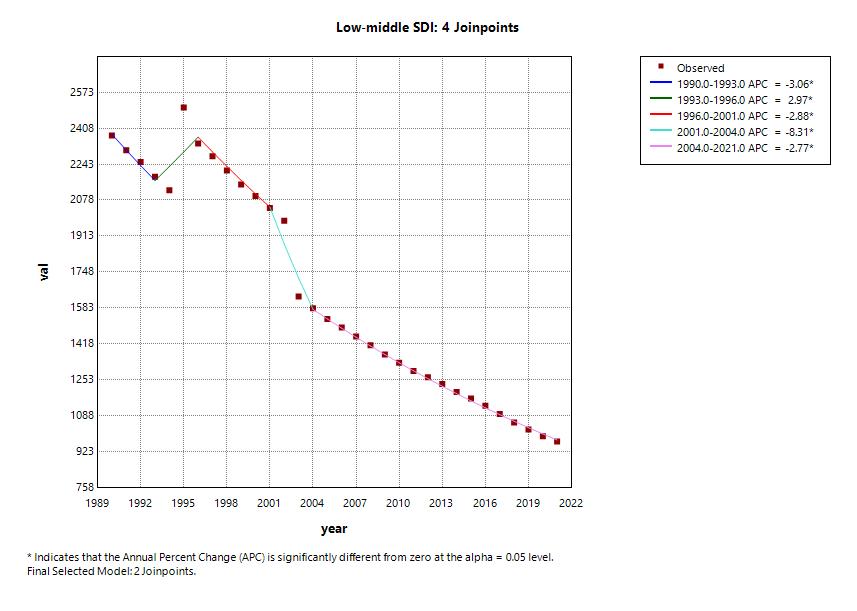

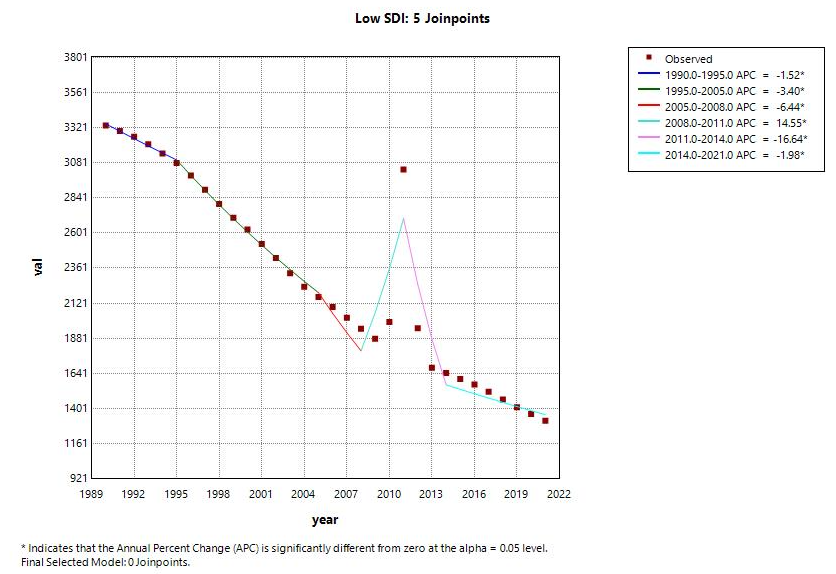


## Figure S5: ASPR, ASMR, age standardized DALYs for 22 GBD regions from 1990 to 2021


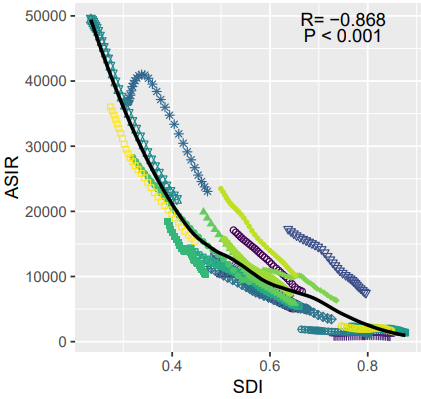

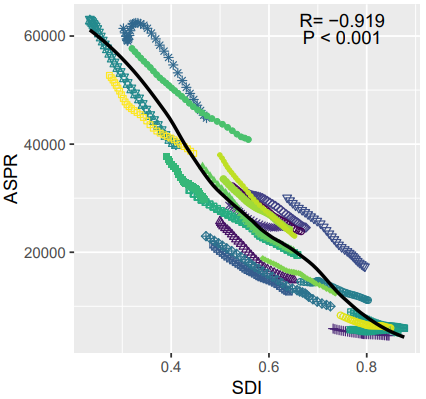

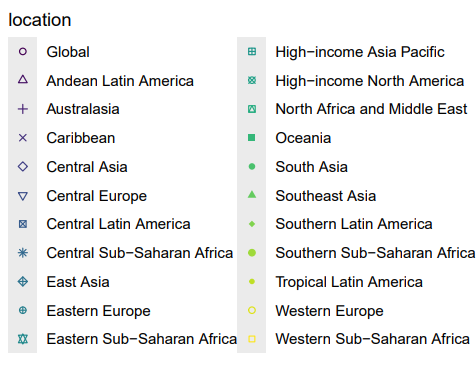


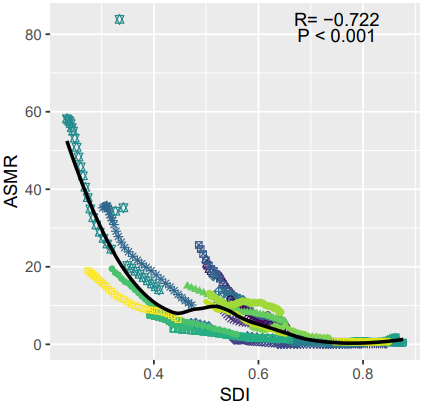

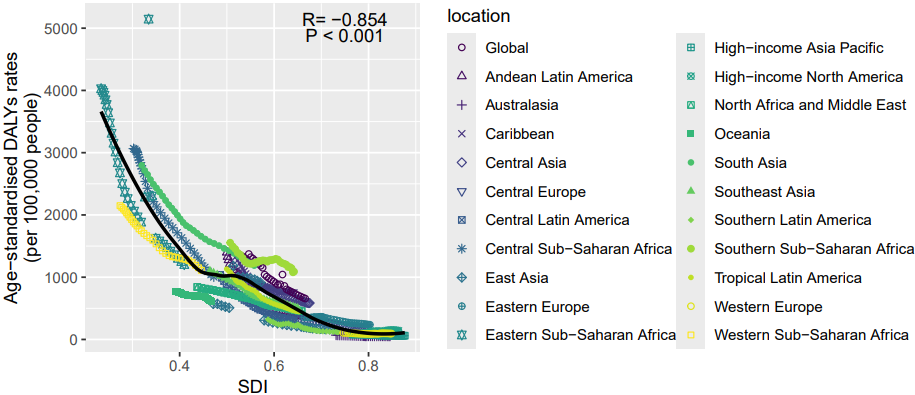


## Figure S6: ASPR, ASMR, age standardized DALYs for 204 countries and regions from 1990 to 2021


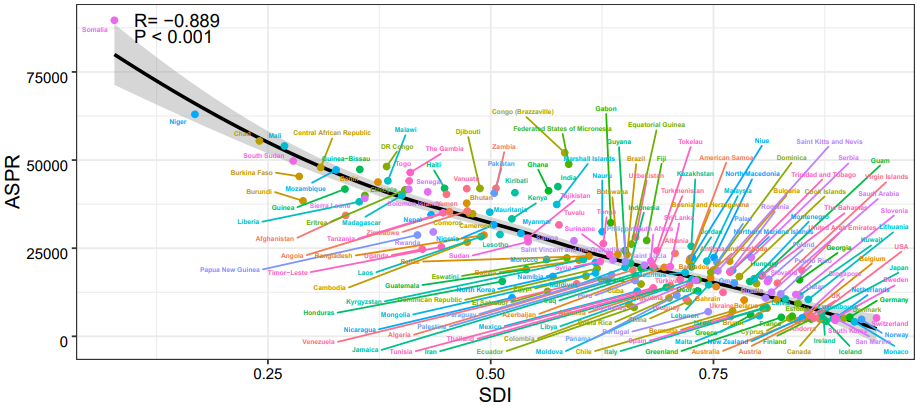

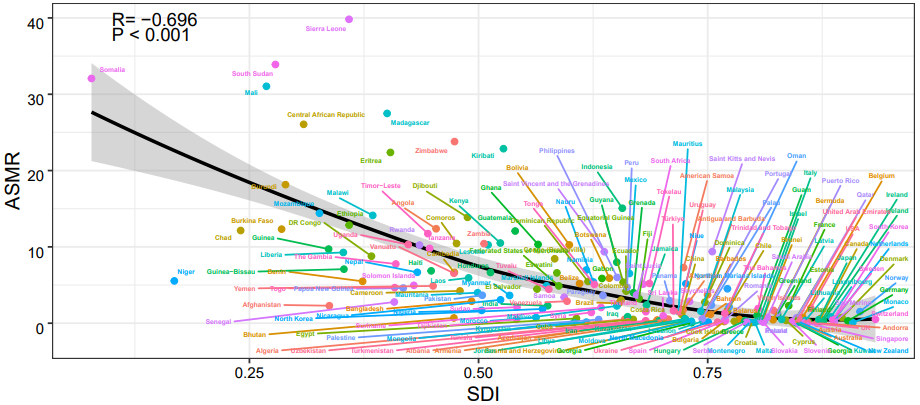

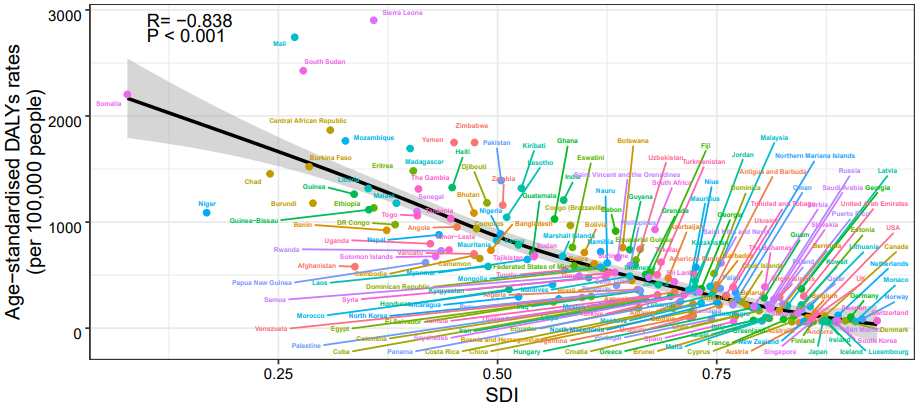

Supplement: Supplementary file 1 [file Data_Sheet_1.docx]
